# Denoising Low‐Power CEST Imaging Using a Deep Learning Approach With a Dual‐Power Feature Preparation Strategy

**DOI:** 10.1002/mrm.70124

**Published:** 2025-10-13

**Authors:** Yashwant Kurmi, Malvika Viswanathan, Leqi Yin, You Chen, Xiaoyu Jiang, Junzhong Xu, Zhongliang Zu

**Affiliations:** ^1^ Vanderbilt University Institute of Imaging Science Vanderbilt University Medical Center Nashville, TN USA; ^2^ Department of Radiology and Radiological Sciences Vanderbilt University Medical Center Nashville, TN USA; ^3^ Department of Biomedical Engineering Vanderbilt University Nashville, TN USA; ^4^ School of Engineering Vanderbilt University Nashville, TN USA; ^5^ Department of Biomedical Informatics Vanderbilt University Medical Center Nashville, TN USA; ^6^ Department of Computer Science Vanderbilt University Nashville, TN USA; ^7^ Department of Physics and Astronomy Vanderbilt University Nashville, TN USA

**Keywords:** chemical exchange saturation transfer (CEST), contrast‐to‐noise ratio (CNR), deep learning (DL), Lorentzian difference (LD) analysis, signal‐to‐noise ratio (SNR)

## Abstract

**Purpose:** Low‐power (LP) chemical exchange saturation transfer (CEST) Z‐spectra have significantly reduced confounding effects and enhanced peak resolvability, thereby improving the observation and quantification of various CEST effects. However, LP Z‐spectra suffer greatly from reduced contrast‐to‐noise ratio (CNR). This study aims to develop a dual‐power feature preparation for an autoencoder‐based deep learning approach (DPDL), for denoising LP Z‐spectra. This method leverages the high CNR of higher saturation power and the enhanced peak resolvability of low saturation power.

**Methods:** The DPDL model was trained on simulated CEST data, validated on both simulated and BSA phantoms, and then applied to measured data from rat brain and leg muscles at 4.7T. A Lorentzian difference (LD) analysis was used to quantify various CEST effects. Several evaluation metrics, including peak signal‐to‐noise ratio (PSNR), were used to assess the denoising performance. To demonstrate the advantage of the DPDL method, it was compared with the autoencoder‐based method without feature preparation and various existing denoising methods that utilized a single LP Z‐spectrum with two averages as input, thereby ensuring equivalent acquisition times.

**Results:** In phantom experiments, the DPDL method demonstrated higher PSNR than existing denoising techniques, validating our approach. In animal experiments, the DPDL method showed improved image quality, outperforming existing denoising techniques. Additionally, several peaks from major tissue components in both brain and muscle were revealed on the LP Z‐spectrum.

**Conclusion:** The superior denoising performance using the DPDL for LP CEST imaging can enhance the isolation of various pools, thereby improving CEST applications, particularly at low fields.

## 1 Introduction

Chemical exchange saturation transfer (CEST) is an advanced molecular imaging technique that offers enhanced detection sensitivity to solute metabolites [1, 2, 3, 4, 5, 6, 7, 8, 9]. By selectively saturating exchangeable protons for several seconds and then measuring the subsequent decrease in water signal due to cumulative exchange effects, CEST indirectly detects solute molecules. One prominent CEST effect is amide proton transfer (APT) at 3.5 ppm, which reflects mobile proteins [10] and has demonstrated potential in diagnosing multiple diseases [10]. Additionally, guanidine and phosphocreatine (PCr) CEST effects occur at 2 ppm and 2.6 ppm [11, 12, 13, 14, 15, 16], respectively, and can provide insights into muscle creatine kinase reactions. Nuclear Overhauser enhancement (NOE)‐mediated saturation transfer effects at −1.6 ppm [17, 18] and −3.5 ppm [19, 20, 21], referred to as NOE(−1.6) and NOE(−3.5), are significant up‐field effects in brain that arise from macromolecules including phospholipids and proteins. These various CEST effects offer valuable information about biochemical processes.

However, CEST is generally less specific to target molecules, primarily due to the broad nature of some CEST peaks, which often lead to significant overlap. For instance, the amine CEST effect at 3 ppm [22, 23], which belongs to the fast exchange regime, exhibits a very broad peak that overlaps with nearly all downfield CEST effects. Additionally, substantial confounding background effects such as direct water saturation (DS) and magnetization transfer (MT) further complicate the interpretation of CEST data. The DS effect is more pronounced at low magnetic fields, making some CEST effects observable only at high fields. To address these challenges, using lower saturation powers, especially at low fields, has been suggested [14]. Lower power narrows the CEST peaks, making them easier to resolve. It also significantly reduces the amine CEST, DS, and MT effects, as their amplitudes roughly depend on the square of the saturation amplitude [24, 25, 26]. In contrast, the dependence of slow‐intermediate exchange CEST effects on saturation field strength is of low order, so they do not decrease as significantly as the confounding effects. Therefore, some slow‐intermediate exchange CEST effects that cannot be easily quantified due to significant overlapping at high power (HP) are expected to be more easily quantified at low‐power (LP). However, this approach comes at the cost of a significantly reduced CEST effect, leading to lower contrast‐to‐noise ratio (CNR) defined as the difference between CEST signals and background signals divided by background noise. The low‐CNR CEST signals can significantly complicate image analysis. Therefore, improving the CNR or low‐signal‐to‐noise ratio (SNR) for the LP CEST imaging is critical for enhancing its applications.

Increasing the number of signal averages can enhance the SNR of CEST signals. However, this method significantly extends the scan time, rendering it impractical for CEST imaging. For example, achieving a twofold increase in SNR necessitates 4 averages, substantially prolonging the scan time and making it less feasible for clinical adoptions. Various post‐processing denoising methods have been also developed. These methods include the nonlocal mean filter and the anisotropic diffusion [27], principal component analysis (PCA) [28], multilinear singular value decomposition‐based (MLSVD) filtering [29, 30], and non‐local mean and coherence‐enhanced diffusion (NLmCED) [27]. Recently, deep learning (DL)‐based CEST denoising methods, such as the denoising chemical exchange saturation transfer network (DECENT) [31], denoising using U‐shaped Network [32], denoising convolutional autoencoder (DCAE) [33], have demonstrated superior noise elimination performance compared to traditional post‐processing methods [34]. These DL‐based approaches typically employ autoencoder networks, which rely on accurately capturing and reconstructing the underlying structure of the data. However, when the SNR or the CNR of the input signal is extremely low, the noise can obscure the signal to such an extent that DL model struggles to learn the true features. This can lead to failure in effectively reconstructing the original signal, resulting in suboptimal denoising performance. While high powers create broad CEST peaks and significant confounding background effects, they also yield greater CEST effects and thus a higher CNR. Here, we propose a dual‐power feature preparation strategy, leveraging the high CNR of higher power and the enhanced peak resolvability of lower power, to enhance the autoencoder denoising for low‐power applications. We term it as a Dual‐Power Deep Learning (DPDL) denoising method.

## 2 Methods

### 2.1 DPDL Architecture

**FIGURE 1:**
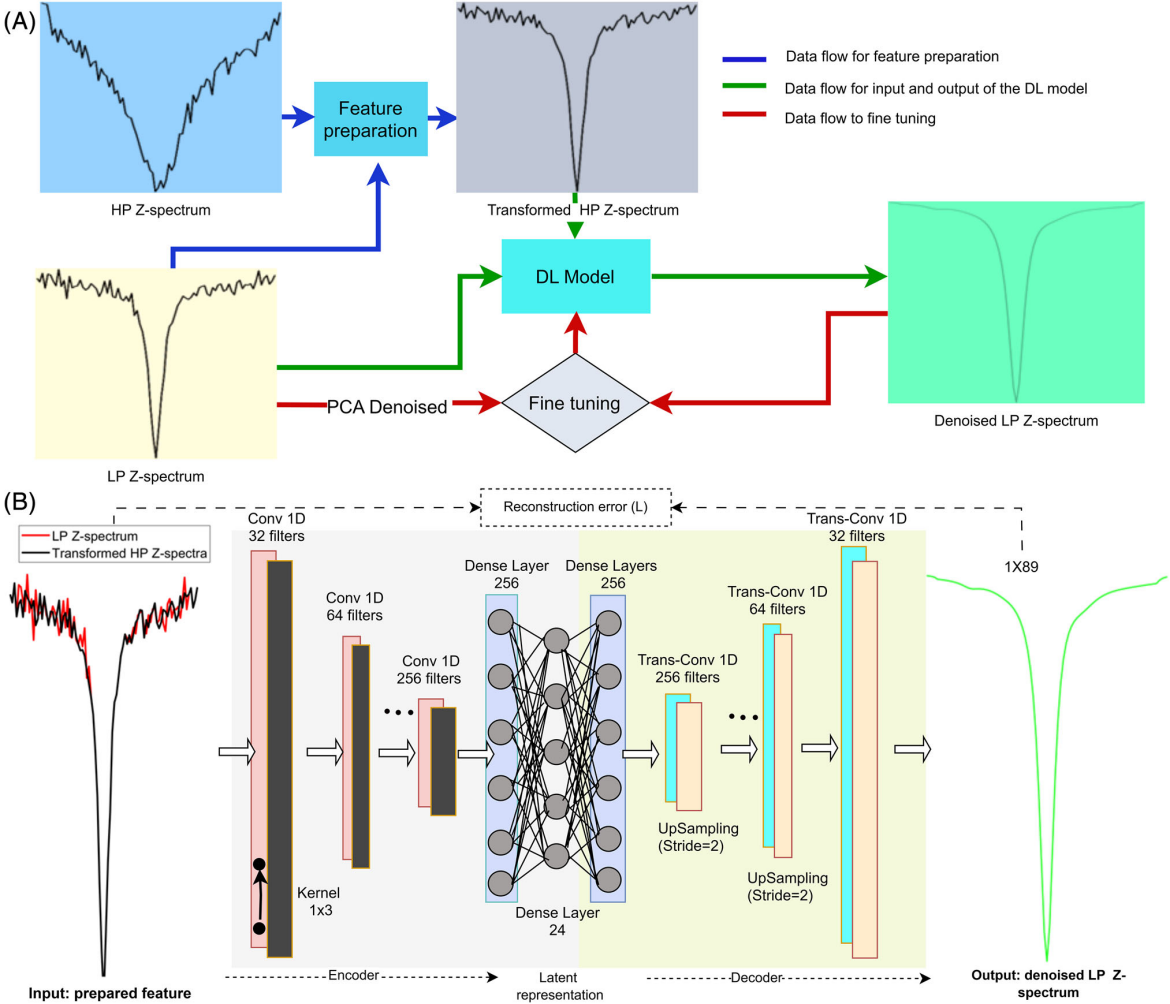
Block diagram illustrating the DPDL architecture, consisting of three stages: Feature preparation, a DL model, and fine‐tuning (A), as well as the autoencoder‐based network within the DL model (B).

Figure 1A illustrates the DPDL architecture, designed to process both LP and HP Z‐spectra. This method involves three stages: feature preparation, an autoencoder‐based DL, and fine‐tuning, ultimately producing a denoised LP Z‐spectrum. In the feature preparation stage, HP Z‐spectrum undergoes several processing steps. First, a Z‐spectral window, containing a certain number of frequency offsets, is defined. Within this window, the mean values of HP CEST signals are subtracted from each HP CEST signal, and these subtracted values are then added to the mean values of corresponding LP CEST signals. This process is applied iteratively across the entire Z‐spectrum, window by window, generating a transformed HP Z‐spectrum that spans the entire frequency range. Figure S1 demonstrates the LP, HP, and the transformed HP Z‐spectrum using this approach, derived from a modified two‐pool (amide and water) model simulation that highlights a single CEST peak for clarity. Notably, in the transformed HP Z‐spectrum, the trajectory of each midpoint within a Z‐spectral window aligns with the LP Z‐spectrum while other points retain the slopes of the HP Z‐spectrum. This trajectory alignment effectively minimizes the greater confounding effects and broader peaks inherent in the HP Z‐spectrum, as these features are primarily embedded within the mean values of the respective windows and can be eliminated through subtraction. Consequently, the DL model can effectively learn the data features of the LP Z‐spectrum with reduced interference from the features of the HP Z‐spectrum. Additionally, the slope of a small section of the Z‐spectrum, representing the derivative at each point, can reveal its fine structure, such as the CEST dip and the sloping DS curve. A steeper slope signifies a more pronounced change in signal intensity over a given frequency offset range, making it easier to distinguish the signal from noise and allowing for more robust identification of fine structure. Under appropriate powers, the slopes of the HP Z‐spectrum within certain windows in tissues are typically steeper than those of the LP Z‐spectrum, as demonstrated in Figure S2. Given that the slopes of the HP and LP Z‐spectra follow specific relationships that the DL model can learn, the slopes of the HP Z‐spectrum within certain windows can be utilized to enhance the robustness of the corresponding slopes of the LP Z‐spectrum to noise, resulting in denoised LP Z‐spectra. However, using a single Z‐spectral window cannot adequately capture the fine structure at the edges of Z‐spectral window and introduces excessive zig–zag pattern in the transformed Z‐spectrum, which impedes effective DL model training. To address these issues, the second step involves defining two Z‐spectral windows of different lengths and averaging the transformed HP Z‐spectra from these two windows. Figure S1 demonstrates the transformed HP Z‐spectra from two Z‐spectral windows of different lengths. The overlap of two Z‐spectral windows suggests that their average can effectively capture the fine structure of the most CEST signals and reduce the zig–zag pattern. In the third step, two Z‐spectral windows are shifted by half of their lengths, as shown in Fig. S1D and S1E. The transformed HP Z‐spectra from these two Z‐spectral windows with and without the shift are then averaged. This further reduces the zig–zag pattern while preserving the fine structure information and robustness of all CEST signals. Figure S1 illustrates the final transformed HP Z‐spectrum after this three‐step feature preparation. Figure S1 showS the enhanced robustness of the slope in this final transformed HP Z‐spectrum compared to the LP Z‐spectrum. In this study, we refer to this final transformed HP Z‐spectrum simply as the “transformed HP Z‐spectrum”, unless otherwise specified. The lengths of these two Z‐spectral windows were chosen as 1 × 3 and 1 × 5 to ensure sensitivity to the fine structure. The optimization of these lengths can be found in Figure S3.

Both the LP Z‐spectrum and the transformed HP Z‐spectrum are subsequently used as input and processed using the autoencoder‐based DL model. Although the transformed HP Z‐spectrum exhibits a zig–zag pattern, this pattern is not randomly distributed. Instead, it depends on specific powers, sample parameters, and the methods used in the feature preparation. As a result, the DL model can effectively capture and absorb this pattern. Simultaneously, the DL model utilizes the transformed HP Z‐spectrum to guide the enhancement of fine structure features in the LP Z‐spectrum. Consequently, this DL model provides a superior denoising performance for the LP Z‐spectrum. Figure 1B illustrates the architecture of the autoencoder network, which comprises a 1D convolutional layer, ELU activation functions, and max pooling for down‐sampling in the encoder. The decoder performs up‐sampling using a transposed convolutional layer, followed by a 1D convolutional layer and linear activation. The autoencoder's architecture was meticulously designed with the appropriate depth and width to prevent overfitting during training.

After processing by the autoencoder, the predicted outcome, i.e., DPDL‐denoised LP Z‐spectrum (Z_DPDL_LP_), is obtained. While the autoencoder‐based DL model exhibits excellent denoising performance, the predicted results may deviate from the original input LP Z‐spectrum (Z_ori_LP_) due to potential mismatches between the training data and input data, as previously discussed [33]. To mitigate such bias in the predictions, a fine‐tuning step utilizing context learning is applied. This step uses the PCA‐denoised original input LP Z‐spectrum (Z_PCA_ori_LP_) as a reference. The PCA performs denoising by removing high‐frequency components and reconstructing the signal using a reduced number of principal components. Although PCA may not provide optimal denoising when the SNR is very low, it typically introduces less bias compared to other denoising methods. This is because, when the true signal has a low‐rank structure, it can often be well approximated by several principal components. Consequently, Z_PCA_ori_LP_ serves as an effective reference for reducing bias. In this study, we selected 8 most significant principal components for obtaining Z_PCA_ori_LP_. The goal of context learning is to adjust Z_DPDL_LP_ to be close to Z_PCA_ori_LP_, thereby reducing bias. However, it is crucial to ensure that Z_DPDL_LP_ does not fully match Z_PCA_ori_LP_, as this would result in the denoising capability of DPDL becoming like that of PCA, ultimately diminishing the denoising performance of DPDL. To achieve this balance, a fine‐tuning method is employed. Specifically, the mean squared error (MSE) between Z_PCA_ori_LP_ and Z_ori_LP_, termed MSE_REF_, is calculated. This serves as a criterion for determining whether Z_DPDL_LP_ is either too far from or too close to Z_PCA_ori_LP_. Additionally, the MSE between Z_PCA_ori_LP_ and Z_DPDL_LP_ from each iteration, termed MSE_DPDL_, is calculated to represent the bias of the predicted outcome. Furthermore, a difference spectrum between Z_DPDL_LP_ and Z_ori_LP_ is calculated and scaled by multiplying it with either 1.01 or 0.99, depending on whether Z_DPDL_LP_ is too far from or too close to Z_PCA_ori_LP_. This scaled difference spectrum is then used to fine‐tune the predicted outcome by subtracting it from Z_DPDL_LP_, which is then used as input for the next iteration of prediction. During the prediction, if MSE_DPDL_ is found to exceed a predefined threshold, indicating significant bias, context learning is applied. Then if MSE_DPDL_ exceeds MSE_REF_, the multiplication factor ‘1.01’ will be used for fine‐tuning. Consequently, if Z_DPDL_LP_ is higher than Z_PCA_ori_LP_, this fine‐tuning would reduce the input Z‐spectrum and then reduce the predicted outcome in the next iteration. Additionally, if Z_DPDL_LP_ is lower than Z_PCA_ori_LP_, this fine‐tuning would increase the input Z‐spectrum and then increase the predicted outcome in the next iteration. Both would make Z_DPDL_LP_ in the next iteration closer to Z_PCA_ori_LP_. Conversely, if MSE_DPDL_ is smaller than MSE_REF_, indicating the predicted outcome is too close to Z_PCA_ori_LP_, the multiplication factor “0.99” will be used for fine‐tuning. This would make Z_DPDL_LP_ in the next iteration farther from Z_PCA_ori_LP_. This context‐learning continues until MSE_DPDL_ falls below the threshold or until a maximum of five iterations is reached.

### 2.2 Generation of Training Data and DL Model Training

Training data were generated using a four‐pool (solute #1, solute #2, water, and MT) model simulation of Bloch‐McConnell equations. This model was used to ensure that the training data represents generalized features of CEST data, rather than being specific to any tissue. To achieve this, the exchange rates of each solute were systematically varied to encompass a spectrum from slow to fast exchange rates, effectively representing all types of exchangeable pools and producing CEST peaks with varying widths. Furthermore, the frequency offsets of the two solute pools were independently adjusted to simulate different degrees of potential pool overlap, thereby generating diverse fine structures. This approach enables the DL model to learn to identify fine structures from noisy data in a general context, rather than focusing on tissue‐specific structures with fixed offsets and widths. The MT pool was included as it is present in most tissues. CEST Z‐spectra were simulated with the same frequency offsets (Δω) and saturation strengths (ω_1_) as those in the measurements. To simulate real‐world conditions, B_0_ and B_1_ shifts were introduced to the Z‐spectra. Specifically, Δω was shifted by 0, ±0.2 and ±0.4 ppm, and ω_1_ was scaled by factors of 0.8, 0.9, 1, 1.1, and 1.2. Gaussian noise at levels of 2.5% and 3% of water signal was added to the simulated Z‐spectra, respectively, to create the training data. This resulted in the generation of a total of 398 131 200 Z‐spectra pairs for two powers, by varying the sample parameters listed in Table S1, two noise levels, five B_0_ shifts, and five B_1_ shifts. Notably, the noise added to both LP and HP Z‐spectra maintained the same level. This setting is based on the analysis of the sources of CEST Z‐spectral noise, which primarily arise from the thermal noise of the receiver coils, the stability of the system, and physiological noise—all of which are independent of the saturation power. To validate this, repetitive acquisition of CEST signals at specific frequency offsets with two powers of 0.25 and 1 μT was performed, as shown in Figure S4, demonstrating that the noise level is comparable for both powers. Moreover, noise was generated for each Z‐spectrum separately to simulate different acquisition scenarios. Clean counterparts for the LP Z‐spectra, with no B_0_ and B_1_ shifts, were used as reference data. In this study, 0.25 μT was used for the low power, and 1 μT was used for the high power, except where otherwise noted. 80% of the data was allocated for training purposes, while the remaining 20% was reserved for validation. The learning rate was set at 1 × 10^−4^, with 100 epochs for each sample with Adam optimizer.

### 2.3 Simulation Validation

To validate our method, a digital phantom mimicking brain was generated from the simulation of Bloch‐McConnell equations. Specifically, a seven‐pool (amide at 3.5 ppm, amine at 3 ppm, guanidine at 2 ppm, water, NOE at −1.6 and −3.5 ppm, and MT) model simulation was performed. The sample parameters were listed in Table S2. The sequence parameters were the same as those in the measurements. Additionally, B_0_ shifts of 0 and ±0.3 ppm as well as B_1_ shifts of 0.85, 1, and 1.15 were added to these simulations. Gaussian noise at a level of 2.5% was added to the Z‐spectrum of each voxel in the digital phantom. A 25 × 25 matrix with varied sample parameters was generated. To avoid the edge effects, a 23 × 23 matrix was selected for the digital phantom by removing voxels near the edge.

### 2.4 Phantom Validation

To further validate our method, a bovine serum albumin (BSA) phantom was prepared by dissolving 10% (w/w) BSA to phosphate‐buffered saline with a pH of 7 and measured at room temperature. The testing data was acquired using a slice thickness of 1.5 mm with a single average, whereas ground truth (GT) data were obtained using a slice thickness of 10 mm with 4 averages.

### 2.5 Animal Preparation

To evaluate the DPDL performance in real tissues such as brain and muscle, six rats bearing 9 L tumor (#1–#6) and six healthy rats (#1–#6) were involved, respectively. For the tumor induction in brain, each rat was injected with 1 × 10^5^ 9 L glioblastoma cells and imaged after 15–20 days. All rats were anesthetized using a mixture of 2% isoflurane and 98% oxygen. Respiration was monitored, and a constant rectal temperature of 37°C was maintained throughout the experiments. Images were acquired from the brains in the tumor rats and the hindlimb muscle in the healthy rats. All experiments were approved by Vanderbilt University Medical Center.

### 2.6 MRI

Z‐spectra were measured with Δω from ±10 to ±6.25 ppm with a step size of 1.25 ppm and −5 to 5 ppm with a step size of 0.125 ppm at 4.7 T. These spectra were measured under two different saturation strengths of 0.25 μT and 1 μT. Control images were acquired with Δω at 500 ppm. The CEST sequence consisted of a 5‐s continuous wave saturation pulse, followed by a single‐shot spin‐echo echo planar imaging and a 2‐s recovery period. The water longitudinal relaxation time (T_1w_ = 1/R_1w_) was obtained using an inversion recovery sequence [35]. The image matrix size was 64 × 64, the field of view was 30 × 30 mm^2^ for brain and 35 × 35 mm^2^ for muscle, slice thickness was 2 mm, and the number of averages was 1. Lipid signals in muscle, prominent around −3.5 ppm, can complicate the accurate quantification of the NOE effect [36]. To effectively mitigate these lipid signals, a lipid suppression technique involving a 90‐degree excitation pulse at −3.5 ppm followed by a spoiler gradient is typically employed. In this study, we applied this method specifically to muscle tissue. All measurements were carried out on a Varian 4.7 T magnet with a 38 mm RF receiver coil.

### 2.7 Evaluation Metrics

MSE, mean absolute error (MAE), peak SNR (PSNR) [31], structural similarity index (SSIM) [31], defined in Equations (S1–S4) respectively, were used to evaluate the denoising performance. A low value of MSE and MAE, or a high value of PSNR and SSIM, indicates superior denoising performance.

### 2.8 Ablation Study and Optimization

To demonstrate the effectiveness of the dual‐power feature preparation strategy compared to a single‐power strategy, we modified the input to the autoencoder network to include only two LP Z‐spectra and compared the evaluation metric values of its DL predicted Z‐spectrum with those obtained from the DPDL‐predicted Z‐spectrum through simulations. Additionally, to assess the effectiveness of the autoencoder network itself, we compared the evaluation metric values of the average of the input LP Z‐spectrum and transformed HP Z‐spectrum with their DPDL‐predicted Z‐spectrum. Averaging the two input Z‐spectra ensures the same acquisition time for a fair comparison. It also provides the intermediate quality of input data before the autoencoder network, highlighting the role of the autoencoder network in enhancing data quality. Furthermore, to assess the effectiveness of feature preparation, we directly input the LP and HP Z‐spectrums into the autoencoder network and compared the evaluation metric values of the predicted results with those of the DPDL‐predicted Z‐spectra. Lastly, to optimize the high power, we evaluated the DPDL‐predicted Z‐spectra using several high‐power strengths: 0.5, 1, 1.5, and 2 μT, and compared the corresponding evaluation metric values. All DL models, configured with various combinations of powers as input data, were retrained on training data simulated with the respective powers.

### 2.9 CEST Quantification and Data Analysis

CEST effects were quantified by a Lorentzian difference (LD) analysis [21, 37], along with an apparent exchange‐dependent relaxation (AREX) metric [38, 39, 40], defined in Equation (1).

(1) $${\text{AREX}}_{\mathrm{LD}}\left(\Delta \omega \right)=\left(\frac{1}{S_{\mathrm{lab}}\left(\Delta \omega \right)}-\frac{1}{S_{\mathrm{Ref}}\left(\Delta \omega \right)}\right)\;R_{1w}$$

where the label signal (S_lab_) represents the measured LP CEST signal, and the reference signal (S_ref_) is estimated using a two‐pool (water and MT) model Lorentzian fitting of the LP Z‐spectrum at specific frequency offset ranges beyond most CEST/NOE effects. In this study, CEST signals ranging from ±10 ppm to ±6.25 ppm and from −0.5 ppm to 0.5 ppm were used for fitting the reference signals. Table S3 provides the starting points and boundaries for the LD analysis. The maps of each CEST effect were generated by selecting the maximum values on the LD fitted spectrum, voxel by voxel, within the following ranges: 3.25 to 3.75 ppm for APT, 2.35 to 2.85 ppm for PCr, 1.75 to 2.25 ppm for guanidine, −1.35 to −1.85 ppm for NOE(−1.6), and −3.25 to −3.75 ppm for NOE(−3.5). ROIs of tumors, contralateral normal tissues, and muscle tissues were delineated from R_1obs_ maps. Student's t‐tests were employed to evaluate the statistical difference between the tumors and normal tissues. A *p* value less than 0.05 or 0.02 was considered to be statistically significant. All the data processing and DL training and prediction were carried out in the MATLAB (R2023b) or python environment, running on a machine with Intel(R) Core(TM) i9‐10 900X CPU@3.70GHz ×20 equipped with 64GB RAM and NVIDIA RTX A4000 GPU with 26.5GB.

### 2.10 State‐Of‐The‐Art Methods

To demonstrate the improved performance of our DPDL method, we compared it with several state‐of‐the‐art denoising methods, including PCA, MLSVD, NLmCED, and DCAE. The PCA method was evaluated using three variations including PCA(7), PCA(8), and PCA(12), which employ 7, 8, and 12 principal components, respectively, to reconstruct denoised Z‐spectra. These existing methods were applied to process the LP CEST signals. Since these existing denoising methods process CEST data using a single power, we utilized two averages for the noisy Z‐spectra processed by these methods to ensure an equivalent acquisition time as used by our DPDL method.

## 3 Results

### 3.1 Demonstration of the Principle of the Dual‐Power Feature Preparation Strategy

**FIGURE 2:**
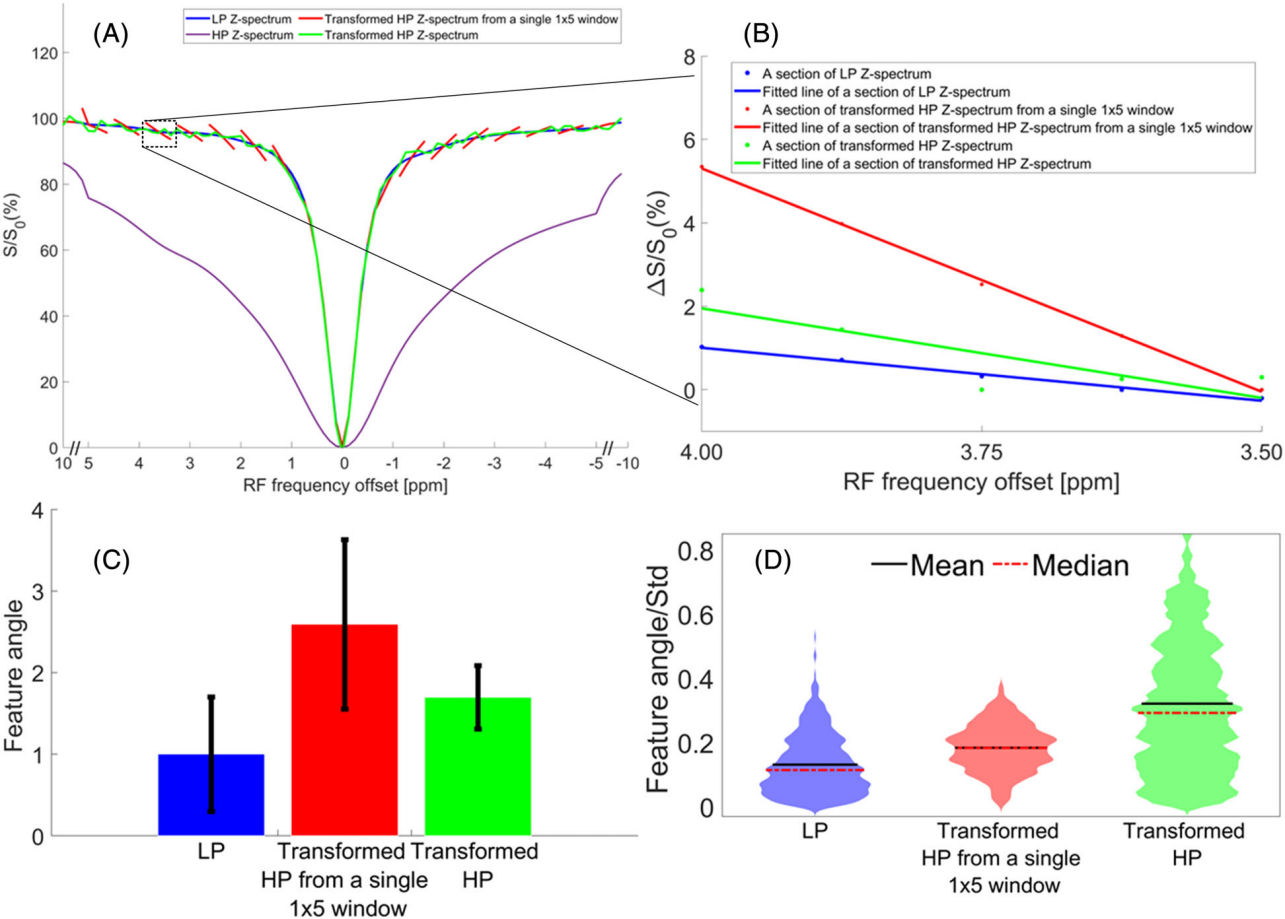
(A) An example pair of clean LP Z‐spectrum and clean HP Z‐spectrum from the digital phantom, along with the corresponding transformed HP Z‐spectrum from a single 1 × 5 Z‐spectral window and the transformed HP Z‐spectrum. (B) A section (from 3.5 ppm to 4.0 ppm) of these clean Z‐spectra in (A), with fitted lines. (C) Monte Carlo simulation of the feature angle from the LP Z‐spectrum, the HP Z‐spectrum from the 1 × 5 spectral window, and the transformed HP Z‐spectrum. Due to the different units on the *x*‐axis and *y*‐axis, a real angle degree cannot be calculated. Therefore, we define the feature angle from the LP Z‐spectrum as 1 unit, and express other feature angles as multiples of this unit. (D) Violin plot showing the ratio of all feature angles to their standard deviation for the LP Z‐spectrum, the transformed HP Z‐spectrum from a single 1 × 5 Z‐spectral window, and the transformed HP Z‐spectrum from the Monte Carlo simulation.

Figure 2 illustrates the principle of dual‐power feature preparation strategy for reducing the sensitivity of input Z‐spectrum to noise through a multiple‐pool model simulation. Figure 2A presents an example pair of clean LP Z‐spectrum and clean HP Z‐spectrum from the digital phantom, as well as the corresponding transformed HP Z‐spectrum from a single 1 × 5 Z‐spectral window and the transformed HP Z‐spectrum. Figure 2B displays a section (from 3.5 to 4.0 ppm) of these clean Z‐spectra shown in Figure 2A, with fitted lines forming an angle relative to the *x*‐axis. This angle serves as a rough measure of the average steepness of the slopes within a given frequency range, offering a straightforward method for comparing slopes. Therefore, it was termed “feature angle.” A Monte Carlo simulation was performed by adding 3% Gaussian noise to the clean Z‐spectra for 1000 times, and this feature angle was calculated. Figure 2C shows the mean and standard deviation of the feature angle for the LP Z‐spectra, transformed HP Z‐spectra from the 1 × 5 Z‐spectral window, and transformed HP Z‐spectra. The higher mean feature angle from the transformed HP Z‐spectrum using the 1 × 5 spectral window compared to the LP Z‐spectra is attributed to the zig–zag pattern. The lower mean feature angle from the transformed HP Z‐spectrum compared to that from the transformed HP Z‐spectrum using the 1 × 5 spectral window indicates the effective reduction of the zig‐zag pattern. Figure 2D shows a violin plot of the ratio of all feature angles to their standard deviation. The high mean ratio of the feature angles to their standard deviation in the transformed HP Z‐spectra compared to that from the LP Z‐spectra indicates the reduced sensitivity of the transformed HP Z‐spectra to noise.

### 3.2 Ablation Study and Optimization

**FIGURE 3:**
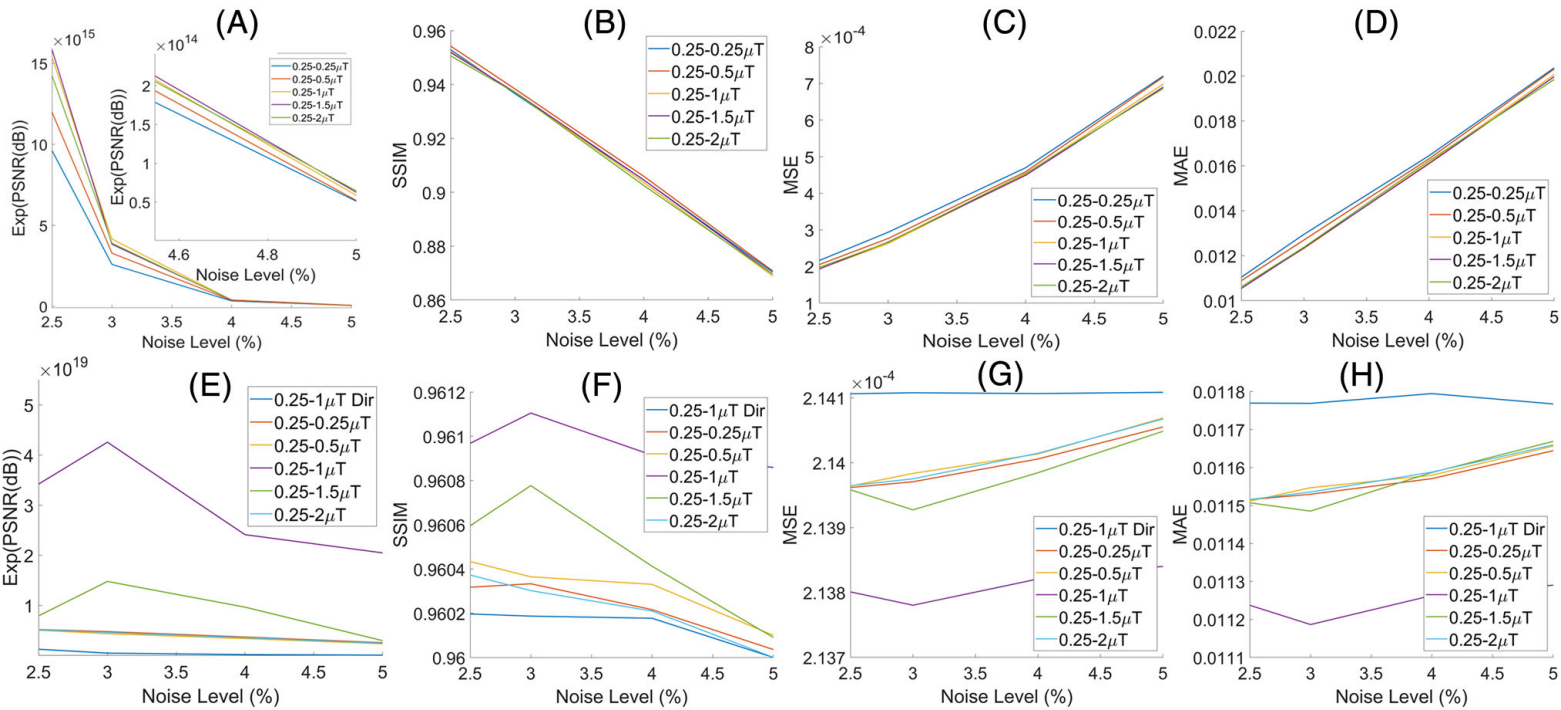
Exp(PSNR), SSIM, MSE, and MAE values for the average of the LP Z‐spectra and the transformed HP Z‐spectra with various high saturation powers (no DL prediction) (A–D), as well as for the DPDL predicted Z‐spectra using these Z‐spectra as inputs (E–H), for a few noise levels. The evaluation metric values for the DL predicted Z‐spectra using LP (0.25 μT) and HP (1 μT) Z‐spectra directly as inputs (no feature preparation) are also presented in (E–H), denoted as “0.25–1 μT dir.” The Exp(PSNR), SSIM, MSE, and MAE values are averaged across all voxels in the digital phantom.

Figure 3A–D illustrates the mean Exp(PSNR), SSIM, MSE, and MAE values from all samples in the digital phantom for the average of the LP Z‐spectra and the transformed HP Z‐spectra with various HPs (no DL prediction). Notably, the evaluation metric values across different HPs are quite close, which can be attributed to the presence of the zig‐zag pattern that affects the evaluation metric values to the same extent. Figure 3E–H presents these evaluation metric values for the DPDL predicted Z‐spectra using the same Z‐spectra in Figure 3A–D as input. It is evident that the Exp(PSNR) and SSIM values in Figure 3A–D are much lower than the corresponding values in Figure 3E–H. Conversely, the MSE and MAE values in Figure 3A–D are notably higher than the corresponding values in Figure 3E–H. This comparison underscores the critical role of the autoencoder network in reducing the zig–zag pattern and noise. Additionally, the Exp(PSNR) and SSIM values for the combination of two LP values of 0.25 μT are much lower than those for the combination of a LP of 0.25 μT and a HP of 1 μT in Figure 3E–H. Conversely, the MSE and MAE values for the two LP values of 0.25 μT are much higher than those with the LP of 0.25 μT and HP of 1 μT in Figure 3E–H. This demonstrates the significant contribution of the dual‐power feature preparation strategy to DPDL. Moreover, the Exp(PSNR) and SSIM values for the direct input of the LP and HP Z‐spectra to the autoencoder (no feature preparation) are lower than those of the corresponding DPDL prediction. Conversely, the MSE and MAE values for the direct input of the LP and HP Z‐spectra to the autoencoder are higher than those of the corresponding DPDL prediction. This underscores the importance of the feature preparation. Finally, the Exp(PSNR) and SSIM values using the LP of 0.25 μT and HP of 1 μT are higher than those for all other combinations of low and high powers in Figure 3E–H. Conversely, the MSE and MAE values for the low power of 0.25 μT and high power of 1 μT are lower than those for all other combinations of low and high powers in Figure 3E–H. This suggests that the combination of 0.25 μT and 1 μT is the optimal set of powers.

### 3.3 Simulation Validation

**FIGURE 4:**
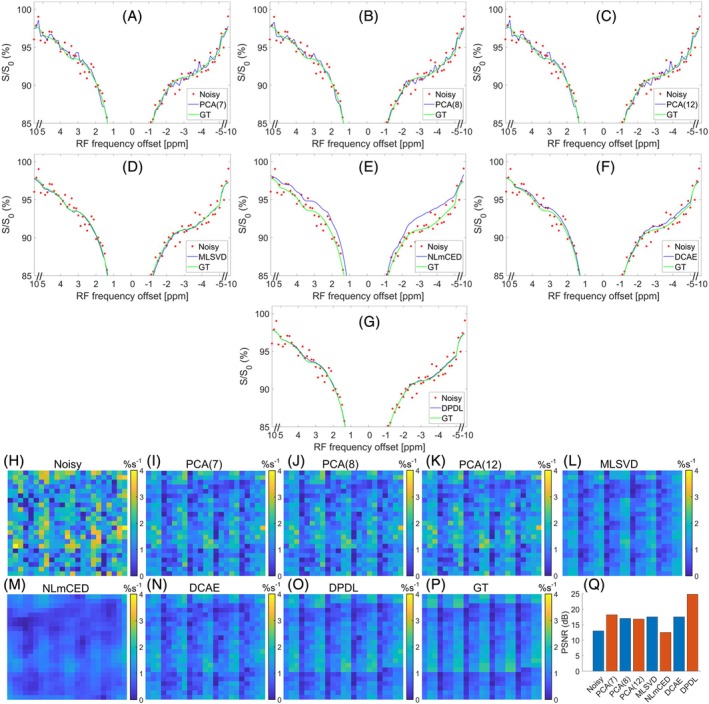
(A–G) display a sample LP Z‐spectrum from a single voxel in the noisy digital phantom, denoised using various methods: PCA(7) (A), PCA(8) (B), PCA(12) (C), MLSVD (D), NLmCED (E), DCAE (F), and DPDL (G). The noisy and GT LP Z‐spectrum are also included in these figures for comparison. The MSE values between the GT and the denoised Z‐spectra are 0.00038 (PCA(7)), 0.00041 (PCA(8)), 0.00047 (PCA(12)), 0.00048 (MLSVD), 0.00053 (NLmCED), 0.00044 (DCAE), and 0.00026 (DPDL), respectively. (H–P) display the LD‐fitted APT maps from the noisy, various denoised, and GT digital phantoms. (Q) shows the PSNR values between the LD‐fitted APT map from the GT digital phantom and that from the noisy and various denoised digital phantoms, which are 12.580 dB (noisy), 18.133 dB (PCA(7)), 17.638 dB (PCA(8)), 17.473 dB (PCA(12)), 17.600 dB (MLSVD), 11.214 dB (NLmCED), 17.706 dB (DCAE), and 24.271 dB (DPDL), respectively.

Figure 4A–G present the LP Z‐spectrum from a single sample in the digital phantom to demonstrate the DPDL's effectiveness in denoising the LP Z‐spectrum compared to other existing denoising techniques. The figures include the noisy LP Z‐spectrum and corresponding GT, along with the denoised counterparts obtained using various denoising methods. DPDL has the lowest MSE value between the GT and denoised Z‐spectrum, demonstrating its superior performance. While NLmCED shows smooth Z‐spectrum, it has significant bias from the GT. Figure S5 shows the corresponding LD fitted spectra. Similarly, DPDL has the lowest MSE value between the LD spectrum fitted from the GT Z‐spectrum and that from the denoised Z‐spectrum. Figure 4H–P compare the LD‐fitted APT maps from the digital phantom denoised by various methods. It is evident that PCA cannot effectively reduce the noise, and NLmCED produces significantly blurred image. This is further verified in Figure S6, which presents the residual maps between the GT and the denoised counterparts. Figure 4Q compares the PSNR values between the LD‐fitted APT map from the GT digital phantom and that from the noisy and various denoised digital phantoms. The PSNR value for DPDL is higher than that for other methods, highlighting its superior performance. The LD‐fitted guanidine, NOE(−1.6), and NOE(−3.5) maps from the digital phantom denoised by various methods, as well as the corresponding residual maps, are illustrated in Figures S7–S12.

Figure S13 compares the denoising performance of various methods on the entire digital phantom from two perspectives. Specifically, Figure S13A,C shows the SSIM between the denoised Z‐spectrum and the corresponding GT from each voxel in the digital phantom. Figure S13B,D shows the SSIM between the denoised 23 × 23 matrix in the digital phantom and the corresponding GT at each frequency offset. Notably, DPDL demonstrates significantly better denoising performance than other methods across the entire digital phantom.

### 3.4 Phantom Validation

**FIGURE 5:**
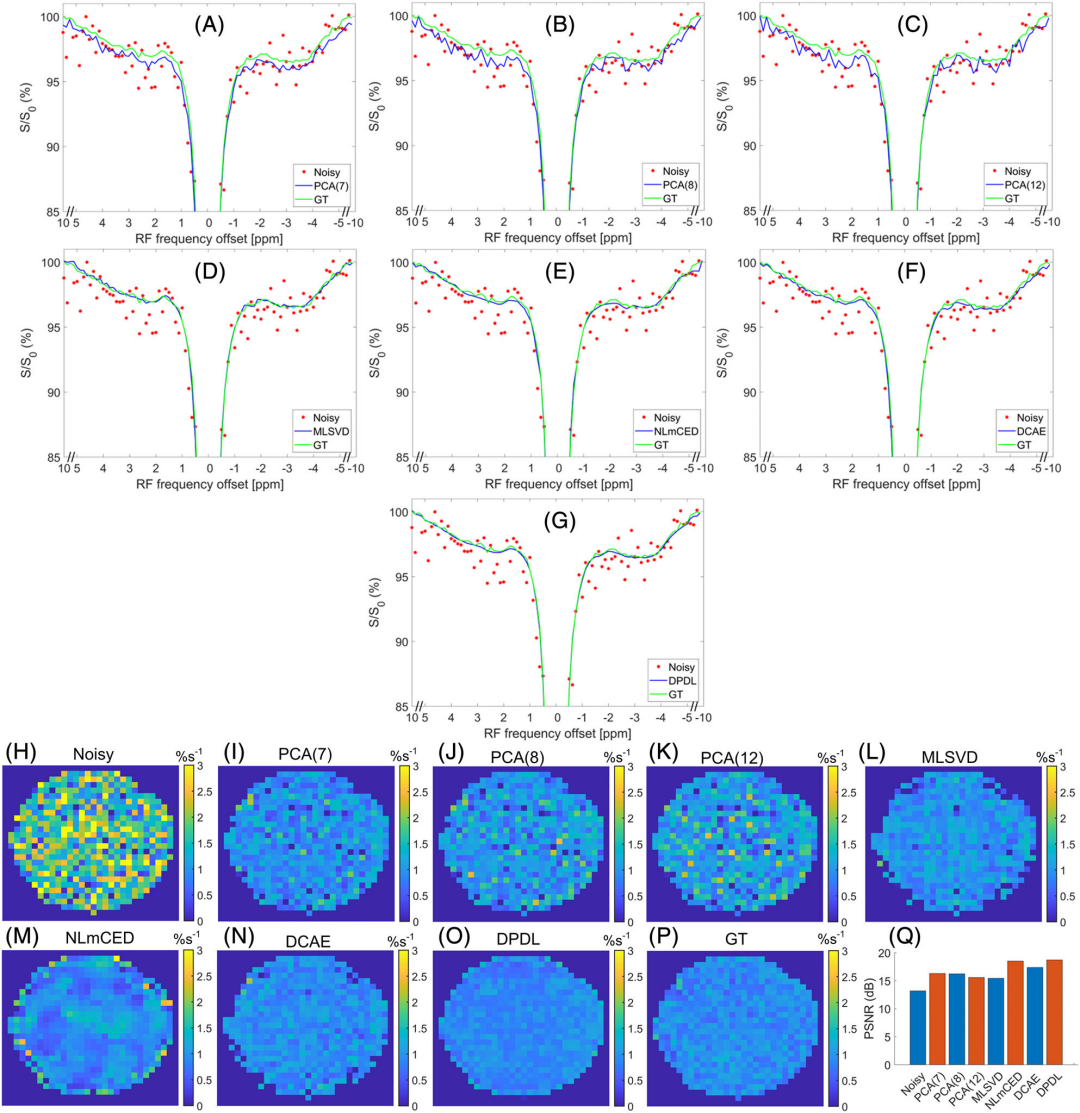
(A–G) display a sample LP Z‐spectrum from a single voxel in the noisy BSA phantom, denoised using various methods: PCA(7) (A), PCA(8) (B), PCA(12) (C), MLSVD (D), NLmCED (E), DCAE (F), and DPDL (G). The noisy and GT LP Z‐spectrum are also included in these figures for comparison. The MSE values between the GT and the denoised Z‐spectra are 0.000395 (PCA(7)), 0.000400 (PCA(8)), 0.000382 (PCA(12)), 0.000361 (MLSVD), 0.000385 (NLmCED), 0.000391 (DCAE), and 0.000250 (DPDL), respectively. (H–P) display the LD‐fitted APT maps from the noisy, denoised, and GT BSA phantoms. (Q) shows the PSNR values between the LD‐fitted APT map from the GT BSA phantom and that from the noisy and various denoised BSA phantoms, which are 13.196 dB (noisy), 16.282 dB (PCA(7)), 16.221 dB (PCA(8)), 15.571 dB (PCA(12)), 15.440 dB (MLSVD), 18.704 dB (NLmCED), 17.368 dB (DCAE), and 19.109 dB (DPDL), respectively.

Figure 5A–G present the noisy, denoised, and GT LP Z‐spectrum from a single voxel in the BSA phantom. The results show that DPDL outperforms other methods, achieving the lowest MSE values between the GT and denoised Z‐spectra. Figure S14 presents the corresponding LD fitted spectra. Similarly, DPDL shows the lowest MSE values between the LD spectrum fitted from the GT Z‐spectrum and that from the denoised Z‐spectrum. Figure 5H–P compares the LD‐fitted APT maps from the BSA phantom denoised by various methods. It is evident that PCA fails to effectively reduce noise, while NLmCED produces significantly blurred images. In contrast, DPDL achieves superior performance, as confirmed by the PSNR plot in Figure 5Q. Although NLmCED achieves a PSNR comparable to that of DPDL, it introduces additional contrast not present in the GT map, indicating poor denoising performance that led to inaccurate quantification. The observations on the BSA phantom are consistent with those observed in the digital phantom, with one exception: the PSNR for NLmCED is relatively higher in the BSA phantom compared to the digital phantom when compared to other methods. This highlights the sensitivity of NLmCED to structural variations, which are less prominent in the homogeneous BSA phantom.

### 3.5 Animal Experiments

Figure 6A–G present LP Z‐spectra from a single voxel located either in the tumor or normal tissue within a rat brain (#1). The figures include both the noisy LP Z‐spectrum and denoised counterparts obtained using various denoising methods. Both NLmCED and DPDL produce relatively smoother Z‐spectra. However, NLmCED introduces significant bias from the noisy data compared to the other methods. Figure S15 shows the corresponding LD fitted spectra. Figure 7 illustrates the LD‐fitted APT maps without denoising and with denoising by various methods, as well as the T_1_ map, from this rat. Figures S16–S20 show the LD‐fitted APT maps from other rats (#2–#6). Figures S21–S38 show the LD‐fitted maps of guanidine, NOE(−1.6), and NOE(−3.5) from all rats (#1–#6). Notably, both MLSVD and NLmCED exhibit patches of uniform intensity in most figures. In contrast, DPDL demonstrates superior denoising performance compared to other methods in most figures.

**FIGURE 6:**
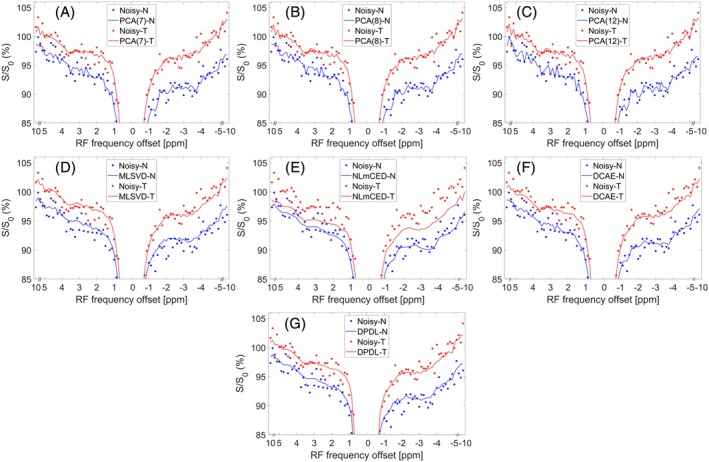
(A–G) display a sample LP Z‐spectrum from a single voxel in a tumor (T) and a single voxel in contralateral normal tissue (N) in a representative rat brain (#1), denoised using various methods: PCA(7) (A), PCA(8) (B), PCA(12) (C), MLSVD (D), NLmCED (E), DCAE (F), and DPDL (G). The noisy LP Z‐spectrum is also included in these figures for comparison.

**FIGURE 7:**
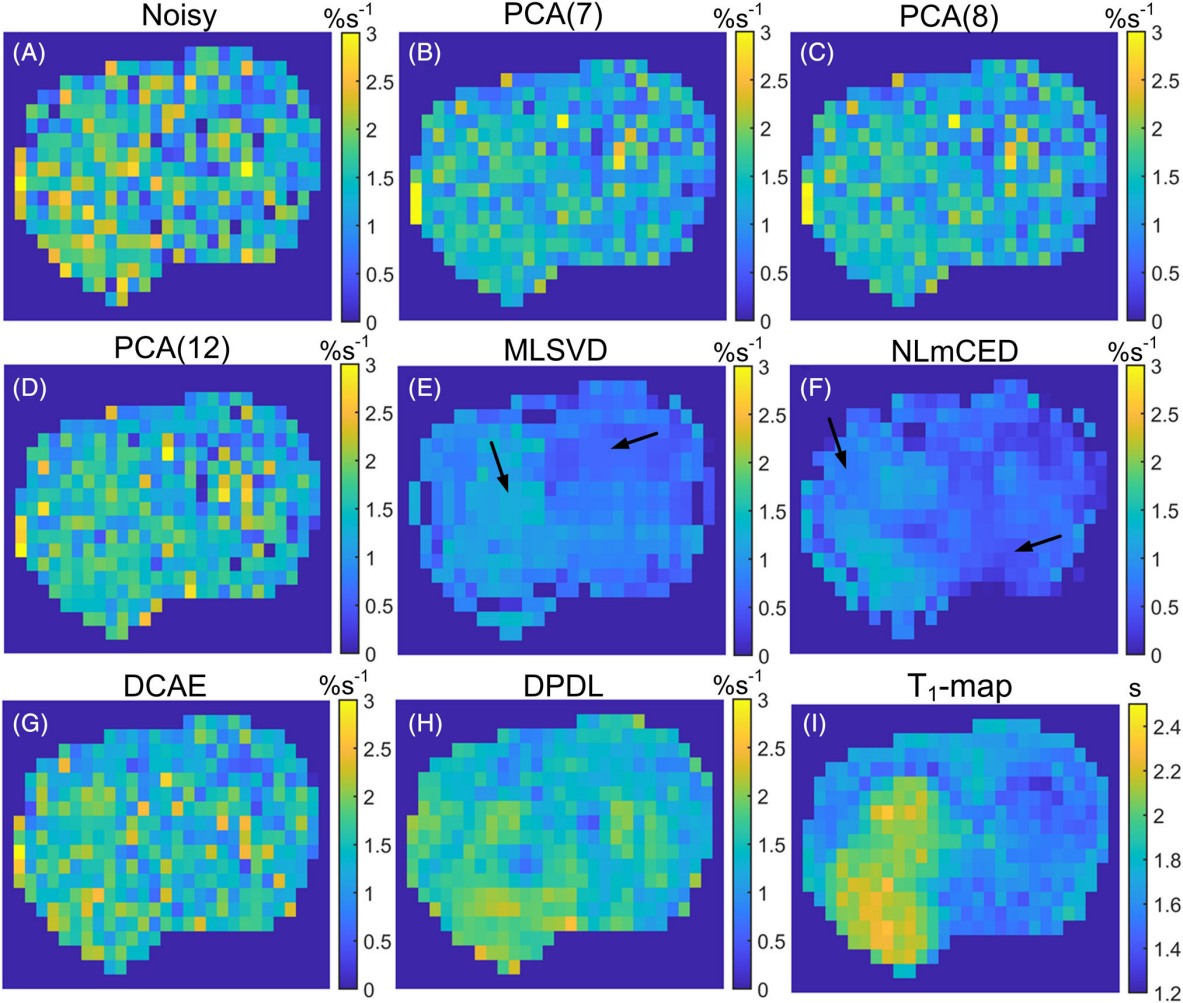
LD‐fitted APT maps from a representative rat brain bearing 9 L tumor (#1), without denoising (A) and with denoising by PCA(7) (B), PCA(8) (C), PCA(12) (D), MLSVD (E), NLmCED (F), DCAE (G), and DPDL (H). T_1_ map was shown in (I) to demonstrate the tumor region. Arrows in (E) and (F) point to patches of uniform intensity, highlighting the suboptimal performance of the denoising.

Figure 8A–G presents LP Z‐spectra from a single voxel in muscle tissue within a rat leg (#1). Similarly, NLmCED shows significant bias from noisy data compared to others. In contrast, DPDL demonstrates a relatively smooth Z‐spectrum with minimal bias. Figure S39 shows the corresponding LD fitted spectra. Figure 9 illustrates the LD‐fitted APT maps without denoising and with denoising by various methods from this rat. Figures S40–S44 show the LD‐fitted APT maps from other rats (#2–#6). Figures S45–S62 show the LD‐fitted maps of PCr, guanidine, and NOE(−3.5) from all rats (#1–#6). DPDL demonstrates superior denoising performance compared to other methods in most figures.

**FIGURE 8:**
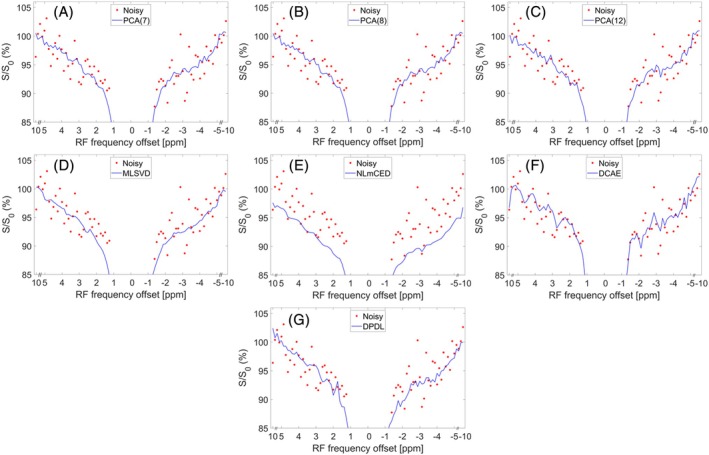
(A–G) display a sample LP Z‐spectrum from a single voxel in muscle in a representative healthy rat leg (#1), denoised using various methods: PCA(7) (A), PCA(8) (B), PCA(12) (C), MLSVD (D), NLmCED (E), DCAE (F), and DPDL (G). The noisy LP Z‐spectrum is also included in these figures for comparison.

**FIGURE 9:**
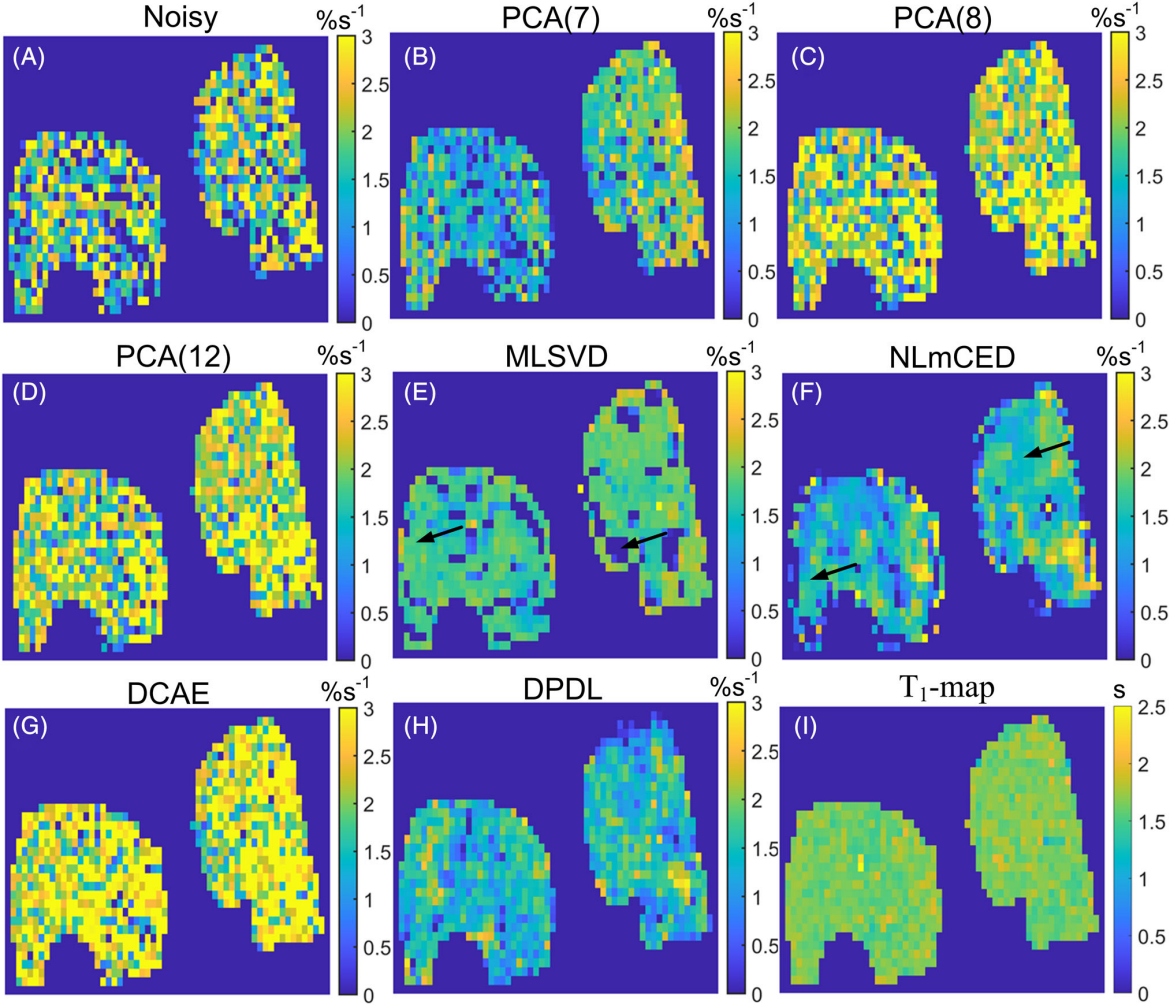
LD‐fitted APT maps from a representative rat leg (#1), without denoising (A) and with denoising by PCA(7) (B), PCA(8) (C), PCA(12) (D), MLSVD (E), NLmCED (F), DCAE (G), and DPDL (H). T_1_ map was also shown in (I). Arrows in (E) and (F) point to patches of uniform intensity, highlighting the suboptimal performance of the denoising.

**FIGURE 10:**
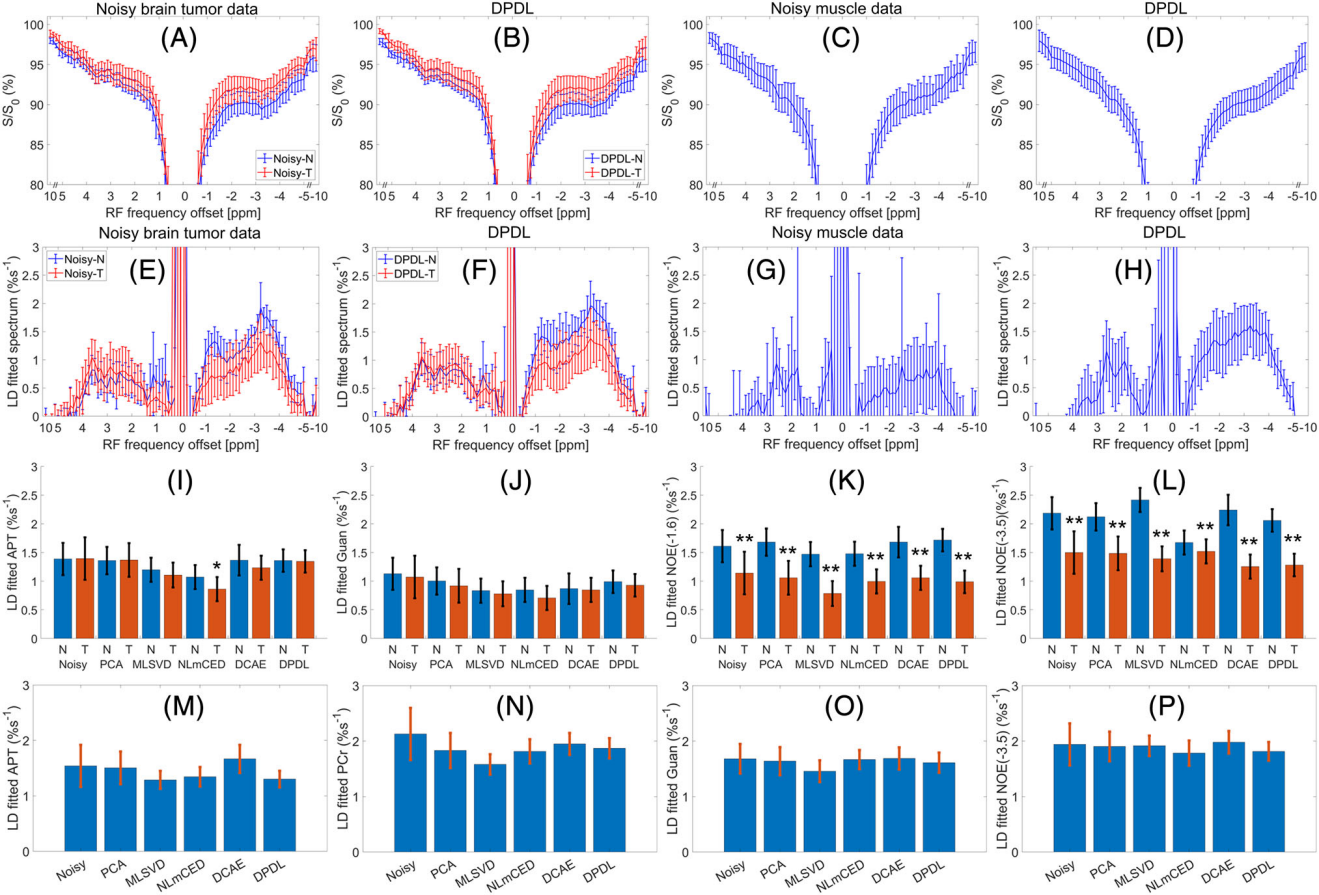
Mean and standard deviation of the LP Z‐spectra and corresponding LD‐fitted spectra from tumors (T) and contralateral normal tissues (N) in rat brains (A, B, E, F), as well as from muscle tissues in rat legs (C, D, G, H), from the noisy data (A, C, E, G) and the denoised counterparts by the DPDL method (B, D, F, H). Statistical analysis of the LD‐fitted APT (I), guanidine (J), NOE(−1.6) (K), and NOE(−3.5) (L) in the tumors and contralateral normal tissues in rat brains, as well as the LD‐fitted APT (M), PCr (N), guanidine (O), and NOE(−3.5) (P) in leg muscles, denoised by various methods. The Z‐spectrum for each ROI was first calculated by averaging the Z‐spectra of all voxels within the ROI for each animal. Subsequently, the mean and standard deviation across all animals were derived. Statistical differences between tumors and normal tissues were assessed using a paired Student's t‐test, with significance indicated as *p < 0.05 and **p < 0.02.

Figure 10A–H show the mean and standard deviation of the LP Z‐spectra and corresponding LD‐fitted spectra from the tumors and normal tissues in all rat brains (#1–#6) and all rat leg muscles (#1–#6). The APT, guanidine, NOE(−1.6), and NOE(−3.5) peaks are clearly observed on the LD‐fitted spectra after the DPDL denoising from brain tissues. Additionally, the APT, PCr, guanidine, and NOE(−3.5) peaks are distinctly visible on the LD‐fitted spectra after the DPDL denoising from muscle tissues. Figure 10I–P show the statistical analysis of the LD‐fitted APT, guanidine, NOE(−1.6), and NOE(−3.5) amplitudes in the tumors and normal tissues in brains, as well as the LD‐fitted APT, PCr, guanidine, and NOE(−3.5) amplitudes in leg muscles, denoised by various methods. For all denoising methods, significant differences between tumors and normal brain tissues are evident in the NOE(−1.6) and NOE(−3.5) images, whereas APT (except NLmCED) and guanidine CEST images do not show such distinctions.

## 4 Discussion

We introduced a novel CEST denoising method that leverages a DL approach along with a dual‐power feature preparation strategy, tailored for LP applications. Feature preparation is essential in DL as it optimizes, normalizes, and scales the input data to ensure efficient training [41, 42]. By using appropriate high powers, the slopes of the HP Z‐spectrum within certain frequency offset ranges are steeper than those of the LP Z‐spectrum, making them less susceptible to noise. These features can be captured by our designed Z‐spectral window and shifted to the LP Z‐spectral data range, resulting in a transformed HP Z‐spectrum. This transformed HP Z‐spectrum enhances the DL model's ability to capture and reconstruct the underlying structure of the LP Z‐spectra with extremely low SNR or CNR. However, this Z‐spectral window causes a zig‐zag pattern in the transformed HP Z‐spectrum, introducing bias. Unlike noise, this pattern is not random; it is related to the fine structure of the Z‐spectrum and can be predicted and absorbed by the DL model. Thus, both the dual‐power feature preparation strategy and the DL model play crucial roles in the DPDL denoising. In this study, the HP of 1 μT is used based on the optimization study. It is important to note that at much higher powers, the CEST effects become significantly broader, diminishing the slope and, consequently, reducing data features.

In this study, APT and guanidine CEST imaging did not show significant differences between tumors and normal tissues, while NOE(−1.6) and NOE(−3.5) did. The lack of significant difference in APT is consistent with previous studies using AREX for CEST quantification [43, 44], but not conventional APT‐weighted imaging [9]. The mechanism behind this discrepancy remains unclear. The absence of significant differences in guanidine CEST contrasts with previous studies [45, 46], which might be due to the lower power used in this study compared to earlier research. The LP may result in relatively higher contribution from protein arginine, which has a slower exchange rate, and less from creatine, which has a faster exchange rate [14, 47, 48, 49, 50, 51, 52]. The significant decreases in NOE(−1.6) and NOE(−3.5) in tumors are consistent with our previous report [53], possibly attributed to reduced levels of macromolecules [19, 20], in tumors. The consistency of APT and NOE imaging with previous studies conducted at higher powers suggests that these contrasts are not influenced by saturation power. This is likely because their exchange/coupling rates are close in both tumors and normal tissues, thereby providing a consistent trend and degree of variation across different powers.

In the LD spectra in Figure 10, the NOE(−1.6) in brains, which is difficult to observe at high powers and low fields due to significant DS effect [54], becomes distinguishable at LP. Additionally, the small peak at 2.6 ppm in brains [15], which is often obscured by the overlapping APT and guanidine CEST effects at high powers and low fields, is distinguishable at LP. Furthermore, the significant NOE effects from −1 to −2 ppm in muscles, although not showing a clear peak, indicate the presence of NOE within this frequency range that may include the glycogen NOE [55, 56] and other effects. The lack of a clear peak may be due to the use of LD fitting, which uses the CEST signal from 0 to ±0.5 ppm as a reference signal, thereby underestimating nearby effects. Developing advanced CEST quantification methods for the low‐power applications is warranted to further enhance its accuracy, and our denoising method can enhance the performance of any CEST quantification method.

The dual‐power feature preparation in DPDL may be sensitive to B_1_ inhomogeneity. Nonetheless, in preclinical MRI, the B_1_ shifts are minimal, and our experiments using digital phantoms with B_1_ shifts of up to 15% demonstrate encouraging results. In clinical 3T, where B_1_ inhomogeneity may be more pronounced, measuring and compensating for B_1_ shifts would be necessary. Additionally, the computational cost of our DPDL is significantly higher than that of conventional methods. Table S4 provides a comparison of the time and space complexities of various methods using Big O notation. Despite the increased computational demand, running DPDL remains feasible on modern computers. For instance, on our systems, the training time is about 42 h, and the testing time for a single rat is approximately 2 mins. Moreover, a lipid suppression technique was applied to muscle to reduce lipid signals. Whether or not the lipid signal is fully suppressed, it does not affect the denoising performance of DPDL. This is attributed to the model being trained on a general four‐pool simulated dataset and fine‐tuned on tissue‐specific Z‐spectra, making it independent of any alterations in the Z‐spectra caused by lipid suppression. However, if the lipid signal is not fully suppressed, it can influence the LD‐fitted NOE(−3.5), as residual lipid signals may overlap with NOE(−3.5). Furthermore, it should be noted that the DL denoising model is protocol‐specific and requires retraining if the imaging parameters are significantly altered. This contrasts with the conventional denoising methods. Future work will focus on improving the generalizability of DPDL to accommodate diverse imaging protocols.

## 5 Conclusion

The proposed DPDL significantly enhances the SNR of CEST Z‐spectrum acquired with low powers, enabling the exploration of new pools and improving the observation and quantification of CEST effects, particularly at low fields. Validation on digital and BSA phantoms, and animal experiments demonstrated superior denoising performance and improved image quality compared to existing methods. However, the DPDL method is protocol‐specific and may require retraining for different datasets, unlike conventional methods that are universally applicable. Despite this limitation, this advancement holds promise for enhancing the utility of CEST imaging in both research and clinical applications.

## Supporting information


**Figure S1:** (A) A pair of clean LP Z‐spectrum and clean HP Z‐spectrum from a modified two‐pool (amide and water) model simulation. (B) The clean LP Z‐spectrum and the corresponding transformed HP Z‐spectrum from a single 1 × 5 Z‐spectral window. (C) The corresponding transformed HP Z‐spectrum from a single 1 × 5 Z‐spectral window and that from a single 1 × 3 Z‐spectral window. (D) The corresponding transformed HP Z‐spectrum from a single 1 × 5 Z‐spectral window and that from a single shifted 1 × 5 Z‐spectral window. (E) The corresponding transformed HP Z‐spectrum from a single 1 × 3 Z‐spectral window and that from a single shifted 1 × 3 Z‐spectral window. (F) The corresponding transformed HP Z‐spectrum averaged from both the 1 × 5 and 1 × 3 Z‐spectral windows, including their shifted Z‐spectral windows, which is simply referred to as the “transformed HP Z‐spectrum”, unless otherwise specified. (G) A section (from 3.5 ppm to 4.0 ppm) of the clean LP Z‐spectrum, the corresponding transformed HP Z‐spectrum from a single 1 × 5 Z‐spectral window, and corresponding transformed HP Z‐spectrum, with fitted lines. (H) Monte Carlo simulation of the feature angle from the Z‐spectra in (G). The feature angle is determined by fitting CEST signals within this frequency range to a line and then calculating the angle between this fitted line and the *x*‐axis. This feature angle can roughly reflect the mean steepness of the Z‐spectrum within this frequency range, offering a straightforward method for comparing slopes of the Z‐spectrum. Due to the different units on the *x*‐axis and *y*‐axis, a real angle degree cannot be calculated. Therefore, we define the feature angle from the LP Z‐spectrum as 1 unit, and express other feature angles as multiples of this unit. (I) Violin plot showing the ratio of all feature angles to their standard deviation for the Z‐spectra in (G) from the Monte Carlo simulation. The sample parameters for this modified two‐pool model simulation are based on Table S2, with the following modifications to highlight the APT peak: (1) a significantly higher *f*_s_ of 1% was simulated to emphasize the APT peak; (2) the DS effect was removed by nulling ω_1_ applied on water in Bloch equations. Additionally, *k*_sw_ was set to 50s^−1^, and *T*_2s_ was set to 2 ms. The Monte Carlo simulation was performed by adding 3% Gaussian noise to the clean Z‐spectra for 1000 times, and the feature angle was calculated.
**Figure S2:** Mean measured CEST Z‐spectra from brains of six rats bearing 9 L tumor (A) and leg muscle tissues of six healthy rats (B) at 4.7 T, using saturation fields of 0.25, 0.5, and 1.0 μT, respectively.
**Figure S3:** Comparative analysis of PSNR values of the DPDL‐predicted Z‐spectra from the digital phantom, for various combinations of Z‐spectral window length across different noise levels. The PSNR values are averaged across all voxels in the digital phantom.
**Figure S4:** Measured CEST signals at 3.5 ppm from the whole brain of a rat over 100 repetitions with saturation field strengths of 0.25 and 1 μT, and at 4.7T. To calculate the standard deviation (std), the first 10 repetitions were excluded to avoid transient effects. The std. values are roughly similar for both saturation field strengths, suggesting that the CEST signals for these two saturation field strengths have comparable noise levels.
**Figure S5:** LD‐fitted spectrum (A–G) from a single voxel in the noisy digital phantom, denoised using various methods: PCA(7) (A), PCA(8) (B), PCA(12) (C), MLSVD (D), NLmCED (E), DCAE (F), and DPDL (G). In (A–G), the MSE values between the LD spectra fitted from the GT Z‐spectra and those fitted from the denoised Z‐spectra are 0.00046, 0.00054, 0.00062, 0.00043, 0.00085, 0.00061, and 0.00023, respectively.
**Figure S6:** Residual maps showing the differences between the APT map from the GT digital phantom and that from various sources: the noisy digital phantom (A), the noisy digital phantom denoised by PCA(7) (B), PCA(8) (C), PCA(12) (D), MLSVD (E), NLmCED (F), DCAE (G), and DPDL (H). The mean absolute values of the residual maps are 0.7774, 0.3266, 0.3274, 0.3383, 0.2484, 0.4437, 0.2350, and 0.1189 for noisy, PCA, MLSVD, NLmCED, DCAE, and DPDL, respectively.
**Figure S7:** (A–I) LD‐fitted guanidine maps from the noisy digital phantom, the noisy digital phantom denoised by PCA(7), PCA(8), PCA(12), MLSVD, NLmCED, DCAE, and DPDL methods, as well as the GT digital phantom. (J) PSNR values between the LD‐fitted guanidine map from the GT digital phantom and that from the noisy digital phantom [12.66 dB], as well as those denoised by PCA(7) [18.24 dB], PCA(8) [17.49 dB], PCA(12) [17.34 dB], MLSVD [18.045 dB], NLmCED [12.715 dB], DCAE [18.425 dB], and DPDL [24.22 dB] methods.
**Figure S8:** Residual maps showing the differences between the guanidine map from the GT digital phantom and that from various sources: the noisy digital phantom (A), the noisy digital phantom denoised by PCA(7) (B), PCA(8) (C), PCA(12) (D), MLSVD (E), NLmCED (F), DCAE (G), and DPDL (H). The mean absolute values of the residual maps are 0.732, 0.328, 0.334, 0.338, 0.261, 0.444, 0.225, and 0.116 for noisy, PCA (7), PCA (8), PCA (12), MLSVD, NLmCED, DCAE, and DPDL, respectively.
**Figure S9:** (A–I) LD‐fitted NOE(−1.6) maps from the noisy digital phantom, the noisy digital phantom denoised by PCA(7), PCA(8), PCA(12), MLSVD, NLmCED, DCAE, and DPDL methods, as well as the GT digital phantom. (J) PSNR values between the LD‐fitted NOE(−1.6) map from the GT digital phantom and that from the noisy digital phantom [13.71 dB], as well as those denoised by PCA(7) [18.62 dB], PCA(8) [17.83 dB], PCA(12) [17.54 dB], MLSVD [19.01 dB], NLmCED [14.145 dB], DCAE [19.51 dB], and DPDL [24.51 dB] methods.
**Figure S10:** Residual maps showing the differences between the NOE(−1.6) map from the GT digital phantom and that from various sources: the noisy digital phantom (A), the noisy digital phantom denoised by PCA(7) (B), PCA(8) (C), PCA(12) (D), MLSVD (E), NLmCED (F), DCAE (G), and DPDL (H). The mean absolute values of the residual maps are 0.772, 0.309, 0.327, 0.348, 0.228, 0.438, 0.226, and 0.129 for noisy, PCA(7), PCA(8), PCA(12), MLSVD, NLmCED, DCAE, and DPDL, respectively.
**Figure S11:** (A–I) LD‐fitted NOE(−3.5) maps from the noisy digital phantom, the noisy digital phantom denoised by PCA(7), PCA(8), PCA(12), MLSVD, NLmCED, DCAE, and DPDL methods, as well as the GT digital phantom. (J) PSNR values between the LD‐fitted NOE(−3.5) map from the GT digital phantom and that from the noisy digital phantom [13.46 dB], as well as those denoised by PCA(7) [18.56 dB], PCA(8) [18.24 dB], PCA(12) [17.92 dB], MLSVD [19.705 dB], NLmCED [16.58 dB], DCAE [20.78 dB], and DPDL [26.84 dB] methods.
**Figure S12:** Residual maps showing the differences between the NOE(−3.5) map from the GT digital phantom and that from various sources: the noisy digital phantom (A), the noisy digital phantom denoised by PCA(7) (B), PCA(8) (C), PCA(12) (D), MLSVD (E), NLmCED (F), DCAE (G), and DPDL (H). The mean absolute values of the residual maps are 0.692, 0.274, 0.286, 0.293, 0.256, 0.429, 0.275, and 0.112 for noisy, PCA(7), PCA(8), PCA(12), MLSVD, NLmCED, DCAE, and DPDL, respectively.
**Figure S13:** (A) Comparison of the SSIM between the denoised Z‐spectrum and the corresponding GT from each voxel in the digital phantom among various denoising methods. (B) Comparison of the SSIM between the denoised 23 × 23 matrix in the digital phantom and the corresponding GT at each frequency offset among various denoising methods. (C) and (D) show vertically zoomed versions of (A) and (B), respectively, highlighting the performance of the MLSVD, NLmCED, DCAE, and DPDL methods in comparison to the clean data.
**Figure S14:** The sample LP LD‐fitted spectrum from a single voxel in the noisy BSA phantom, denoised using various methods: PCA(7) (A), PCA(8) (B), PCA(12) (C), MLSVD (D), NLmCED (E), DCAE (F), and DPDL (G). In (A–G), the MSE values between the LD spectra fitted from the GT Z‐spectra and those fitted from the denoised Z‐spectra are 0.000390, 0.000426, 0.000414, 0.000372, 0.0002885, 0.000386, and 0.000225, respectively. The noisy LP LD‐fitted spectrum and GT are also included in these figures for comparison.
**Figure S15:** The sample LP LD‐fitted spectrum from a single voxel in a tumor (T) and a single voxel in contralateral normal tissue (N) in a representative rat brain (#1), denoised using various methods: PCA(7) (A), PCA(8) (B), PCA(12) (C), MLSVD (D), NLmCED (E), DCAE (F), and DPDL (G). The noisy LP LD‐fitted spectrum and GT are also included in these figures for comparison.
**Figure S16:** LD‐fitted APT maps from a rat brain bearing a 9 L tumor (#2), without denoising (A) and with denoising by PCA(7) (B), PCA(8) (C), PCA(12) (D), MLSVD (E), NLmCED (F), DCAE (G), and DPDL (H). T_1_ map was shown in (I) to demonstrate the tumor region. Arrows in (E) and (F) point to patches of uniform intensity, highlighting the suboptimal performance of the denoising.
**Figure S17:** LD‐fitted APT maps from a rat brain bearing a 9 L tumor (#3), without denoising (A) and with denoising by PCA(7) (B), PCA(8) (C), PCA(12) (D), MLSVD (E), NLmCED (F), DCAE (G), and DPDL (H). T_1_ map was shown in (I) to demonstrate the tumor region. Arrows in (E) and (F) point to patches of uniform intensity, highlighting the suboptimal performance of the denoising.
**Figure S18:** LD‐fitted APT maps from a rat brain bearing a 9 L tumor (#4), without denoising (A) and with denoising by PCA(7) (B), PCA(8) (C), PCA(12) (D), MLSVD (E), NLmCED (F), DCAE (G), and DPDL (H). T_1_ map was shown in (I) to demonstrate the tumor region. Arrows in (E) and (F) point to patches of uniform intensity, highlighting the suboptimal performance of the denoising.
**Figure S19:** LD‐fitted APT maps from a rat brain bearing a 9 L tumor (#5), without denoising (A) and with denoising by PCA(7) (B), PCA(8) (C), PCA(12) (D), MLSVD (E), NLmCED (F), DCAE (G), and DPDL (H). T_1_ map was shown in (I) to demonstrate the tumor region.
**Figure S20:** LD‐fitted APT maps from a rat brain bearing a 9 L tumor (#6), without denoising (A) and with denoising by PCA(7) (B), PCA(8) (C), PCA(12) (D), MLSVD (E), NLmCED (F), DCAE (G), and DPDL (H). T_1_ map was shown in (I) to demonstrate the tumor region. Arrows in (F) point to patches of uniform intensity, highlighting the suboptimal performance of the denoising.
**Figure S21:** LD‐fitted Guanidine maps from a rat brain bearing a 9 L tumor (#1), without denoising (A) and with denoising by PCA(7) (B), PCA(8) (C), PCA(12) (D), MLSVD (E), NLmCED (F), DCAE (G), and DPDL (H). T_1_ map was shown in (I) to demonstrate the tumor region. Arrows in (E) and (F) point to patches of uniform intensity, highlighting the suboptimal performance of the denoising.
**Figure S22:** LD‐fitted Guanidine maps from a rat brain bearing a 9 L tumor (#2), without denoising (A) and with denoising by PCA(7) (B), PCA(8) (C), PCA(12) (D), MLSVD (E), NLmCED (F), DCAE (G), and DPDL (H). T_1_ map was shown in (I) to demonstrate the tumor region. Arrows in (E) and (F) point to patches of uniform intensity, highlighting the suboptimal performance of the denoising.
**Figure S23:** LD‐fitted Guanidine maps from a rat brain bearing a 9 L tumor (#3), without denoising (A) and with denoising by PCA(7) (B), PCA(8) (C), PCA(12) (D), MLSVD (E), NLmCED (F), DCAE (G), and DPDL (H). T_1_ map was shown in (I) to demonstrate the tumor region. Arrows in (E) and (F) point to patches of uniform intensity, highlighting the suboptimal performance of the denoising.
**Figure S24:** LD‐fitted Guanidine maps from a rat brain bearing a 9 L tumor (#4), without denoising (A) and with denoising by PCA(7) (B), PCA(8) (C), PCA 12) (D), MLSVD (E), NLmCED (F), DCAE (G), and DPDL (H). T_1_ map was shown in (I) to demonstrate the tumor region. Arrows in (E) and (F) point to patches of uniform intensity, highlighting the suboptimal performance of the denoising.
**Figure S25:** LD‐fitted Guanidine maps from a rat brain bearing a 9 L tumor (#5), without denoising (A) and with denoising by PCA(7) (B), PCA(8) (C), PCA(12) (D), MLSVD (E), NLmCED (F), DCAE (G), and DPDL (H). T_1_ map was shown in (I) to demonstrate the tumor region.
**Figure S26:** LD‐fitted Guanidine maps from a rat brain bearing a 9 L tumor (#6), without denoising (A) and with denoising by PCA(7) (B), PCA(8) (C), PCA(12) (D), MLSVD (E), NLmCED (F), DCAE (G), and DPDL (H). T_1_ map was shown in (I) to demonstrate the tumor region. Arrows in (E) and (F) point to patches of uniform intensity, highlighting the suboptimal performance of the denoising.
**Figure S27:** LD‐fitted NOE(−1.6) maps from a rat brain bearing a 9 L tumor (#1), without denoising (A) and with denoising by PCA(7) (B), PCA(8) (C), PCA(12) (D), MLSVD (E), NLmCED (F), DCAE (G), and DPDL (H). T_1_ map was shown in (I) to demonstrate the tumor region. Arrows in (E) and (F) point to patches of uniform intensity, highlighting the suboptimal performance of the denoising.
**Figure S28:** LD‐fitted NOE(−1.6) maps from a rat brain bearing a 9 L tumor (#2), without denoising (A) and with denoising by PCA(7) (B), PCA(8) (C), PCA(12) (D), MLSVD (E), NLmCED (F), DCAE (G), and DPDL (H). T_1_ map was shown in (I) to demonstrate the tumor region. Arrows in (E) and (F) point to patches of uniform intensity, highlighting the suboptimal performance of the denoising.
**Figure S29:** LD‐fitted NOE(−1.6) maps from a rat brain bearing a 9 L tumor (#3), without denoising (A) and with denoising by PCA(7) (B), PCA(8) (C), PCA(12) (D), MLSVD (E), NLmCED (F), DCAE (G), and DPDL (H). T_1_ map was shown in (I) to demonstrate the tumor region. Arrows in (E) and (F) point to patches of uniform intensity, highlighting the suboptimal performance of the denoising.
**Figure S30:** LD‐fitted NOE(−1.6) maps from a rat brain bearing a 9 L tumor (#4), without denoising (A) and with denoising by PCA(7) (B), PCA(8) (C), PCA(12) (D), MLSVD (E), NLmCED (F), DCAE (G), and DPDL (H). T_1_ map was shown in (I) to demonstrate the tumor region. Arrows in (E) and (F) point to patches of uniform intensity, highlighting the suboptimal performance of the denoising.
**Figure S31:** LD‐fitted NOE(−1.6) maps from a rat brain bearing a 9 L tumor (#5), without denoising (A) and with denoising by PCA(7) (B), PCA(8) (C), PCA(12) (D), MLSVD (E), NLmCED (F), DCAE (G), and DPDL (H). T_1_ map was shown in (I) to demonstrate the tumor region.
**Figure S32:** LD‐fitted NOE(−1.6) maps from a rat brain bearing a 9 L tumor (#6), without denoising (A) and with denoising by PCA(7) (B), PCA(8) (C), PCA(12) (D), MLSVD (E), NLmCED (F), DCAE (G), and DPDL (H). T_1_ map was shown in (I) to demonstrate the tumor region. Arrows in (F) point to patches of uniform intensity, highlighting the suboptimal performance of the denoising.
**Figure S33:** LD‐fitted NOE(−3.5) maps from a rat brain bearing a 9 L tumor (#1), without denoising (A) and with denoising by PCA(7) (B), PCA(8) (C), PCA(12) (D), MLSVD (E), NLmCED (F), DCAE (G), and DPDL (H). T_1_ map was shown in (I) to demonstrate the tumor region. Arrows in (E) and (F) point to patches of uniform intensity, highlighting the suboptimal performance of the denoising.
**Figure S34:** LD‐fitted NOE(−3.5) maps from a rat brain bearing a 9 L tumor (#2), without denoising (A) and with denoising by PCA(7) (B), PCA(8) (C), PCA(12) (D), MLSVD (E), NLmCED (F), DCAE (G), and DPDL (H). T_1_ map was shown in (I) to demonstrate the tumor region. Arrows in (E) and (F) point to patches of uniform intensity, highlighting the suboptimal performance of the denoising.
**Figure S35:** LD‐fitted NOE(−3.5) maps from a rat brain bearing a 9 L tumor (#3), without denoising (A) and with denoising by PCA(7) (B), PCA(8) (C), PCA(12) (D), MLSVD (E), NLmCED (F), DCAE (G), and DPDL (H). T_1_ map was shown in (I) to demonstrate the tumor region. Arrows in (E) and (F) point to patches of uniform intensity, highlighting the suboptimal performance of the denoising.
**Figure S36:** LD‐fitted NOE(−3.5) maps from a rat brain bearing a 9 L tumor (#4), without denoising (A) and with denoising by PCA(7) (B), PCA(8) (C), PCA(12) (D), MLSVD (E), NLmCED (F), DCAE (G), and DPDL (H). T_1_ map was shown in (I) to demonstrate the tumor region. Arrows in (E) and (F) point to patches of uniform intensity, highlighting the suboptimal performance of the denoising.
**Figure S37:** LD‐fitted NOE(−3.5) maps from a rat brain bearing a 9 L tumor (#5), without denoising (A) and with denoising by PCA (7) (B), PCA (8) (C), PCA (12) (D), MLSVD (E), NLmCED (F), DCAE (G), and DPDL (H). T_1_ map was shown in (I) to demonstrate the tumor region.
**Figure S38:** LD‐fitted NOE(−3.5) maps from a rat brain bearing a 9 L tumor (#6), without denoising (A) and with denoising by PCA(7) (B), PCA(8) (C), PCA(12) (D), MLSVD (E), NLmCED (F), DCAE (G), and DPDL (H). T_1_ map was shown in (I) to demonstrate the tumor region.
**Figure S39:** LD‐fitted spectrum from a single voxel in muscle in a representative healthy rat leg (#1), after denoising using various methods: PCA(7) (A), PCA(8) (B), PCA(12) (C), MLSVD (D), NLmCED (E), DCAE (F), and DPDL (G). The noisy LP LD‐fitted spectrum is also included in these figures for comparison.
**Figure S40:** LD‐fitted APT maps from the leg muscle in a rat (#2), without denoising (A) and with denoising by PCA(7) (B), PCA(8) (C), PCA(12) (D), MLSVD (E), NLmCED (F), DCAE (G), and DPDL (H). T_1_ map was shown in (I). Arrows in (E) point to patches of uniform intensity, highlighting the suboptimal performance of the denoising.
**Figure S41:** LD‐fitted APT maps from the leg muscle in a rat (#3), without denoising (A) and with denoising by PCA(7) (B), PCA(8) (C), PCA(12) (D), MLSVD (E), NLmCED (F), DCAE (G), and DPDL (H). T_1_ map was shown in (I). Arrows in (E) and (F) point to patches of uniform intensity, highlighting the suboptimal performance of the denoising.
**Figure S42:** LD‐fitted APT maps from the leg muscle in a rat (#4), without denoising (A) and with denoising by PCA(7) (B), PCA(8) (C), PCA(12) (D), MLSVD (E), NLmCED (F), DCAE (G), and DPDL (H). T_1_ map was shown in (I). Arrows in (E) and (F) point to patches of uniform intensity, highlighting the suboptimal performance of the denoising.
**Figure S43:** LD‐fitted APT maps from the leg muscle in a rat (#5), without denoising (A) and with denoising by PCA(7) (B), PCA(8) (C), PCA(12) (D), MLSVD (E), NLmCED (F), DCAE (G), and DPDL (H). T_1_ map was shown in (I). Arrows in (E) and (F) point to patches of uniform intensity, highlighting the suboptimal performance of the denoising.
**Figure S44:** LD‐fitted APT maps from the leg muscle in a rat (#6), without denoising (A) and with denoising by PCA(7) (B), PCA(8) (C), PCA(12) (D), MLSVD (E), NLmCED (F), DCAE (G), and DPDL (H). T_1_ map was shown in (I). Arrows in (E) and (F) point to patches of uniform intensity, highlighting the suboptimal performance of the denoising.
**Figure S45:** LD‐fitted PCr maps from the leg muscle in a rat (#1), without denoising (A) and with denoising by PCA(7) (B), PCA(8) (C), PCA(12) (D), MLSVD (E), NLmCED (F), DCAE (G), and DPDL (H). T_1_ map was shown in (I).
**Figure S46:** LD‐fitted PCr maps from the leg muscle in a rat (#2), without denoising (A) and with denoising by PCA(7) (B), PCA(8) (C), PCA(12) (D), MLSVD (E), NLmCED (F), DCAE (G), and DPDL (H). T_1_ map was shown in (I).
**Figure S47:** LD‐fitted PCr maps from the leg muscle in a rat (#3), without denoising (A) and with denoising by PCA(7) (B), PCA(8) (C), PCA(12) (D), MLSVD (E), NLmCED (F), DCAE (G), and DPDL (H). T_1_ map was shown in (I).
**Figure S48:** LD‐fitted PCr maps from the leg muscle in a rat (#4), without denoising (A) and with denoising by PCA(7) (B), PCA(8) (C), PCA(12) (D), MLSVD (E), NLmCED (F), DCAE (G), and DPDL (H). T_1_ map was shown in (I).
**Figure S49:** LD‐fitted PCr maps from the leg muscle in a rat (#5), without denoising (A) and with denoising by PCA(7) (B), PCA(8) (C), PCA(12) (D), MLSVD (E), NLmCED (F), DCAE (G), and DPDL (H). T_1_ map was shown in (I).
**Figure S50:** LD‐fitted PCr maps from the leg muscle in a rat (#6), without denoising (A) and with denoising by PCA(7) (B), PCA(8) (C), PCA(12) (D), MLSVD (E), NLmCED (F), DCAE (G), and DPDL (H). T_1_ map was shown in (I).
**Figure S51:** LD‐fitted guanidine maps from the leg muscle in a rat (#1), without denoising (A) and with denoising by PCA(7) (B), PCA(8) (C), PCA(12) (D), MLSVD (E), NLmCED (F), DCAE (G), and DPDL (H). T_1_ map was shown in (I).
**Figure S52:** LD‐fitted guanidine maps from the leg muscle in a rat (#2), without denoising (A) and with denoising by PCA(7) (B), PCA(8) (C), PCA(12) (D), MLSVD (E), NLmCED (F), DCAE (G), and DPDL (H). T_1_ map was shown in (I). Arrows in (E) and (F) point to patches of uniform intensity, highlighting the suboptimal performance of the denoising.
**Figure S53:** LD‐fitted guanidine maps from the leg muscle in a rat (#3), without denoising (A) and with denoising by PCA(7) (B), PCA(8) (C), PCA(12) (D), MLSVD (E), NLmCED (F), DCAE (G), and DPDL (H). T_1_ map was shown in (I). Arrows in (E) and (F) point to patches of uniform intensity, highlighting the suboptimal performance of the denoising.
**Figure S54:** LD‐fitted guanidine maps from the leg muscle in a rat (#4), without denoising (A) and with denoising by PCA(7) (B), PCA(8) (C), PCA(12) (D), MLSVD (E), NLmCED (F), DCAE (G), and DPDL (H). T_1_ map was shown in (I). Arrows in (E) and (F) point to patches of uniform intensity, highlighting the suboptimal performance of the denoising.
**Figure S55:** LD‐fitted guanidine maps from the leg muscle in a rat (#5), without denoising (A) and with denoising by PCA(7) (B), PCA(8) (C), PCA(12) (D), MLSVD (E), NLmCED (F), DCAE (G), and DPDL (H). T_1_ map was shown in (I). Arrows in (E) and (F) point to patches of uniform intensity, highlighting the suboptimal performance of the denoising.
**Figure S56:** LD‐fitted guanidine maps from the leg muscle in a rat (#6), without denoising (A) and with denoising by PCA(7) (B), PCA(8) (C), PCA(12) (D), MLSVD (E), NLmCED (F), DCAE (G), and DPDL (H). T_1_ map was shown in (I). Arrows in (E) and (F) point to patches of uniform intensity, highlighting the suboptimal performance of the denoising.
**Figure S57:** LD‐fitted NOE(−3.5) maps from the leg muscle in a rat (#1), without denoising (A) and with denoising by PCA(7) (B), PCA(8) (C), PCA(12) (D), MLSVD (E), NLmCED (F), DCAE (G), and DPDL (H). T_1_ map was shown in (I). Arrows in (E) and (F) point to patches of uniform intensity, highlighting the suboptimal performance of the denoising.
**Figure S58:** LD‐fitted NOE(−3.5) maps from the leg muscle in a rat (#2), without denoising (A) and with denoising by PCA(7) (B), PCA(8) (C), PCA(12) (D), MLSVD (E), NLmCED (F), DCAE (G), and DPDL (H). T_1_ map was shown in (I).
**Figure S59:** LD‐fitted NOE(−3.5) maps from the leg muscle in a rat (#3), without denoising (A) and with denoising by PCA(7) (B), PCA(8) (C), PCA(12) (D), MLSVD (E), NLmCED (F), DCAE (G), and DPDL (H). T_1_ map was shown in (I). Arrows in (E) and (F) point to patches of uniform intensity, highlighting the suboptimal performance of the denoising.
**Figure S60:** LD‐fitted NOE(−3.5) maps from the leg muscle in a rat (#4), without denoising (A) and with denoising by PCA(7) (B), PCA(8) (C), PCA(12) (D), MLSVD (E), NLmCED (F), DCAE (G), and DPDL (H). T_1_ map was shown in (I).
**Figure S61:** LD‐fitted NOE(−3.5) maps from the leg muscle in a rat (#5), without denoising (A) and with denoising by PCA(7) (B), PCA(8) (C), PCA(12) (D), MLSVD (E), NLmCED (F), DCAE (G), and DPDL (H). T_1_ map was shown in (I). Arrows in (E) and (F) point to patches of uniform intensity, highlighting the suboptimal performance of the denoising.
**Figure S62:** LD‐fitted NOE(−3.5) maps from the leg muscle in a rat (#6), without denoising (A) and with denoising by PCA(7) (B), PCA(8) (C), PCA(12) (D), MLSVD (E), NLmCED (F), DCAE (G), and DPDL (H). T_1_ map was shown in (I). Arrows in (E) and (F) point to patches of uniform intensity, highlighting the suboptimal performance of the denoising.
**Table S1:** List of all sample parameters in the four‐pool (water, solute #1, solute #2, and MT) model Bloch simulations.
**Table S2:** List of all sample parameters in a seven‐pool model simulation to generate digital phantoms for mimicking brain tissues.
**Table S3:** Starting points and boundaries of the amplitude, width, and offset of the water and MT pools in the LD analysis. The unit of peak width and offset is ppm.
**Table S4:** Summary of computational complexity using big‐O notation, encompassing both time and space complexities, for various data analysis and DL methods applied to input data with size W × H × *N*. Here, W × H represents the spatial size of the CEST image, and N denotes the length of the Z‐spectral dimension. In this study, W = 64, H = 64, and *N* = 89. For simplicity, we assume (H ≈ W ≈ N), which simplifies the analysis and enables a more straightforward comparison across methods. For DL models, the time complexity is divided into training time complexity and testing time complexity. Space complexity refers to memory requirements.

## Data Availability

All source code for processing and analysis, along with the associated data, is available on our lab's GitHub repository: https://github.com/CESTlabZu.
