# Supplementary material for: Denoising Low‐Power CEST Imaging Using a Deep Learning Approach With a Dual‐Power Feature Preparation Strategy
Source: Magn Reson Med. 2025 Oct 13;95(3):1410–28. doi: 10.1002/mrm.70124 (PMC12746379; doi:10.1002/mrm.70124)
Supplement: Supplementary file 1 — Figure S1: (A) A pair of clean LP Z‐spectrum and clean HP Z‐spectrum from a modified two‐pool (amide and water) model simulation. (B) The clean LP Z‐spectrum and the corresponding transformed HP Z‐spectrum from a single 1 × 5 Z‐spectral window. (C) The corresponding transformed HP Z‐spectrum from a single 1 × 5 Z‐spectral window and that from a single 1 × 3 Z‐spectral window. (D) The corresponding transformed HP Z‐spectrum from a single 1 × 5 Z‐spectral window and that from a single shifted 1 × 5 Z‐spectral window. (E) The corresponding transformed HP Z‐spectrum from a single 1 × 3 Z‐spectral window and that from a single shifted 1 × 3 Z‐spectral window. (F) The corresponding transformed HP Z‐spectrum averaged from both the 1 × 5 and 1 × 3 Z‐spectral windows, including their shifted Z‐spectral windows, which is simply referred to as the “transformed HP Z‐spectrum”, unless otherwise specified. (G) A section (from 3.5 ppm to 4.0 ppm) of the clean LP Z‐spectrum, the corresponding transformed HP Z‐spectrum from a single 1 × 5 Z‐spectral window, and corresponding transformed HP Z‐spectrum, with fitted lines. (H) Monte Carlo simulation of the feature angle from the Z‐spectra in (G). The feature angle is determined by fitting CEST signals within this frequency range to a line and then calculating the angle between this fitted line and the x‐axis. This feature angle can roughly reflect the mean steepness of the Z‐spectrum within this frequency range, offering a straightforward method for comparing slopes of the Z‐spectrum. Due to the different units on the x‐axis and y‐axis, a real angle degree cannot be calculated. Therefore, we define the feature angle from the LP Z‐spectrum as 1 unit, and express other feature angles as multiples of this unit. (I) Violin plot showing the ratio of all feature angles to their standard deviation for the Z‐spectra in (G) from the Monte Carlo simulation. The sample parameters for this modified two‐pool model simulation are based on Table S2, [file MRM-95-1410-s001.docx]

**Supplementary information**

**Supporting information equations.**

MSE, MAE, PSNR, and SSIM are defined in following Eq. (S1-S4), respectively

$MSE=\left\| \hat{s}-s \right\|_{2}^{2}$ (S1)

$MAE=\left| \hat{s}-s \right|$ (S2)

where $s$ and $\hat{s}$ denote the GT signals and estimated signals, respectively.

$PSNR(dB) = 10\log_{10} \frac{X_{max}^{2}}{MSE}$ (S3)

where $X$ represents a given sample data which is either noisy or denoised, and $X_{max}$ is the maximum value in this given sample data, which is 1 for a Z-spectrum. To clearly visualize the subtle differences in PSNR, we apply an exponential function to the PSNR values, referred to as Exp(PSNR).

$SSIM$ = $\frac{\left( 2\mu_{X_{denoise}} \mu_{X_{ref}}+ c_{1} \right)\left( 2\sigma_{X_{denoise}X_{ref}} + c_{2} \right)}{\left( \mu_{X_{denoise}}^{2}+ \mu_{X_{ref}}^{2}+c_{1} \right)\left( \sigma_{X_{denoise}}^{2}+\sigma_{X_{ref}}^{2}+ c_{2} \right)}$ (S4)

where $\mu$ refers the mean and $\sigma$ stands for the standard deviation of X with the subscript “denoise” indicating the denoised data, while “ref” indicating the reference data; $\sigma_{X_{denoise}X_{ref}}$ is the covariance of $X_{denoise}$ and $X_{ref}$. The constants $c_{1}=1\times{10}^{-4}$ and $c_{2}=9c_{1}$ are taken to avoid the division by zero(1).

**Supporting information Table S1.** List of all sample parameters in the four-pool (water, solute #1, solute #2, and MT) model Bloch simulations.

| **Sample parameters** | **Water** | **Solute #1** | **Solute #2** | **MT** |
| --- | --- | --- | --- | --- |
| f_s_ (%) | 100 | 0.05, 0.1, 0.15, 5 | 0.05, 0.1, 0.15, 5 | 4:6:16 |
| k_sw_ (s^-1^) | - | 50, 100, 200,500, 2000, 5000 | 50, 100, 200, 500, 2000, 5000 | 25 |
| T_1_ (s) | 1.5 | 1.5 | 1.5 | 1.5 |
| T_2_ (ms) | 50 | 0.5, 2, 10, 20 | 0.5, 2, 10, 20 | 0.05 |
| **Δ** (ppm) | 0 | 4.375: 0.5: -4.125 | 3.75: 0.5: -3.75 | -2.3 |

Δω values were shifted by: -0.4ppm, -0.2ppm, 0ppm, 0.2ppm, and 0.4ppm

ω_1_ values were scaled by: 0.8, 0.9, 1, 1.1, and 1.2

**Supporting information Table S2.** List of all sample parameters in a seven-pool model simulation to generate digital phantoms for mimicking brain tissues.

| **Sample parameters** | **Water** | **Amide** | **Amine** | **Guan** | **NOE(-1.6)** | **NOE(-3.5)** | **MT** |
| --- | --- | --- | --- | --- | --- | --- | --- |
| **f_s_ (%)** | 100 | 0.04: 0.03: 0.16 | 0.5, 1.0 | 0.03 | 0.6 | 0.6,1.0, 1.4 | 5:5:15 |
| **k_sw_ (s^-1^)** | - | 50:50:200^a^ | 3000,5000^b^ | 500^c^ | 50 | 20 | 25 |
| **T_1_ (s)** | 1.5 | 1.5 | 1.5 | 1.5 | 1.5 | 1.5 | 1.5 |
| **T_2_ (ms)** | 50 | 2, 3.5 | 10 | 10 | 1 | 0.3,0.5 | 0.05 |
| **Δ (ppm)** | 0 | 3.5 | 3 | 2 | -1.6 | -3.5 | -2.3 |
| Guan: Guanidine | | | | | | | |

Δω values were shifted by: -0.3ppm, 0ppm, 0.3ppm

ω_1_ values were scaled by: 0.85, 1, and 1.15

^a^ Ref (2-4), ^b^ Ref (5), ^c^ Ref (6)

**Supporting information Table S3.** Starting points and boundaries of the amplitude, width, and offset of the water and MT pools in the LD analysis. The unit of peak width and offset is ppm. The goodness of the LD fitting was assessed by the sum of squared errors.

|  | Start | Lower | Upper |
| --- | --- | --- | --- |
| A_water_ | 0.9 | 0.02 | 1 |
| W_water_ | 1.4 | 0.3 | 10 |
| Δ_water_ | 0 | -1 | 1 |
| A_MT_ | 0.1 | 0 | 1 |
| W_MT_ | 25 | 10 | 100 |
| Δ_MT_ | 0 | -4 | 4 |

**Supporting information Table S4:** Summary of computational complexity using big-O notation, encompassing both time and space complexities, for various data analysis and DL methods applied to input data with size W×H×N. Here, W×H represents the spatial size of the CEST image, and N denotes the length of the Z-spectral dimension. In this study, W = 64, H = 64, and N = 89. For simplicity, we assume (H ≈ W ≈ N), which simplifies the analysis and enables a more straightforward comparison across methods. For DL models, the time complexity is divided into training time complexity and testing time complexity. Space complexity refers to memory requirements.

| **Methods** | **Time complexity** | **Space complexity** |
| --- | --- | --- |
| PCA (7) | O(N^3^) | O(N^2^) |
| MLSVD (7) | O(N^3^) | O(N^2^) |
| NLmCED (8) | O(N^3^) | O(N^3^) |
| DCAE (9) | Training: O(N^4^)/ testing: O(N^3^) | O(N^3^) |
| DPDL (9) | Training: O(N^4^)/ testing: O(N^3^) | O(N^3^) |
| **Explanation of PCA and MLSVD complexity:**  Time complexity: PCA involves eigen-decomposition or Singular Value Decomposition (SVD) applied to a covariance matrix derived from input data, which has dimensions N×N. The time complexity of this process scales as O(N³). MLSVD is a form of tensor decomposition, and if the tensor dimensions scale similarly, the computational complexity remains O(N³).  Space complexity: PCA requires storage for the covariance matrix, resulting in O(N^2^) space complexity. MLSVD similarly requires storage for matrices during decomposition, which again scales as O(N^2^).  **Explanation of DL model complexity:** In CNNs, the primary computational complexity arises from the convolutional layers. In contrast, other layers, such as activation functions, pooling layers, and fully connected layers, contribute comparatively less to the overall complexity. The time complexity of a single convolutional layer can be approximately as O(1×N×C×K×1×F), where C denotes the number of input channels, K is the number of kernels, and 1×F corresponds to the kernel size. When the values of C, K, and 1×F are relatively small (e.g., C = 1, K = 32, 1×F = 1×3), the number of multiplications per voxel becomes O(1×N×96). Assuming a network with 6 convolutional layers and considering the internal weight calculations for each layer introduce an additional factor of N, the overall computational complexity scales as O(1×N×N×96) ≈ O(N³).  *Training Time Complexity:* The computational complexity for a single epoch is dominated by convolutional operations, which scale as O(N³). If there are n epochs used to train the model, the time complexity scales linearly with n. Assuming n=N, the overall complexity is O(N^4^).  *Testing Time Complexity:* During testing, only forward propagation is required, which involves convolutional operations that scale as O(N³), like the complexity of a single epoch during training.  *Space Complexity:* DL requires storage of internal weight, biases, and gradients for 6 layers, result in an approximate space complexity of O(N³). | | |


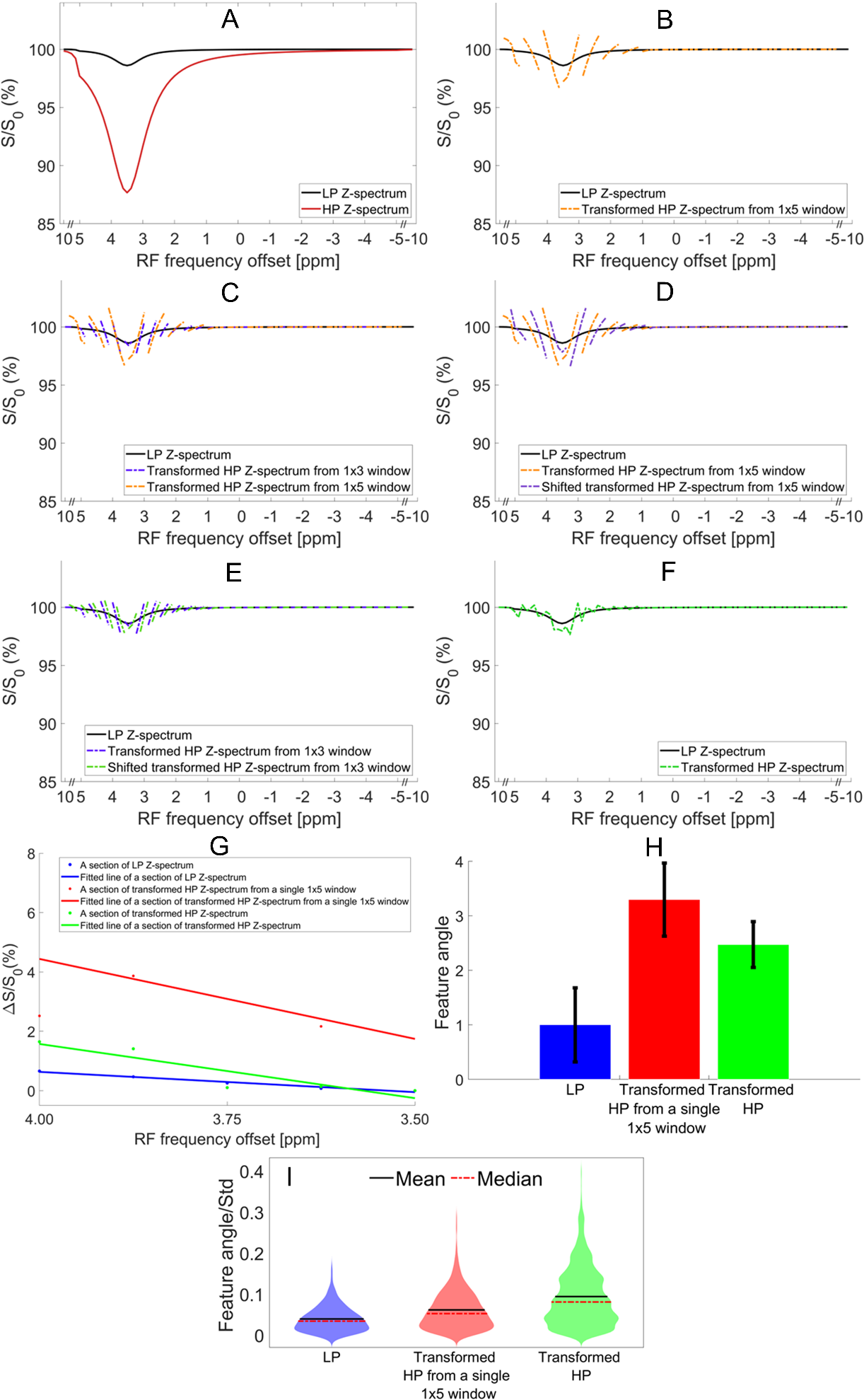


**Supporting information Fig. S1:** (A) A pair of clean LP Z-spectrum and clean HP Z-spectrum from a modified two-pool (amide and water) model simulation. (B) The clean LP Z-spectrum and the corresponding transformed HP Z-spectrum from a single 1x5 Z-spectral window. (C) The corresponding transformed HP Z-spectrum from a single 1x5 Z-spectral window and that from a single 1x3 Z-spectral window. (D) The corresponding transformed HP Z-spectrum from a single 1x5 Z-spectral window and that from a single shifted 1x5 Z-spectral window. (E) The corresponding transformed HP Z-spectrum from a single 1x3 Z-spectral window and that from a single shifted 1x3 Z-spectral window. (F) The corresponding transformed HP Z-spectrum averaged from both the 1x5 and 1x3 Z-spectral windows, including their shifted Z-spectral windows, which is simply referred to as the "transformed HP Z-spectrum", unless otherwise specified. (G) A section (from 3.5ppm to 4.0ppm) of the clean LP Z-spectrum, the corresponding transformed HP Z-spectrum from a single 1x5 Z-spectral window, and corresponding transformed HP Z-spectrum, with fitted lines. (H) Monte Carlo simulation of the feature angle from the Z-spectra in (G). The feature angle is determined by fitting CEST signals within this frequency range to a line and then calculating the angle between this fitted line and the x-axis. This feature angle can roughly reflect the mean steepness of the Z-spectrum within this frequency range, offering a straightforward method for comparing slopes of the Z-spectrum. Due to the different units on the x-axis and y-axis, a real angle degree cannot be calculated. Therefore, we define the feature angle from the LP Z-spectrum as 1 unit, and express other feature angles as multiples of this unit. (I) Violin plot showing the ratio of all feature angles to their standard deviation for the Z-spectra in (G) from the Monte Carlo simulation. The sample parameters for this modified two-pool model simulation are based on Supporting information Table S2, with the following modifications to highlight the APT peak: (1) a significantly higher f_s_ of 1% was simulated to emphasize the APT peak; (2) the DS effect was removed by nulling ω_1_ applied on water in Bloch equations. Additionally, k_sw_ was set to 50s^-1^, and T_2s_ was set to 2ms. The Monte Carlo simulation was performed by adding 3% Gaussian noise to the clean Z-spectra for 1000 times, and the feature angle was calculated.


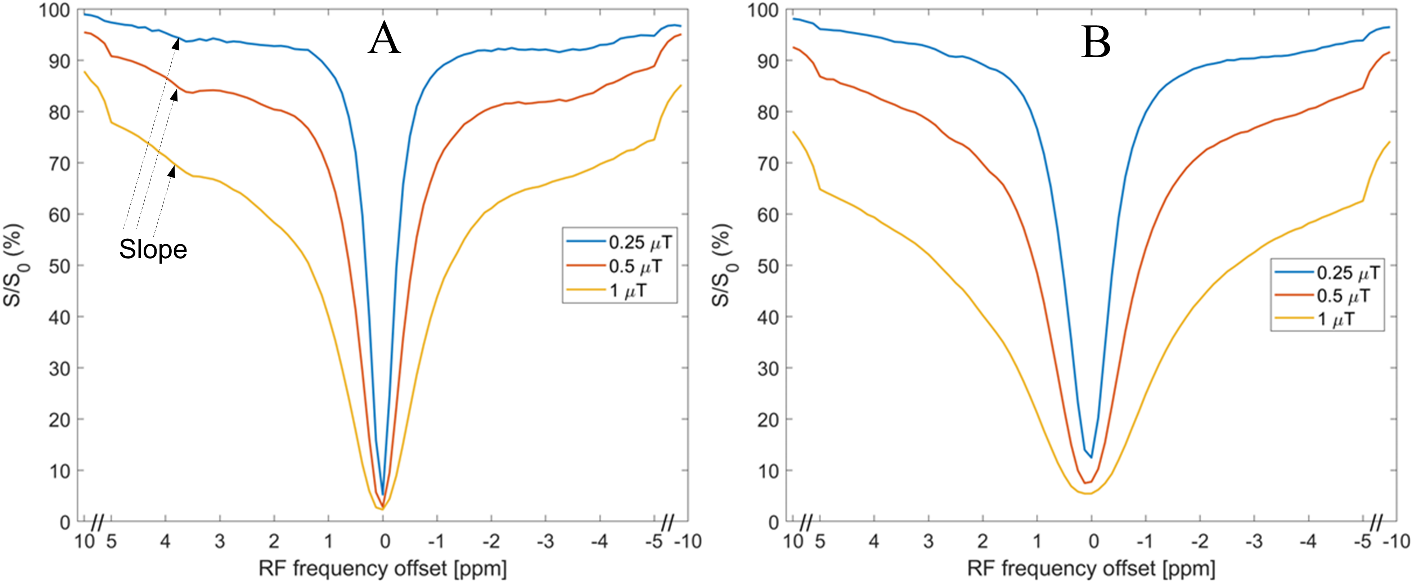


**Supporting information Fig. S2:** Mean measured CEST Z-spectra from brains of 6 rats bearing 9l tumor (A) and leg muscle tissues of six healthy rats (B) at 4.7T, using saturation fields of 0.25µT, 0.5µT, and 1.0µT, respectively.

**
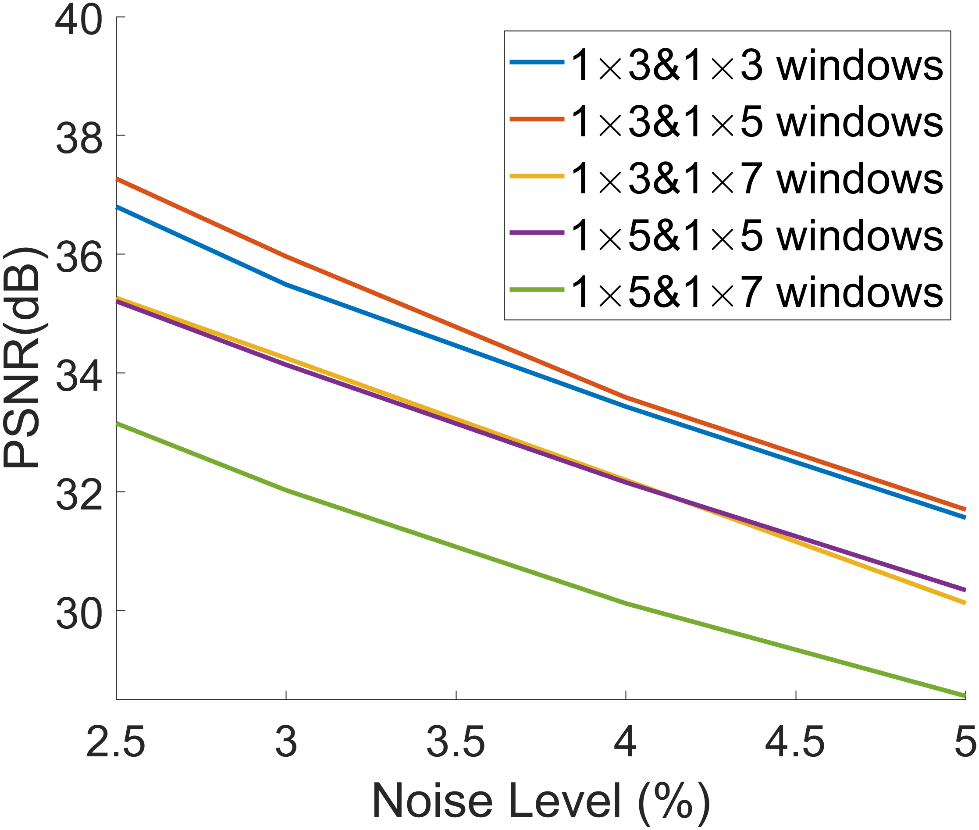
**

**Supporting information Fig.S3:** Comparative analysis of PSNR values of the DPDL-predicted Z-spectra from the digital phantom, for various combinations of Z-spectral window length across different noise levels. The PSNR values are averaged across all voxels in the digital phantom.


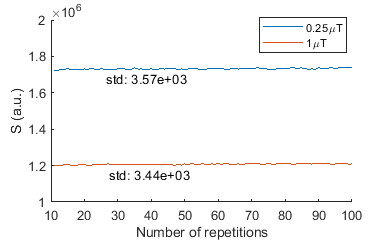


**Supporting information Fig. S4:** Measured CEST signals at 3.5ppm from the whole brain of a rat over 100 repetitions with saturation field strengths of 0.25µT and 1µT, and at 4.7T. To calculate the standard deviation (std), the first 10 repetitions were excluded to avoid transient effects. The std values are roughly similar for both saturation field strengths, suggesting that the CEST signals for these two saturation field strengths have comparable noise levels.


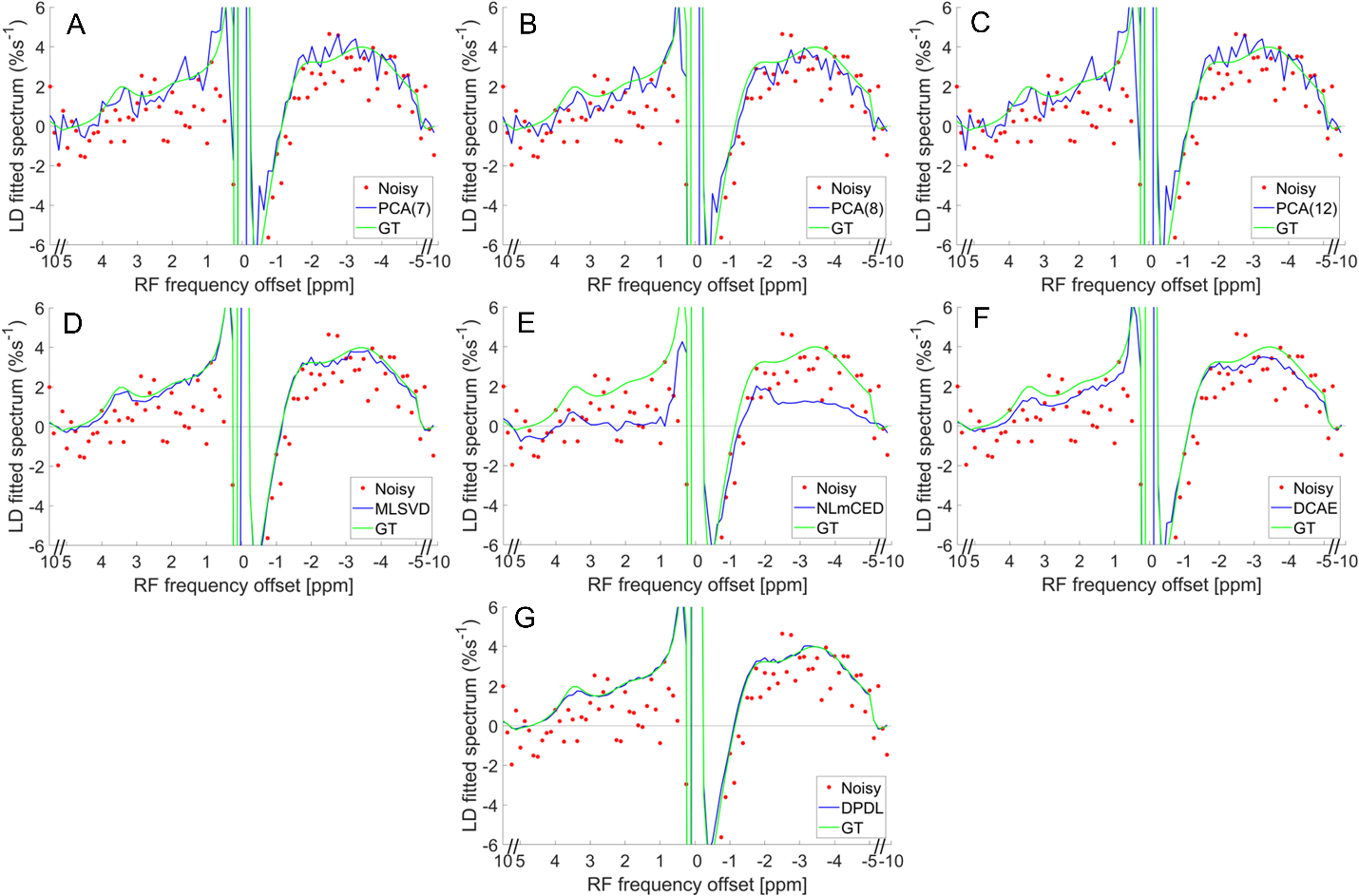


**Supporting information Fig. S5:** A sample LP LD-fitted spectrum from a single voxel in the noisy digital phantom, denoised using various methods: PCA(7) (A), PCA(8) (B), PCA(12) (C), MLSVD (D), NLmCED (E), DCAE (F), and DPDL (G). The MSE values between the LD spectrum fitted from the GT Z-spectrum and those fitted from the denoised Z-spectra are 0.00046 (A), 0.00054 (B), 0.00062 (C), 0.00043 (D), 0.00085 (E), 0.00061 (F), and 0.00023 (G), respectively.


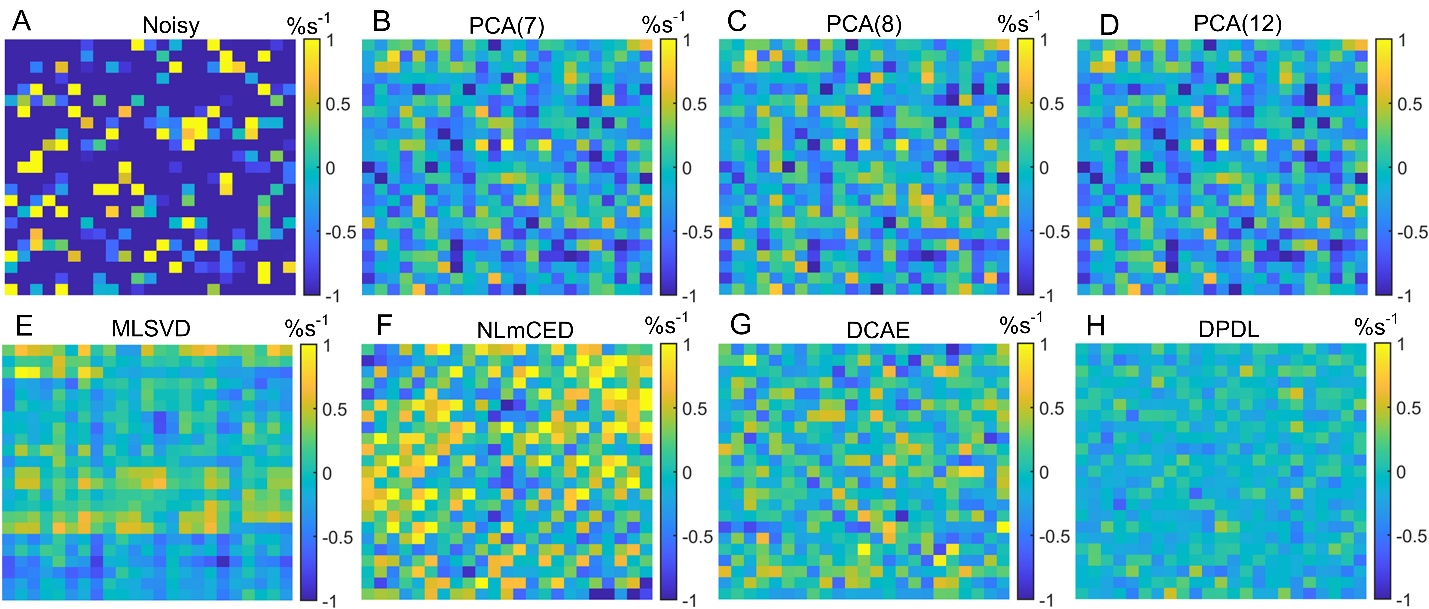


**Supporting information Fig. S6:** Residual maps showing the differences between the APT map from the GT digital phantom and that from various sources: the noisy digital phantom (A), the noisy digital phantom denoised by PCA(7) (B), PCA(8) (C), PCA(12) (D), MLSVD (E), NLmCED (F), DCAE (G), and DPDL (H). The mean absolute values of the residual maps are 0.7774, 0.3266, 0.3274, 0.3383, 0.2484, 0.4437, 0.2350, and 0.1189 for noisy, PCA, MLSVD, NLmCED, DCAE, and DPDL, respectively.


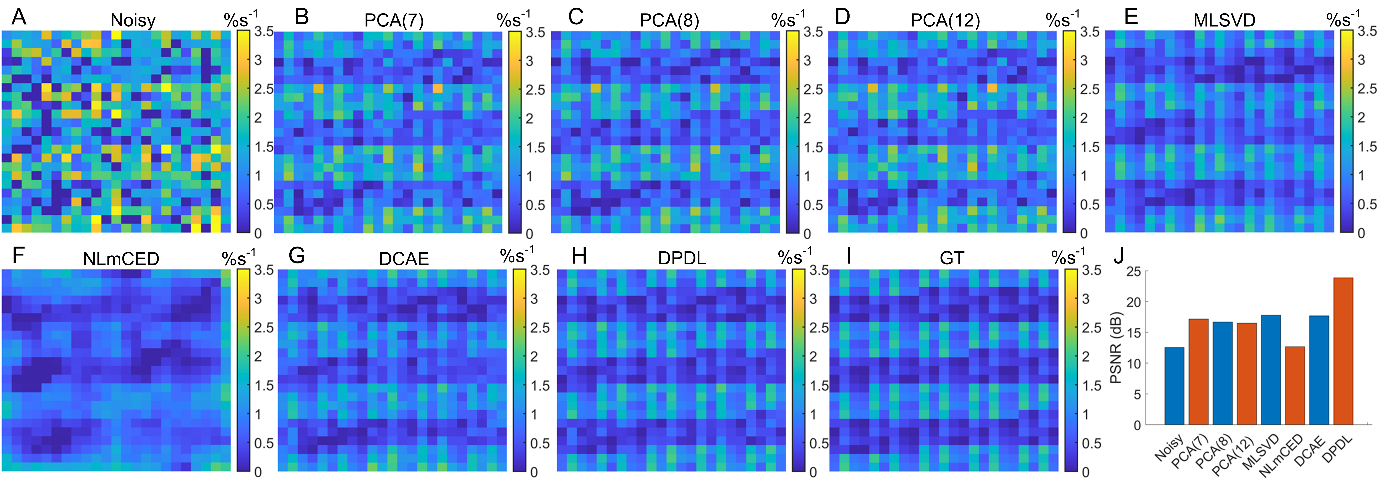


**Supporting information Fig. S7:** (A-I) LD-fitted guanidine maps from the noisy digital phantom, the noisy digital phantom denoised by PCA(7), PCA(8), PCA(12), MLSVD, NLmCED, DCAE, and DPDL methods, as well as the GT digital phantom. (J) PSNR values between the LD-fitted guanidine map from the GT digital phantom and that from the noisy digital phantom [12.66dB], as well as those denoised by PCA(7) [18.24 dB], PCA(8) [17.49 dB], PCA(12) [17.34 dB], MLSVD [18.045 dB], NLmCED [12.715 dB], DCAE [18.425 dB], and DPDL [24.22 dB] methods.


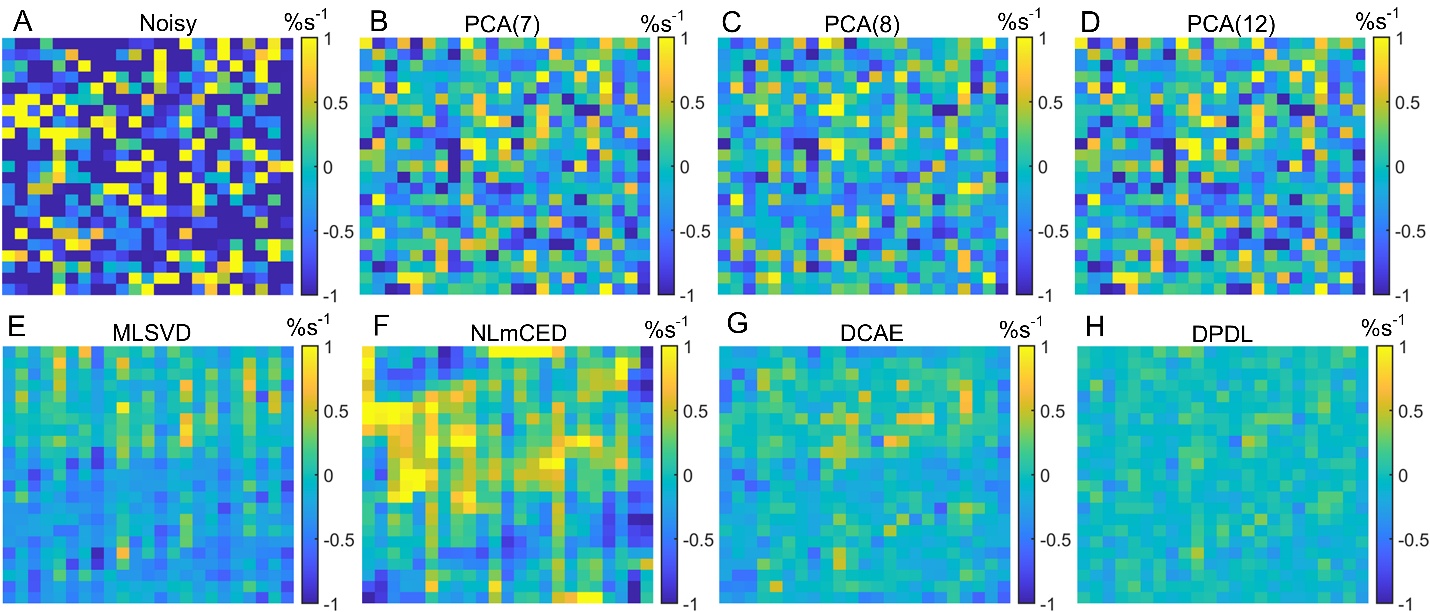


**Supporting information Fig. S8:** Residual maps showing the differences between the guanidine map from the GT digital phantom and that from various sources: the noisy digital phantom (A), the noisy digital phantom denoised by PCA(7) (B), PCA(8) (C), PCA(12) (D), MLSVD (E), NLmCED (F), DCAE (G), and DPDL (H). The mean absolute values of the residual maps are 0.732, 0.328, 0.334, 0.338, 0.261, 0.444, 0.225, and 0.116 for noisy, PCA(7), PCA(8), PCA(12), MLSVD, NLmCED, DCAE, and DPDL, respectively.


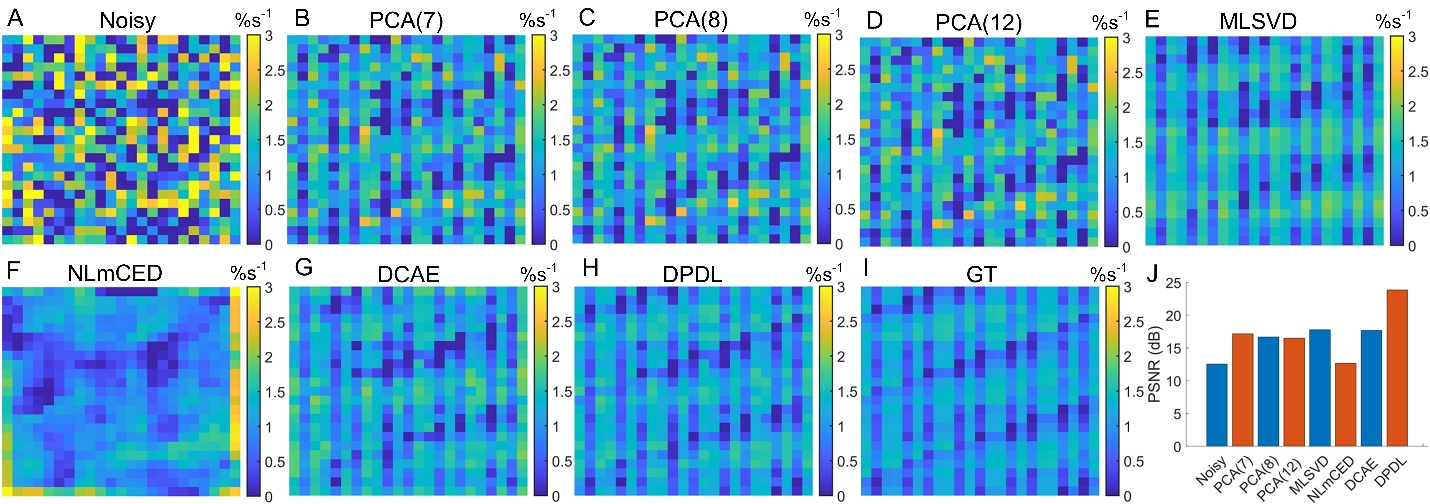


**Supporting information Fig. S9:** (A-I) LD-fitted NOE(-1.6) maps from the noisy digital phantom, the noisy digital phantom denoised by PCA(7), PCA(8), PCA(12), MLSVD, NLmCED, DCAE, and DPDL methods, as well as the GT digital phantom. (J) PSNR values between the LD-fitted NOE(-1.6) map from the GT digital phantom and that from the noisy digital phantom [13.71dB], as well as those denoised by PCA(7) [18.62 dB], PCA(8) [17.83 dB], PCA(12) [17.54 dB], MLSVD [19.01 dB], NLmCED [14.145 dB], DCAE [19.51 dB], and DPDL [24.51 dB] methods.


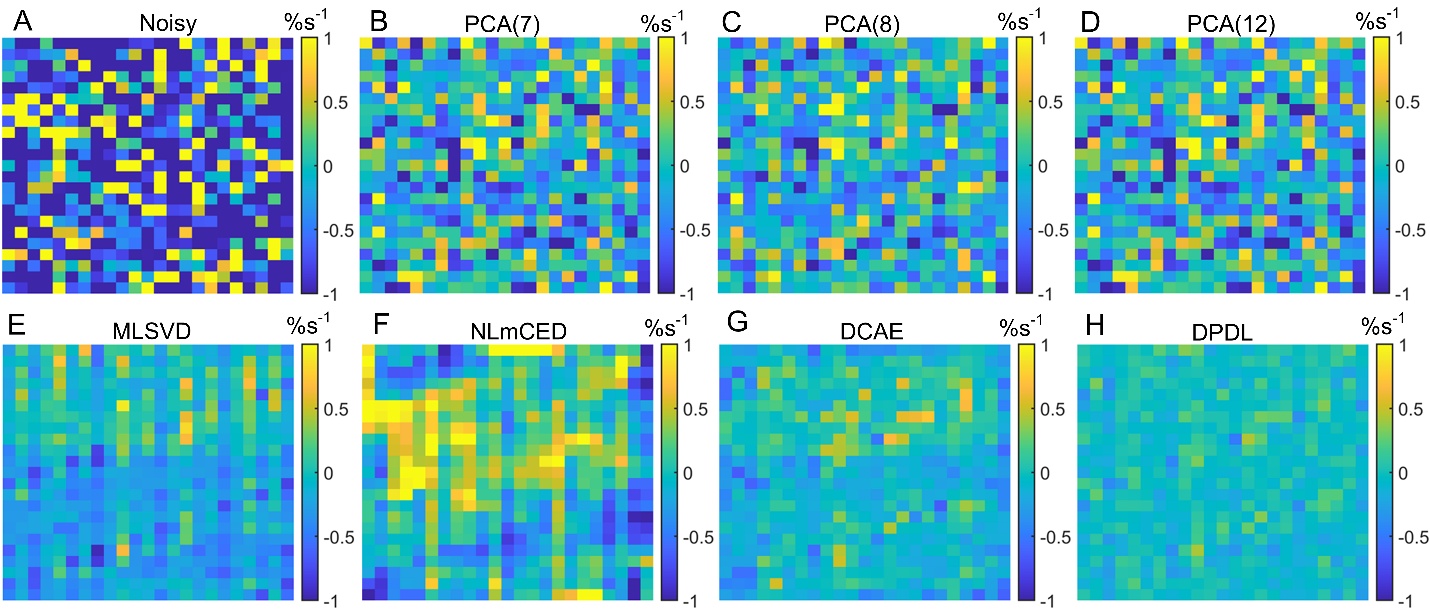


**Supporting information Fig. S10:** Residual maps showing the differences between the NOE(-1.6) map from the GT digital phantom and that from various sources: the noisy digital phantom (A), the noisy digital phantom denoised by PCA(7) (B), PCA(8) (C), PCA(12) (D), MLSVD (E), NLmCED (F), DCAE (G), and DPDL (H). The mean absolute values of the residual maps are 0.772, 0.309, 0.327, 0.348, 0.228, 0.438, 0.226, and 0.129 for noisy, PCA(7), PCA(8), PCA(12), MLSVD, NLmCED, DCAE, and DPDL, respectively.


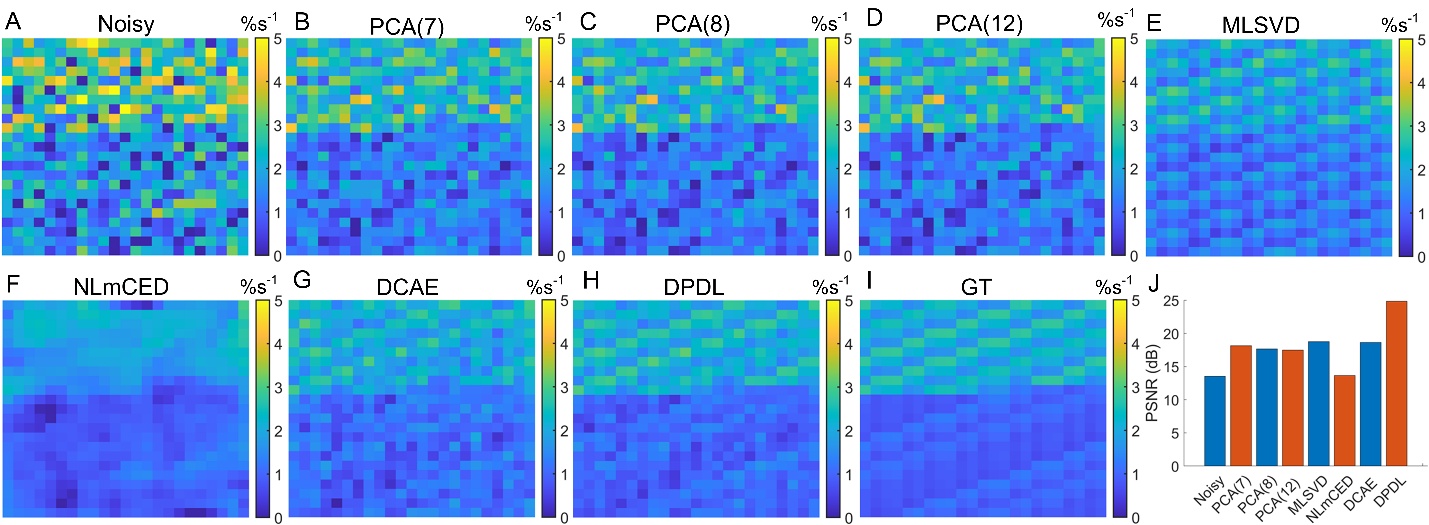


**Supporting information Fig. S11:** (A-I) LD-fitted NOE(-3.5) maps from the noisy digital phantom, the noisy digital phantom denoised by PCA(7), PCA(8), PCA(12), MLSVD, NLmCED, DCAE, and DPDL methods, as well as the GT digital phantom. (J) PSNR values between the LD-fitted NOE(-3.5) map from the GT digital phantom and that from the noisy digital phantom [13.46dB], as well as those denoised by PCA(7) [18.56dB], PCA(8) [18.24dB], PCA(12) [17.92dB], MLSVD [19.705dB], NLmCED [16.58dB], DCAE [20.78dB], and DPDL [26.84dB] methods.


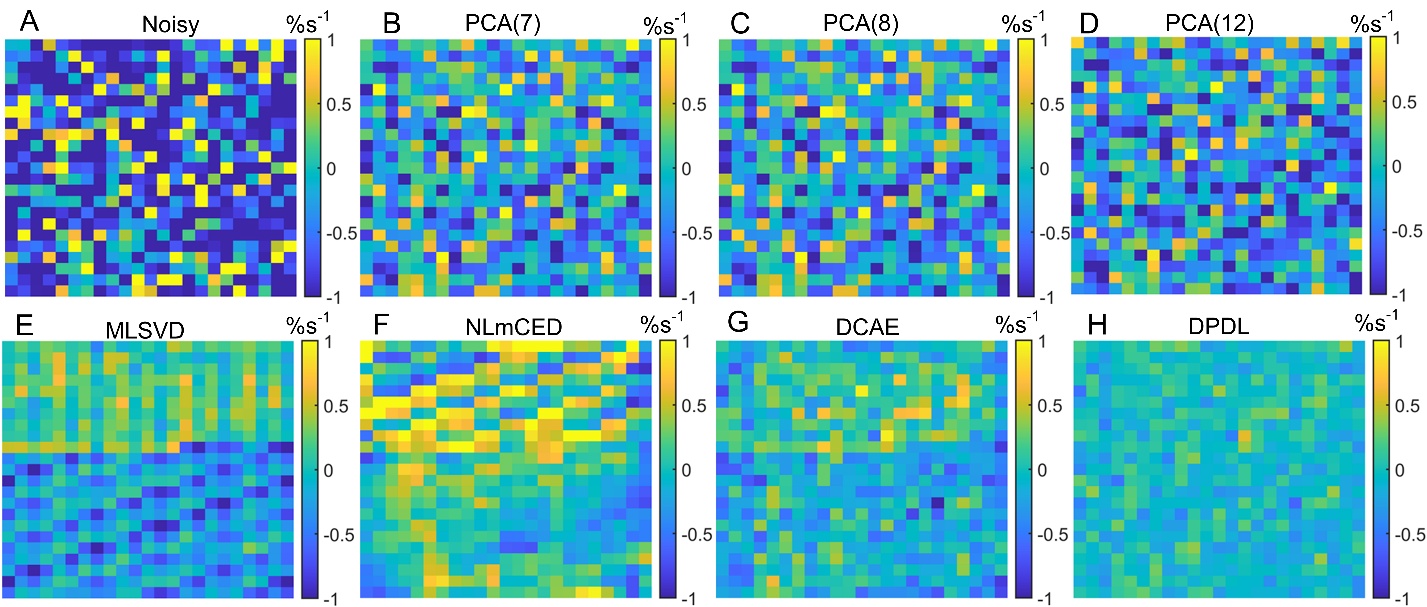


**Supporting information Fig. S12:** Residual maps showing the differences between the NOE(-3.5) map from the GT digital phantom and that from various sources: the noisy digital phantom (A), the noisy digital phantom denoised by PCA(7) (B), PCA(8) (C), PCA(12) (D), MLSVD (E), NLmCED (F), DCAE (G), and DPDL (H). The mean absolute values of the residual maps are 0.692, 0.274, 0.286, 0.293, 0.256, 0.429, 0.275, and 0.112 for noisy, PCA(7), PCA(8), PCA(12), MLSVD, NLmCED, DCAE, and DPDL, respectively.


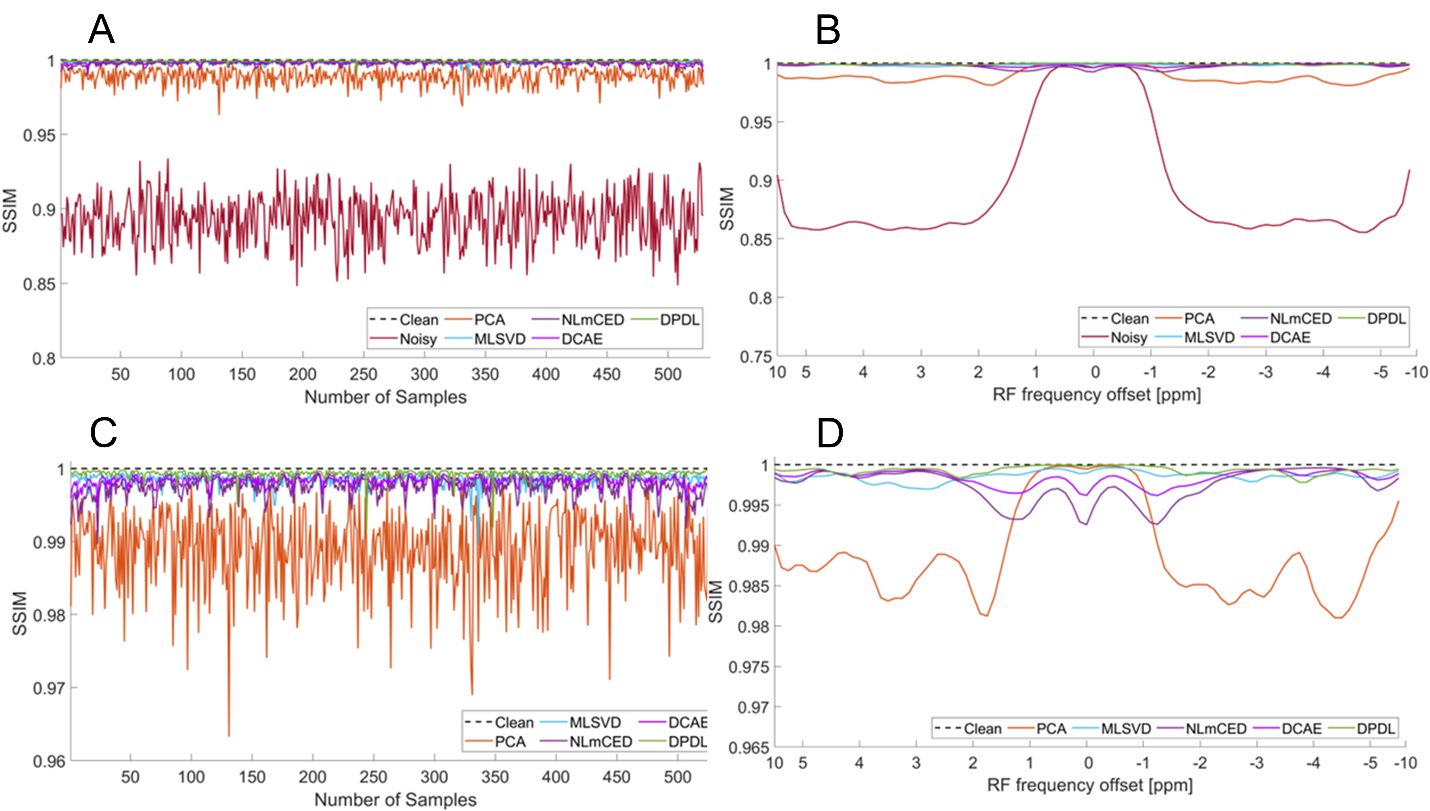


**Supporting information Fig. S13:** (A) Comparison of the SSIM between the denoised Z-spectrum and the corresponding GT from each voxel in the digital phantom among various denoising methods. (B) Comparison of the SSIM between the denoised 23 × 23 matrix in the digital phantom and the corresponding GT at each frequency offset among various denoising methods. (C) and (D) show vertically zoomed versions of (A) and (B), respectively, highlighting the performance of the MLSVD, NLmCED, DCAE, and DPDL methods in comparison to the clean data.


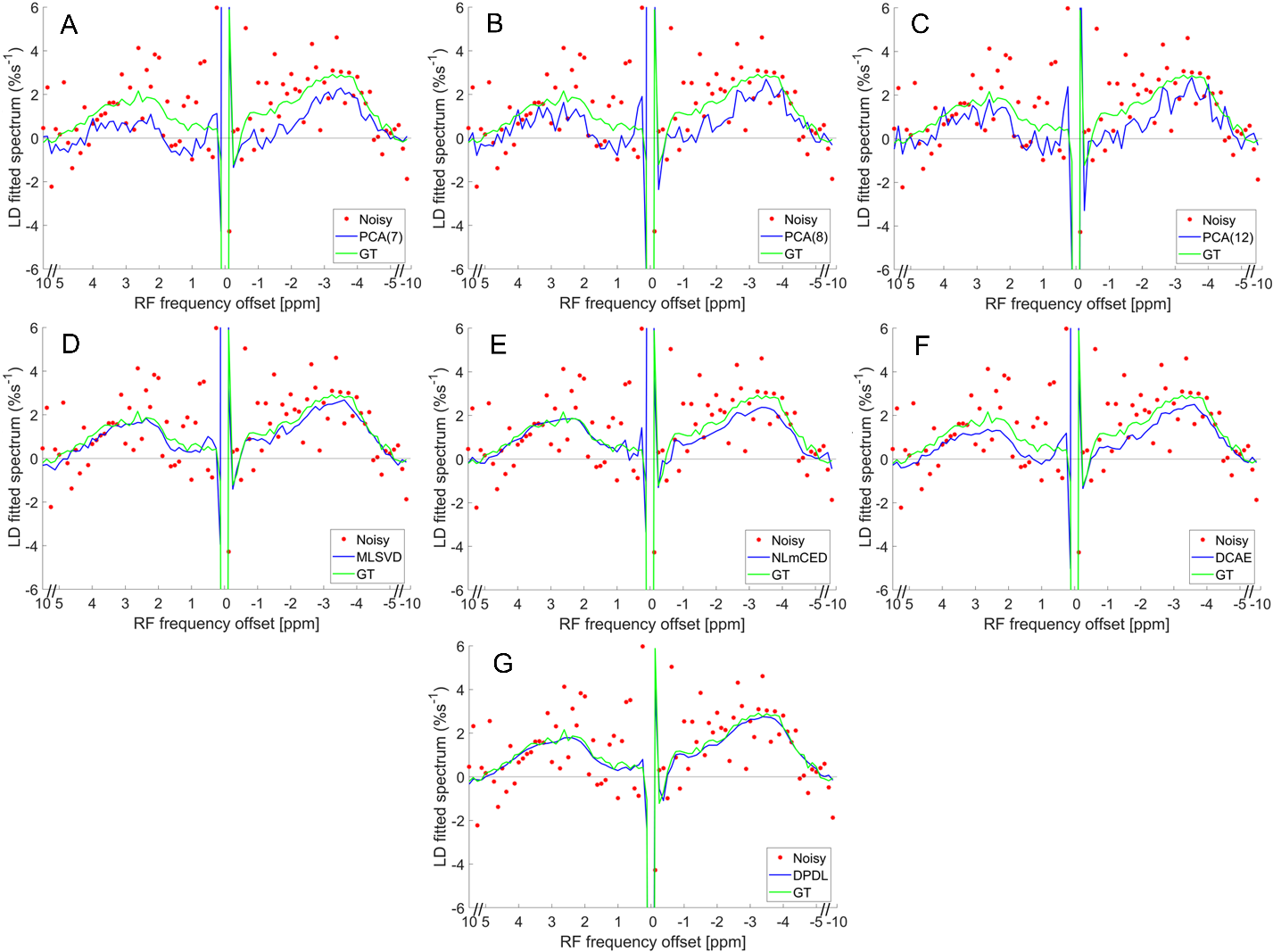


**Supporting information Fig. S14:** A sample LP LD-fitted spectrum from a single voxel in the noisy BSA phantom, denoised using various methods: PCA(7) (A), PCA(8) (B), PCA(12) (C), MLSVD (D), NLmCED (E), DCAE (F), and DPDL (G). The MSE values between the LD spectrum fitted from the GT Z-spectrum and those fitted from the denoised Z-spectra are 0.000390 (A), 0.000426 (B), 0.000414 (C), 0.000372 (D), 0.0002885 (E), 0.000386 (F), and 0.000225 (G), respectively. The noisy LP LD-fitted spectrum and GT are also included in these figures for comparison.


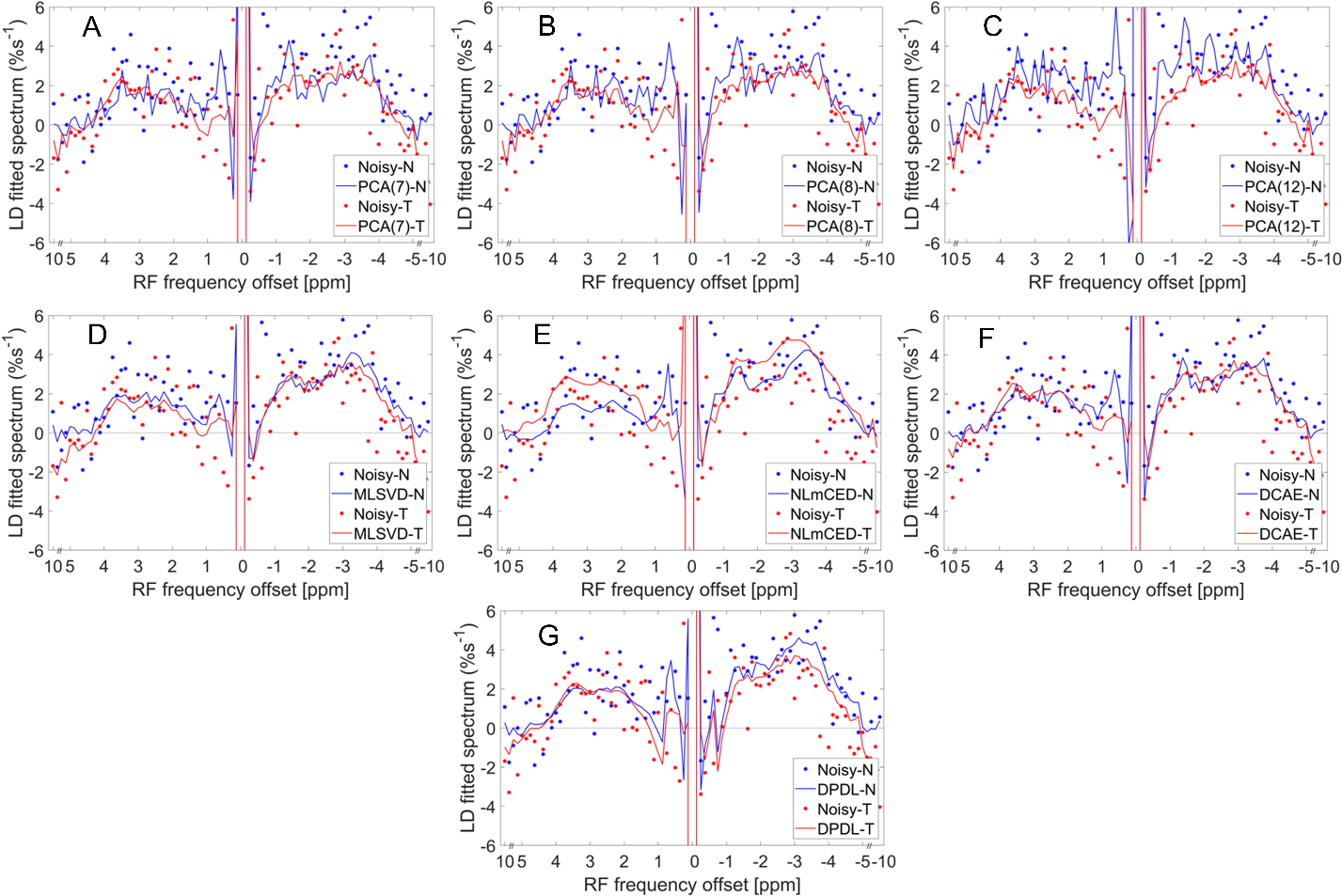


**Supporting information Fig. S15:** A sample LP LD-fitted spectrum from a single voxel in the tumor (T) and a single voxel in the contralateral normal tissue (N) in a representative rat brain (#1), denoised using various methods: PCA(7) (A), PCA(8) (B), PCA(12) (C), MLSVD (D), NLmCED (E), DCAE (F), and DPDL (G). The noisy LP LD-fitted spectra are also included in these figures for comparison.


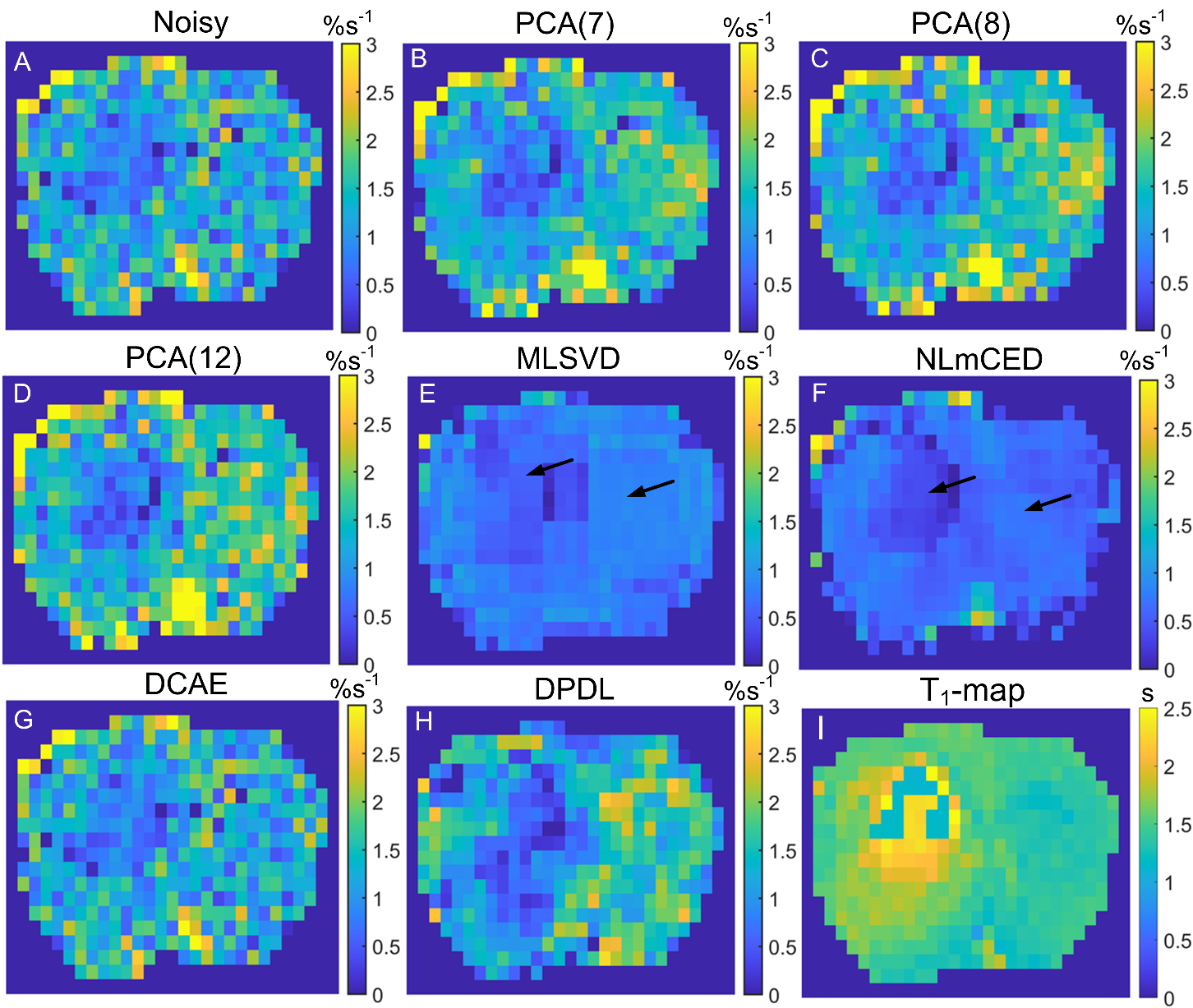


**Supporting information Fig.S16:** LD-fitted APT maps from a rat brain bearing a 9L tumor (#2), without denoising (A) and with denoising by PCA(7) (B), PCA(8) (C), PCA(12) (D), MLSVD (E), NLmCED (F), DCAE (G), and DPDL (H). T_1_ map was shown in (I) to demonstrate the tumor region. Arrows in (E) and (F) point to patches of uniform intensity, highlighting the suboptimal performance of the denoising.


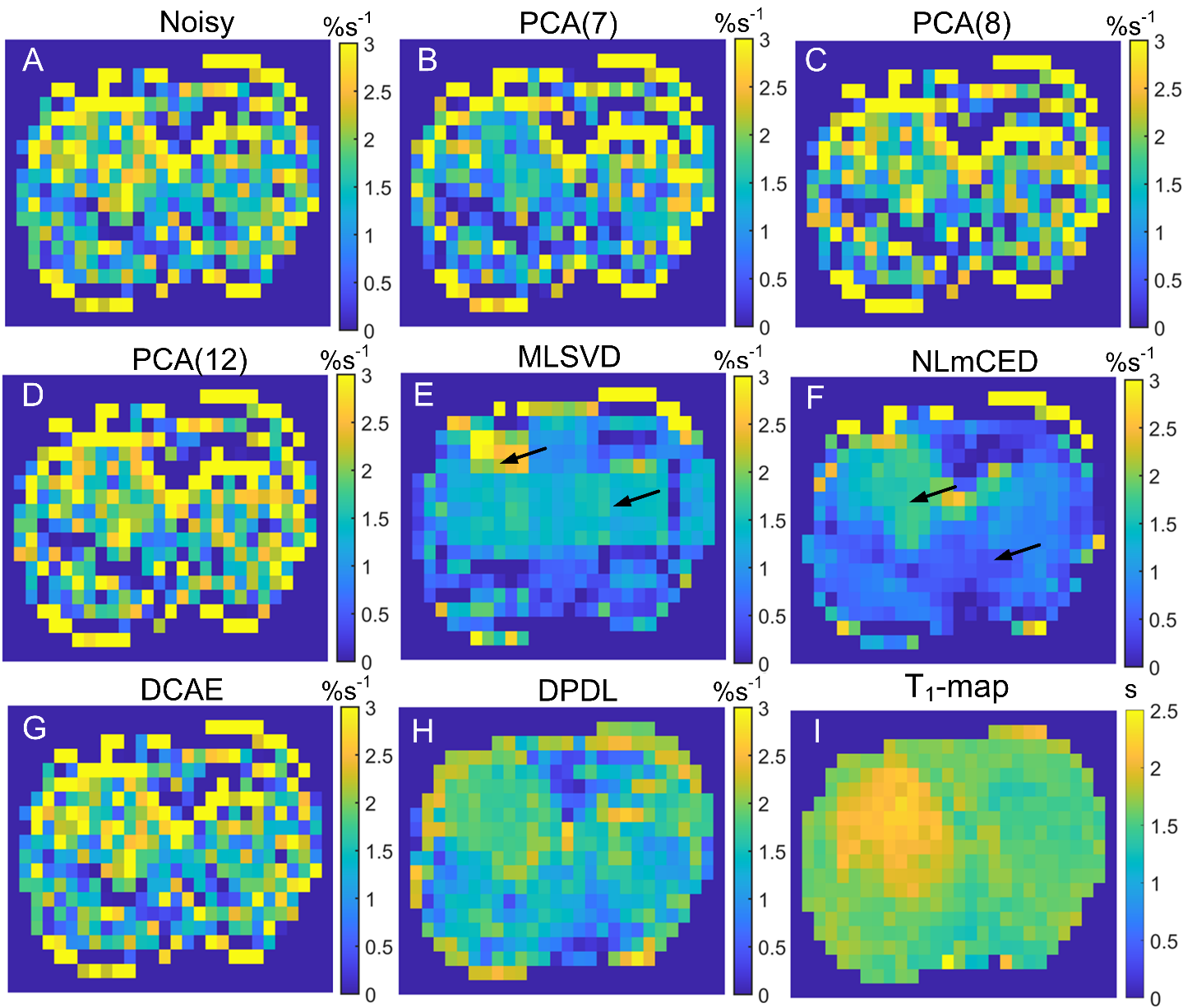


**Supporting information Fig.S17:** LD-fitted APT maps from a rat brain bearing a 9L tumor (#3), without denoising (A) and with denoising by PCA(7) (B), PCA(8) (C), PCA(12) (D), MLSVD (E), NLmCED (F), DCAE (G), and DPDL (H). T_1_ map was shown in (I) to demonstrate the tumor region. Arrows in (E) and (F) point to patches of uniform intensity, highlighting the suboptimal performance of the denoising.


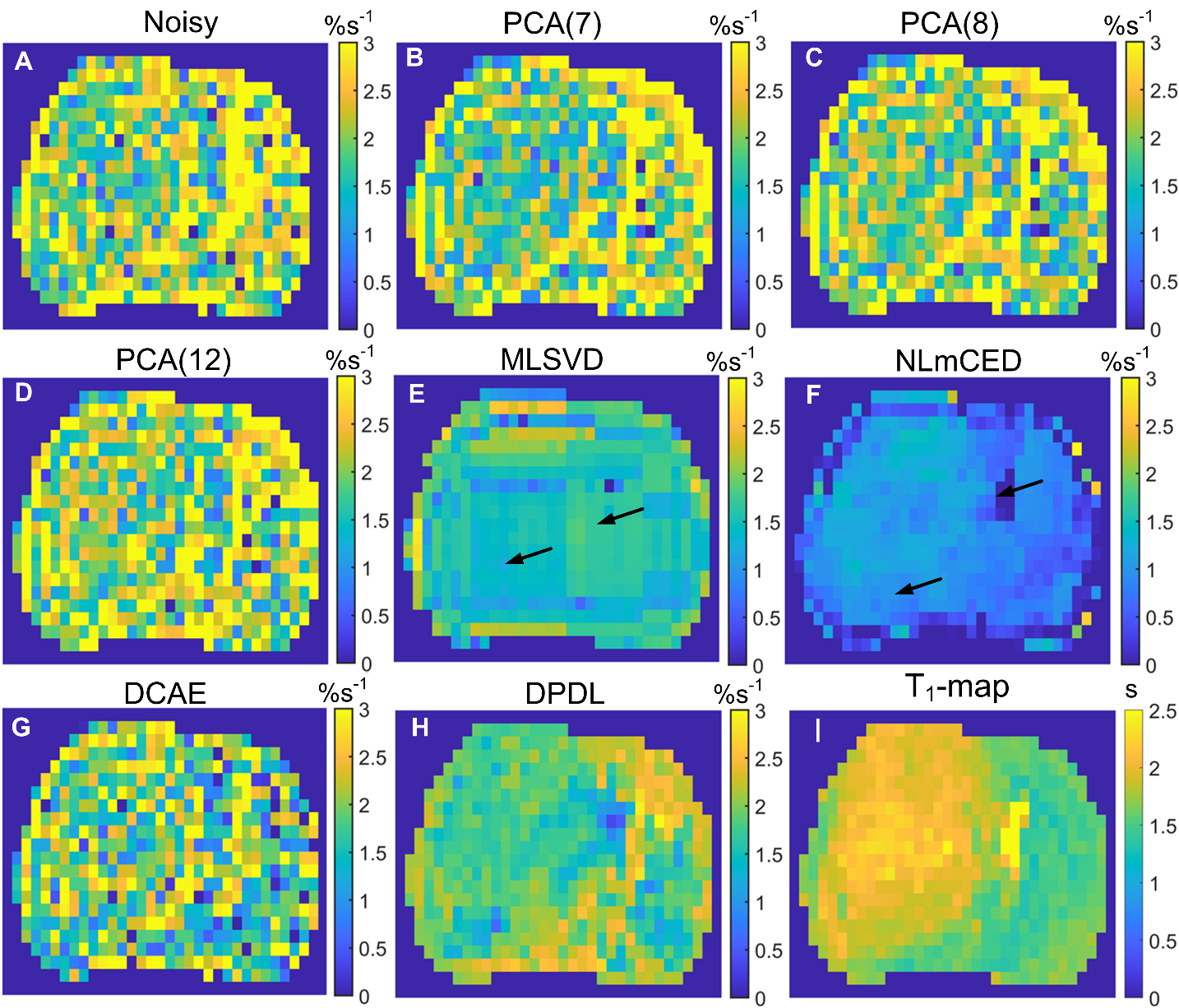


**Supporting information Fig.S18:** LD-fitted APT maps from a rat brain bearing a 9L tumor (#4), without denoising (A) and with denoising by PCA(7) (B), PCA(8) (C), PCA(12) (D), MLSVD (E), NLmCED (F), DCAE (G), and DPDL (H). T_1_ map was shown in (I) to demonstrate the tumor region. Arrows in (E) and (F) point to patches of uniform intensity, highlighting the suboptimal performance of the denoising.


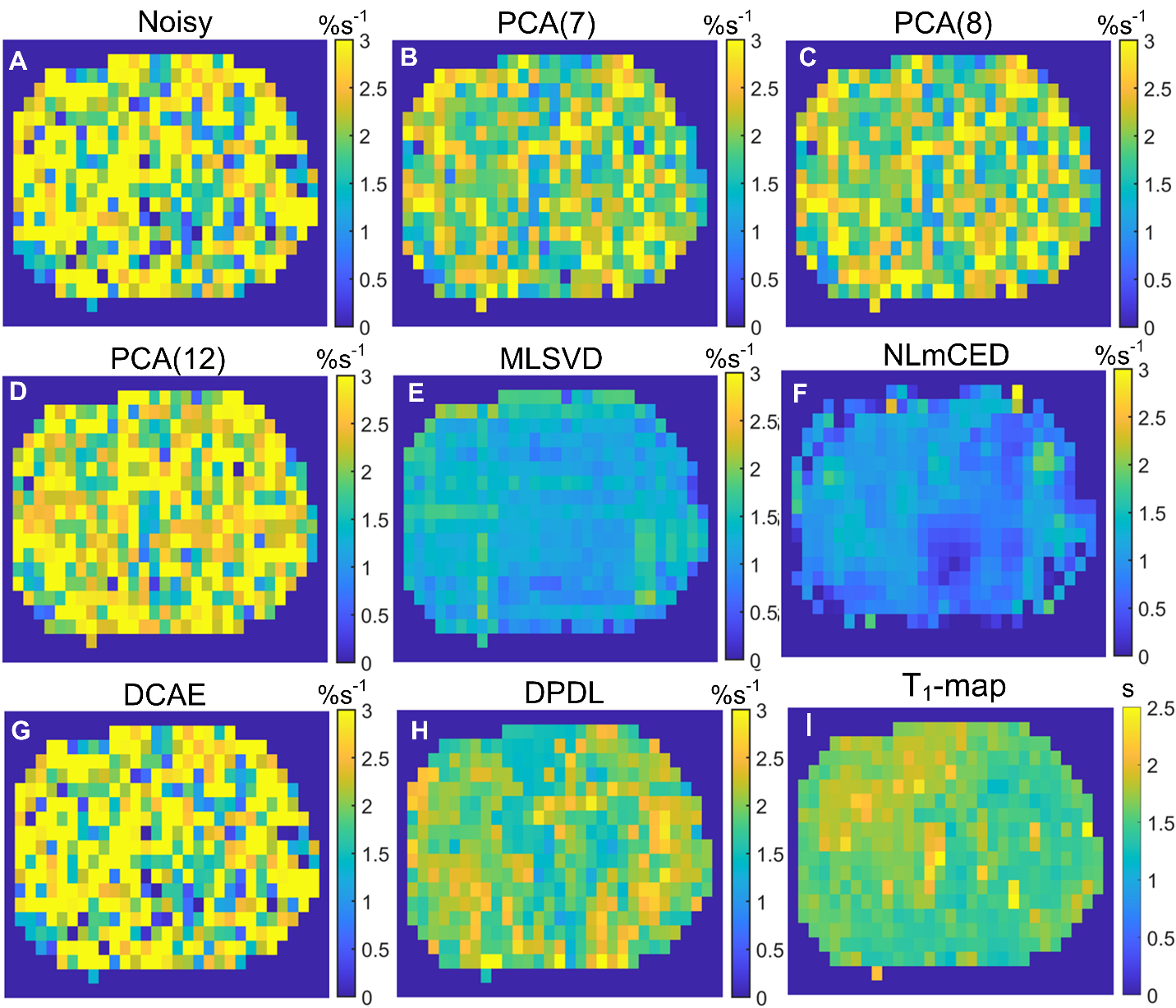


**Supporting information Fig.S19:** LD-fitted APT maps from a rat brain bearing a 9L tumor (#5), without denoising (A) and with denoising by PCA(7) (B), PCA(8) (C), PCA(12) (D), MLSVD (E), NLmCED (F), DCAE (G), and DPDL (H). T_1_ map was shown in (I) to demonstrate the tumor region.


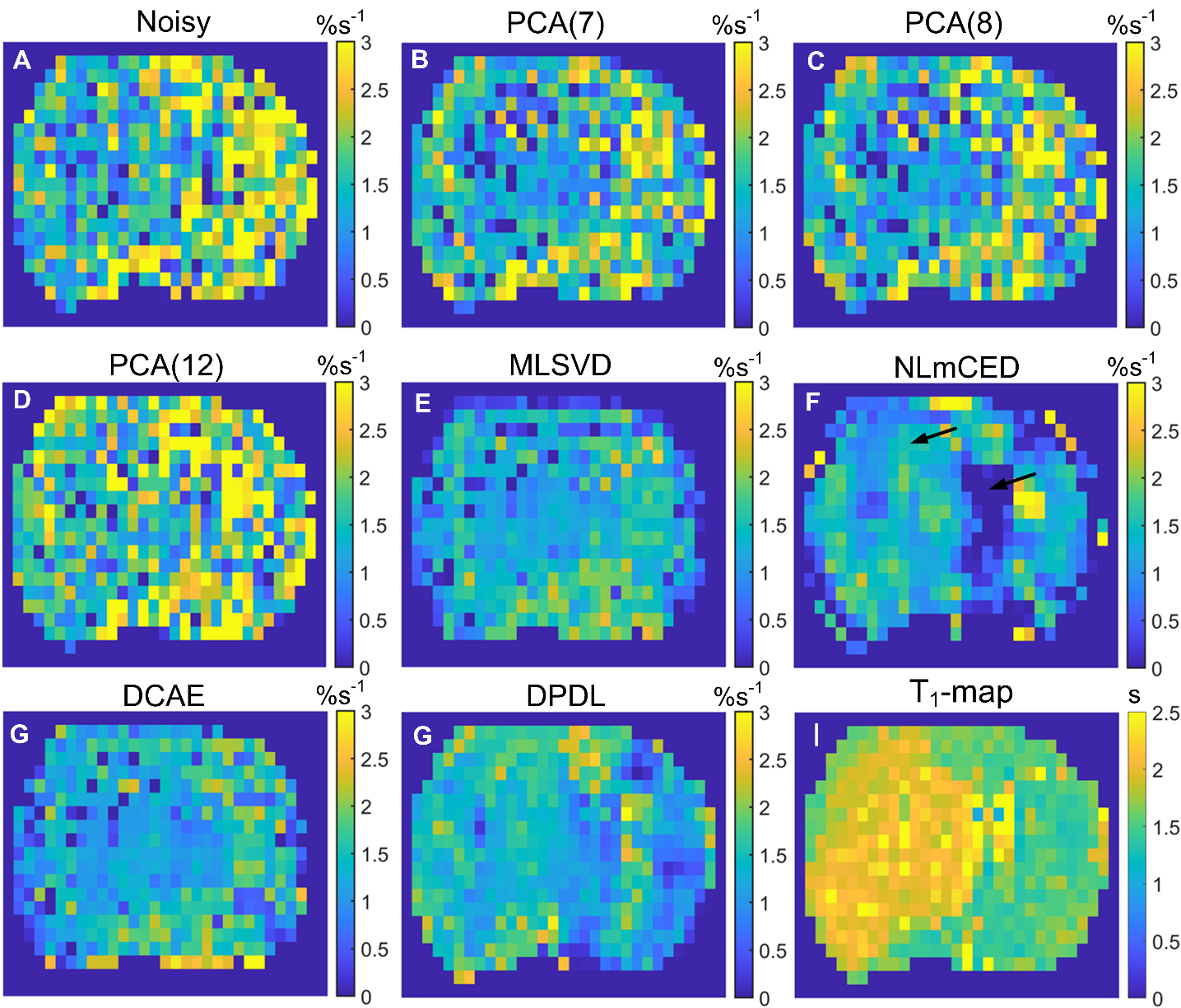


**Supporting information Fig.S20:** LD-fitted APT maps from a rat brain bearing a 9L tumor (#6), without denoising (A) and with denoising by PCA(7) (B), PCA(8) (C), PCA(12) (D), MLSVD (E), NLmCED (F), DCAE (G), and DPDL (H). T_1_ map was shown in (I) to demonstrate the tumor region. Arrows in (F) point to patches of uniform intensity, highlighting the suboptimal performance of the denoising.


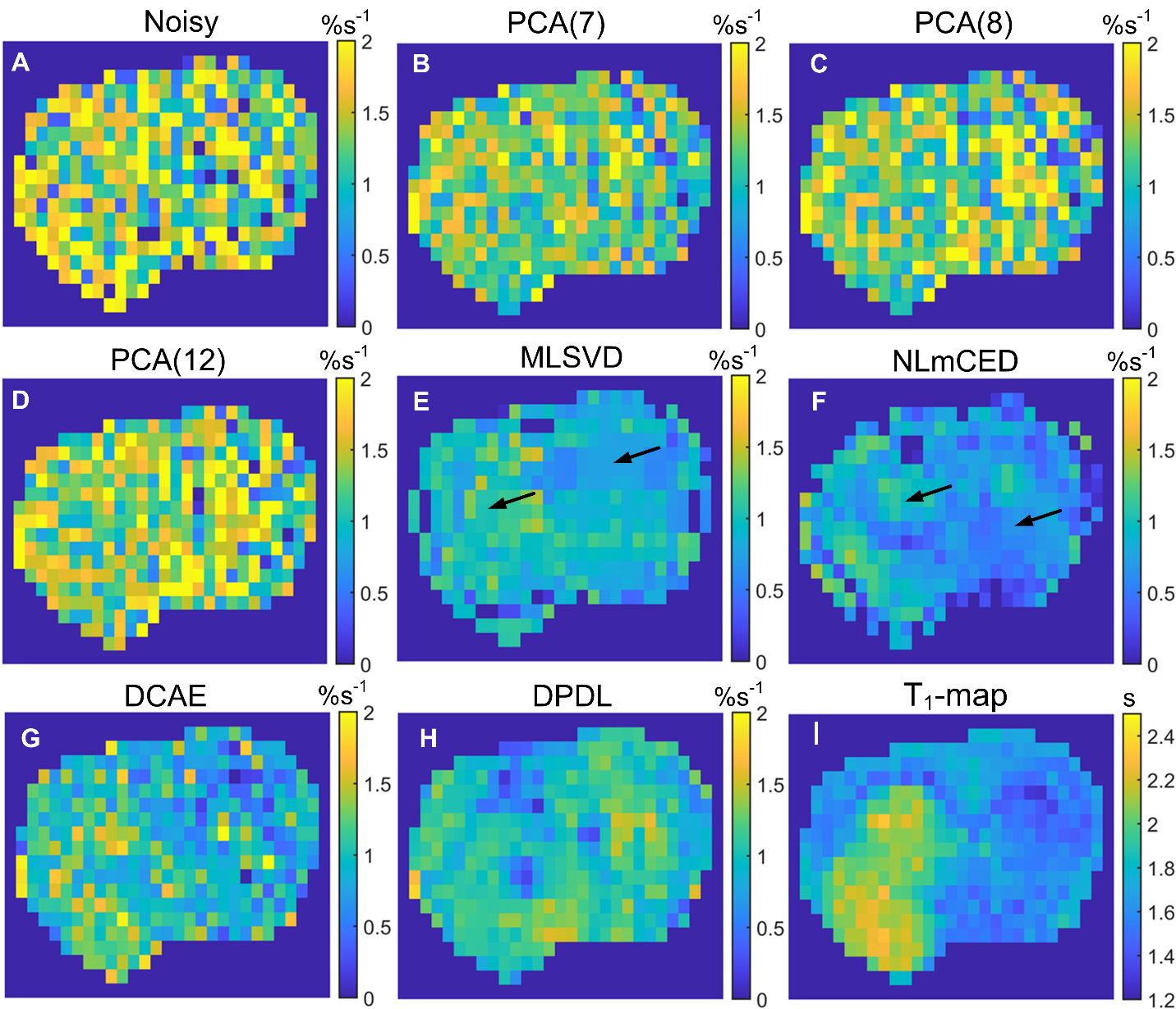


**Supporting information Fig.S21:** LD-fitted Guanidine maps from a rat brain bearing a 9L tumor (#1), without denoising (A) and with denoising by PCA(7) (B), PCA(8) (C), PCA(12) (D), MLSVD (E), NLmCED (F), DCAE (G), and DPDL (H). T_1_ map was shown in (I) to demonstrate the tumor region. Arrows in (E) and (F) point to patches of uniform intensity, highlighting the suboptimal performance of the denoising.
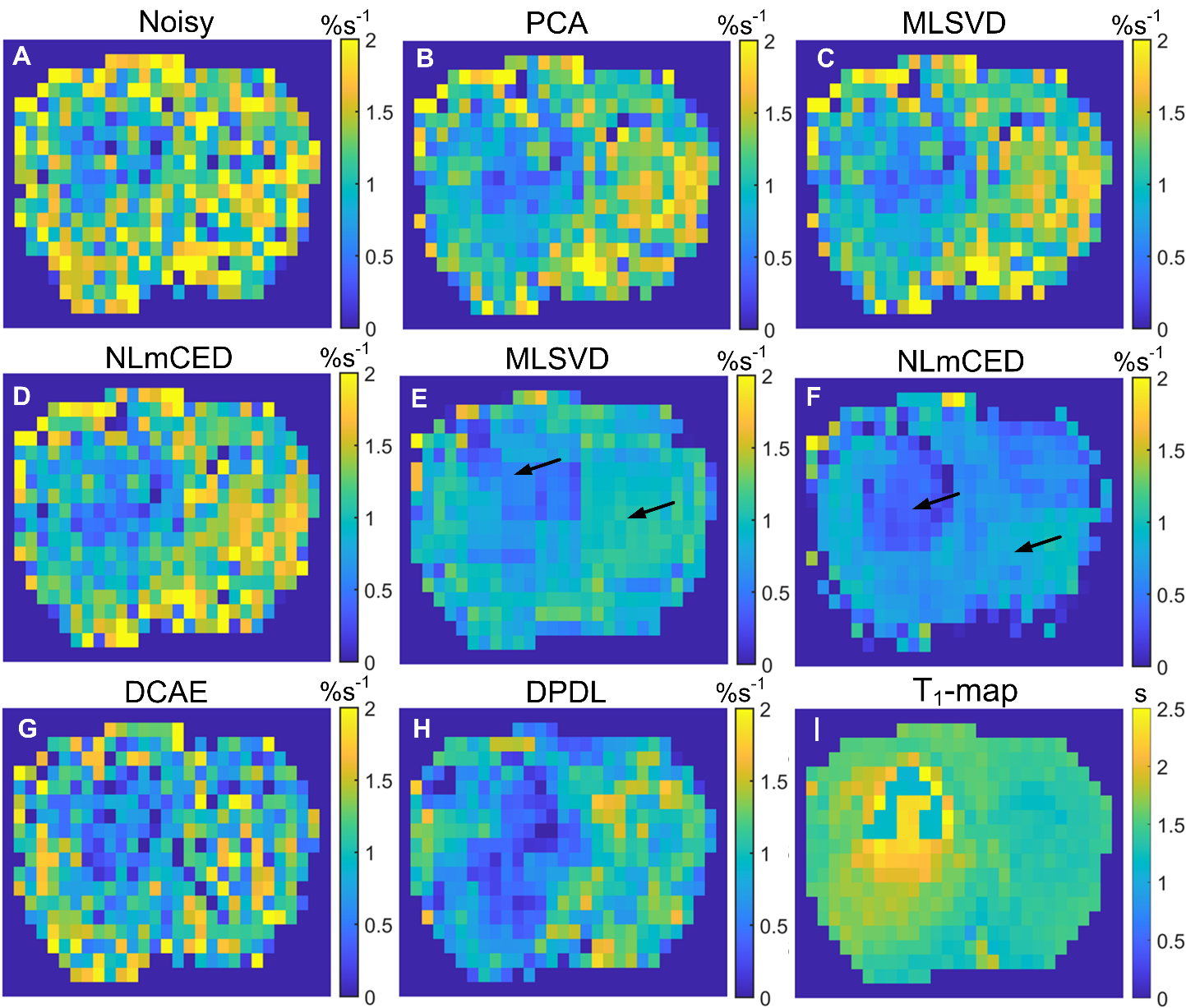


**Supporting information Fig.S22:** LD-fitted Guanidine maps from a rat brain bearing a 9L tumor (#2), without denoising (A) and with denoising by PCA(7) (B), PCA(8) (C), PCA(12) (D), MLSVD (E), NLmCED (F), DCAE (G), and DPDL (H). T_1_ map was shown in (I) to demonstrate the tumor region. Arrows in (E) and (F) point to patches of uniform intensity, highlighting the suboptimal performance of the denoising.


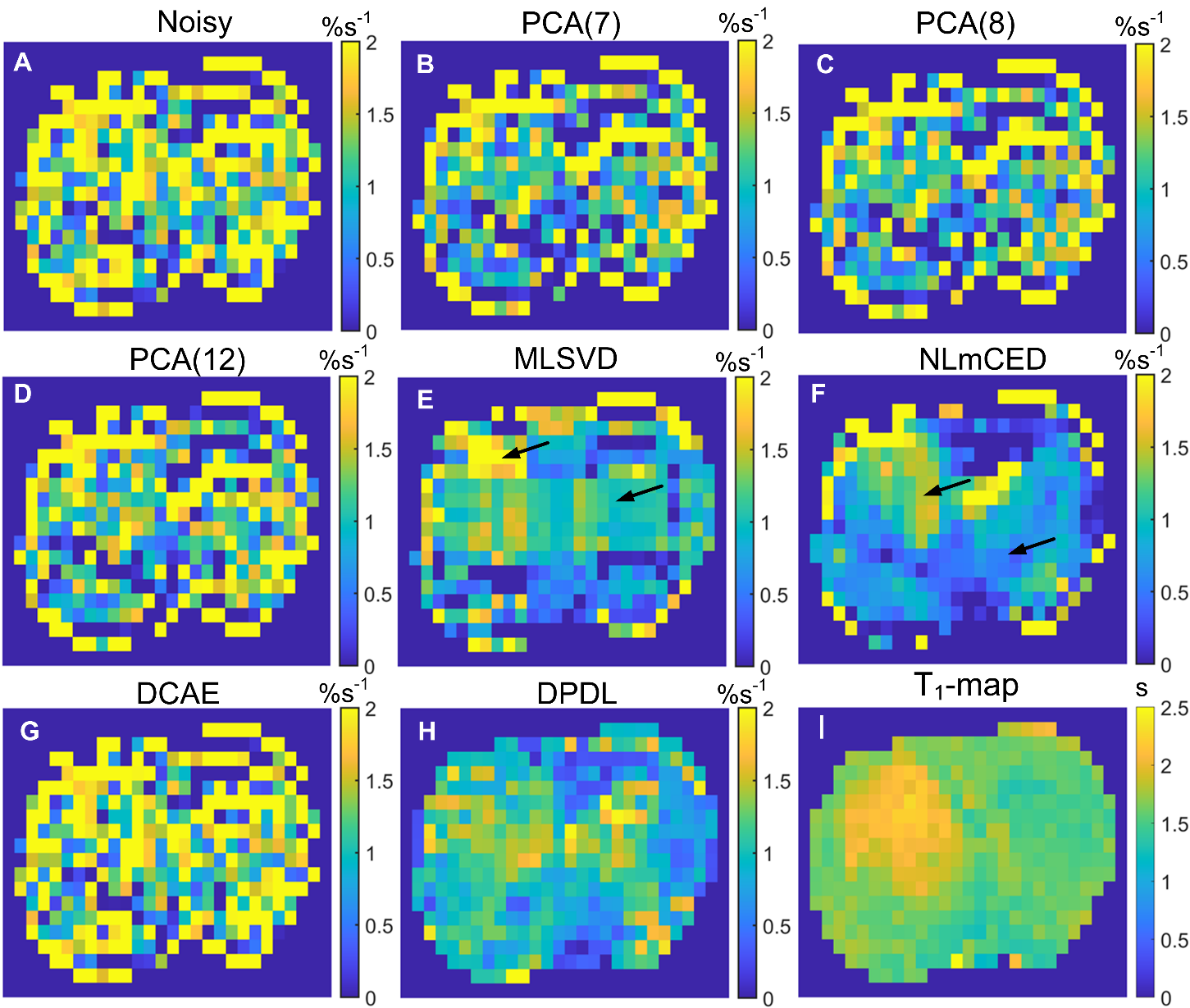


**Supporting information Fig.S23:** LD-fitted Guanidine maps from a rat brain bearing a 9L tumor (#3), without denoising (A) and with denoising by PCA(7) (B), PCA(8) (C), PCA(12) (D), MLSVD (E), NLmCED (F), DCAE (G), and DPDL (H). T_1_ map was shown in (I) to demonstrate the tumor region. Arrows in (E) and (F) point to patches of uniform intensity, highlighting the suboptimal performance of the denoising.


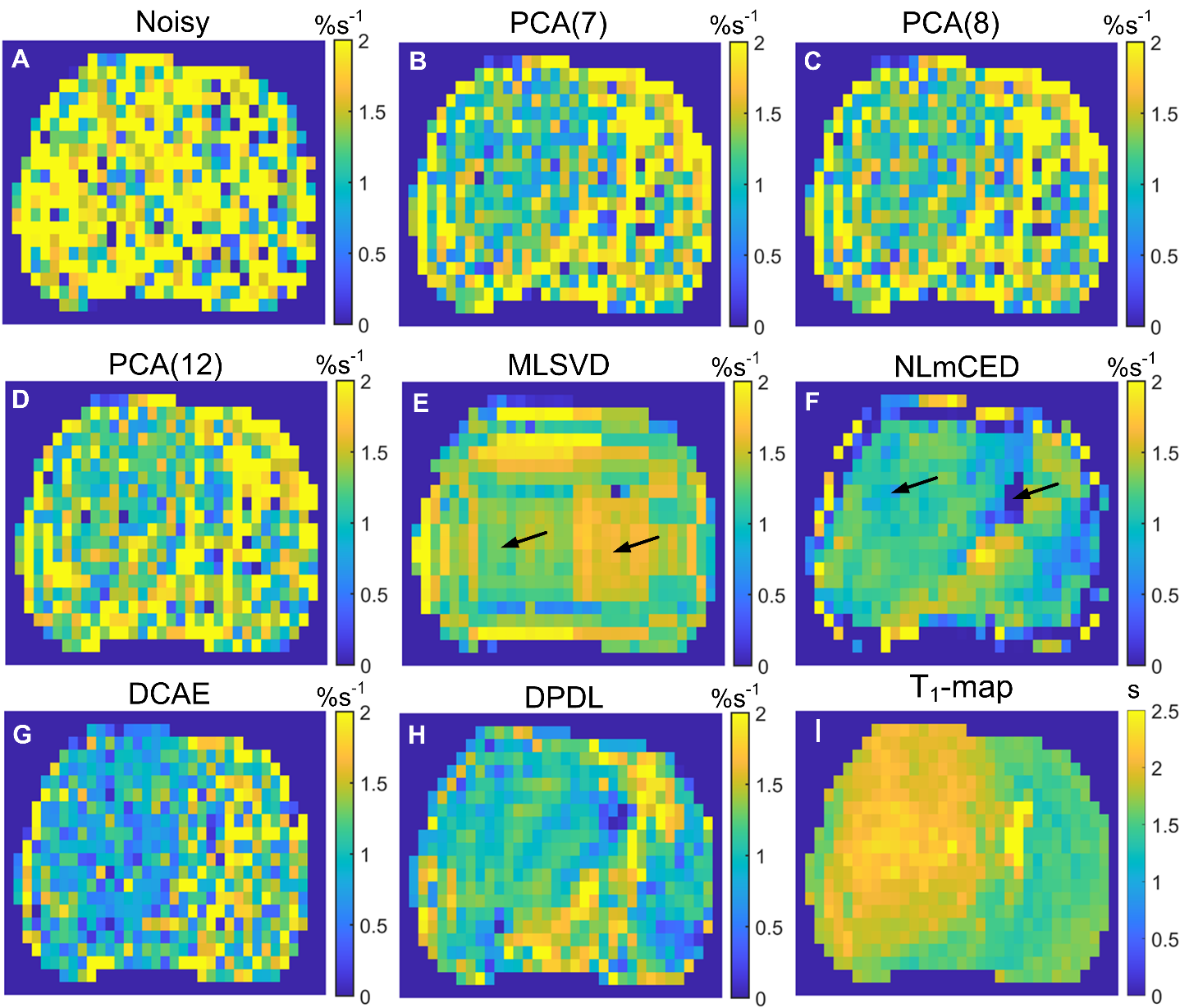


**Supporting information Fig.S24:** LD-fitted Guanidine maps from a rat brain bearing a 9L tumor (#4), without denoising (A) and with denoising by PCA(7) (B), PCA(8) (C), PCA(12) (D), MLSVD (E), NLmCED (F), DCAE (G), and DPDL (H). T_1_ map was shown in (I) to demonstrate the tumor region. Arrows in (E) and (F) point to patches of uniform intensity, highlighting the suboptimal performance of the denoising.


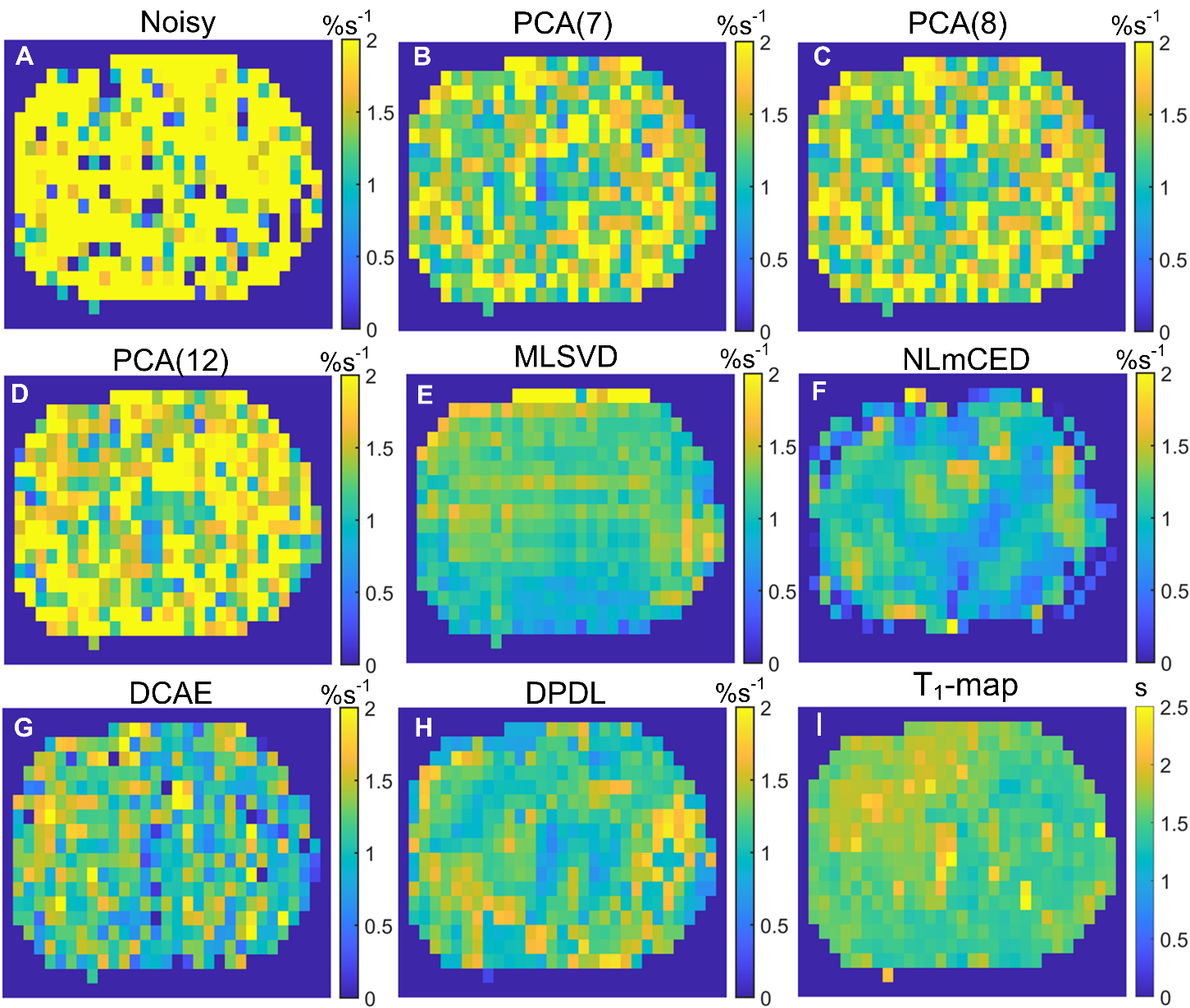


**Supporting information Fig.S25:** LD-fitted Guanidine maps from a rat brain bearing a 9L tumor (#5), without denoising (A) and with denoising by PCA(7) (B), PCA(8) (C), PCA(12) (D), MLSVD (E), NLmCED (F), DCAE (G), and DPDL (H). T_1_ map was shown in (I) to demonstrate the tumor region.


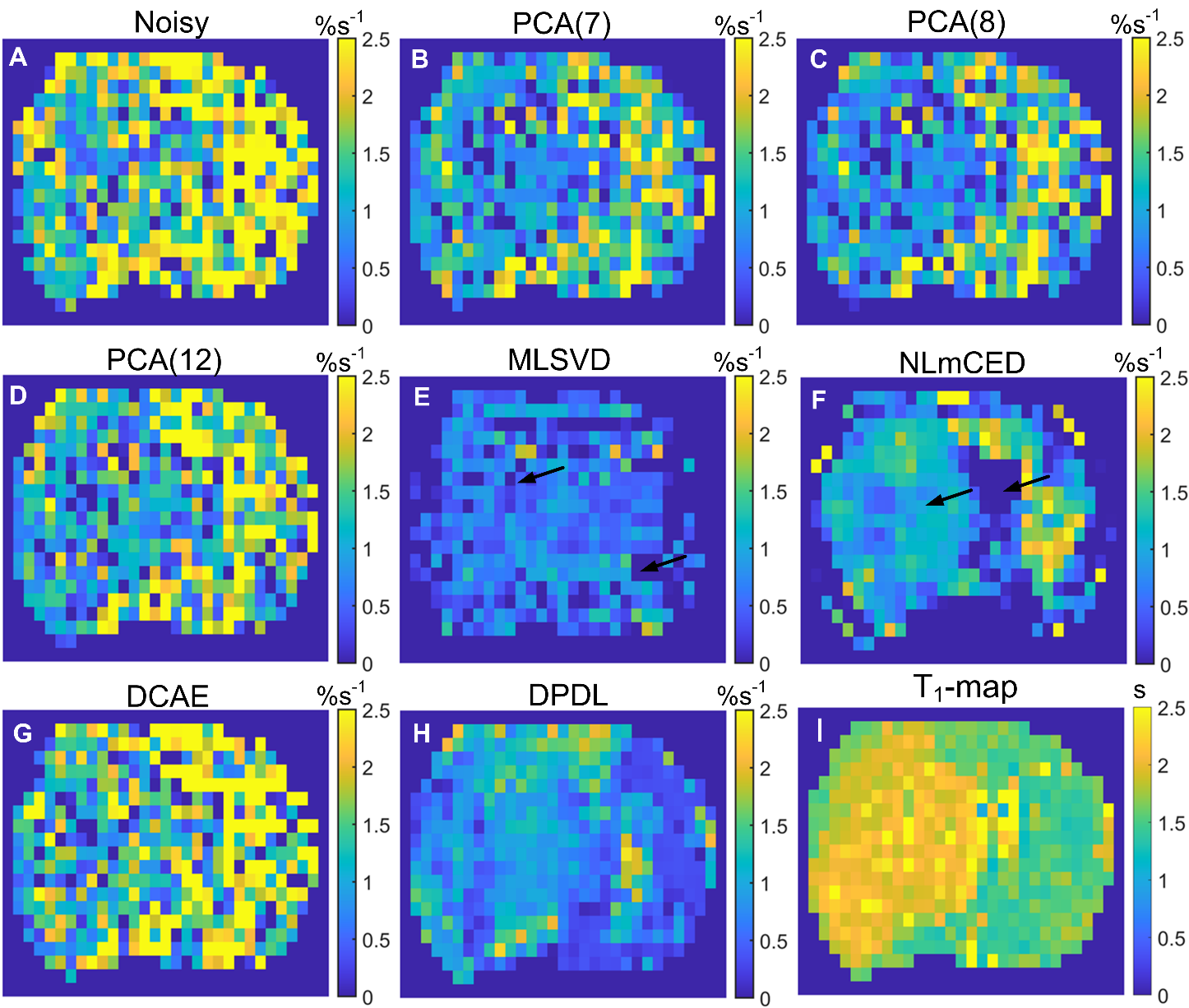


**Supporting information Fig.S26:** LD-fitted Guanidine maps from a rat brain bearing a 9L tumor (#6), without denoising (A) and with denoising by PCA(7) (B), PCA(8) (C), PCA(12) (D), MLSVD (E), NLmCED (F), DCAE (G), and DPDL (H). T_1_ map was shown in (I) to demonstrate the tumor region. Arrows in (E) and (F) point to patches of uniform intensity, highlighting the suboptimal performance of the denoising.


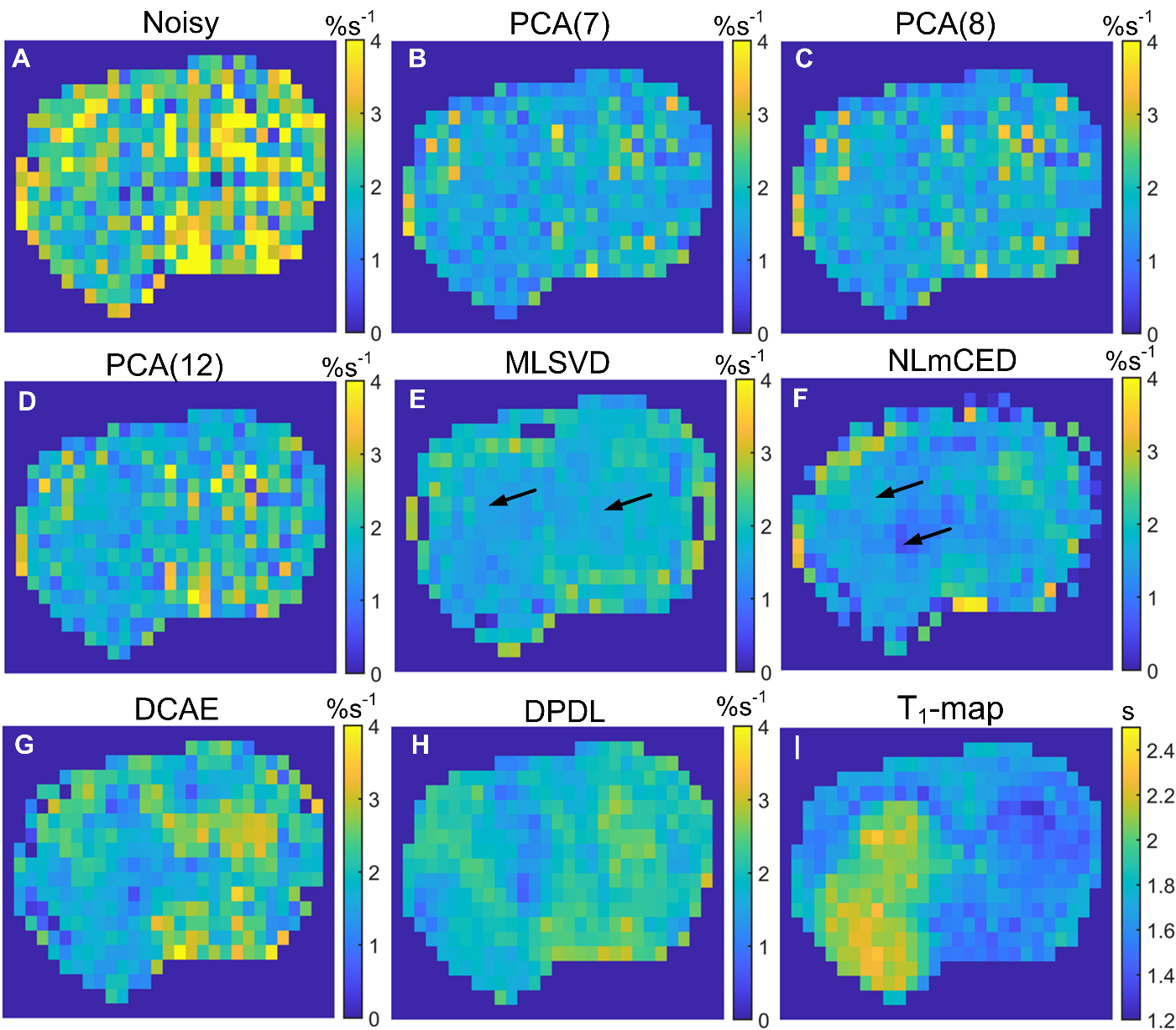


**Supporting information Fig.S27:** LD-fitted NOE(-1.6) maps from a rat brain bearing a 9L tumor (#1), without denoising (A) and with denoising by PCA(7) (B), PCA(8) (C), PCA(12) (D), MLSVD (E), NLmCED (F), DCAE (G), and DPDL (H). T_1_ map was shown in (I) to demonstrate the tumor region. Arrows in (E) and (F) point to patches of uniform intensity, highlighting the suboptimal performance of the denoising.


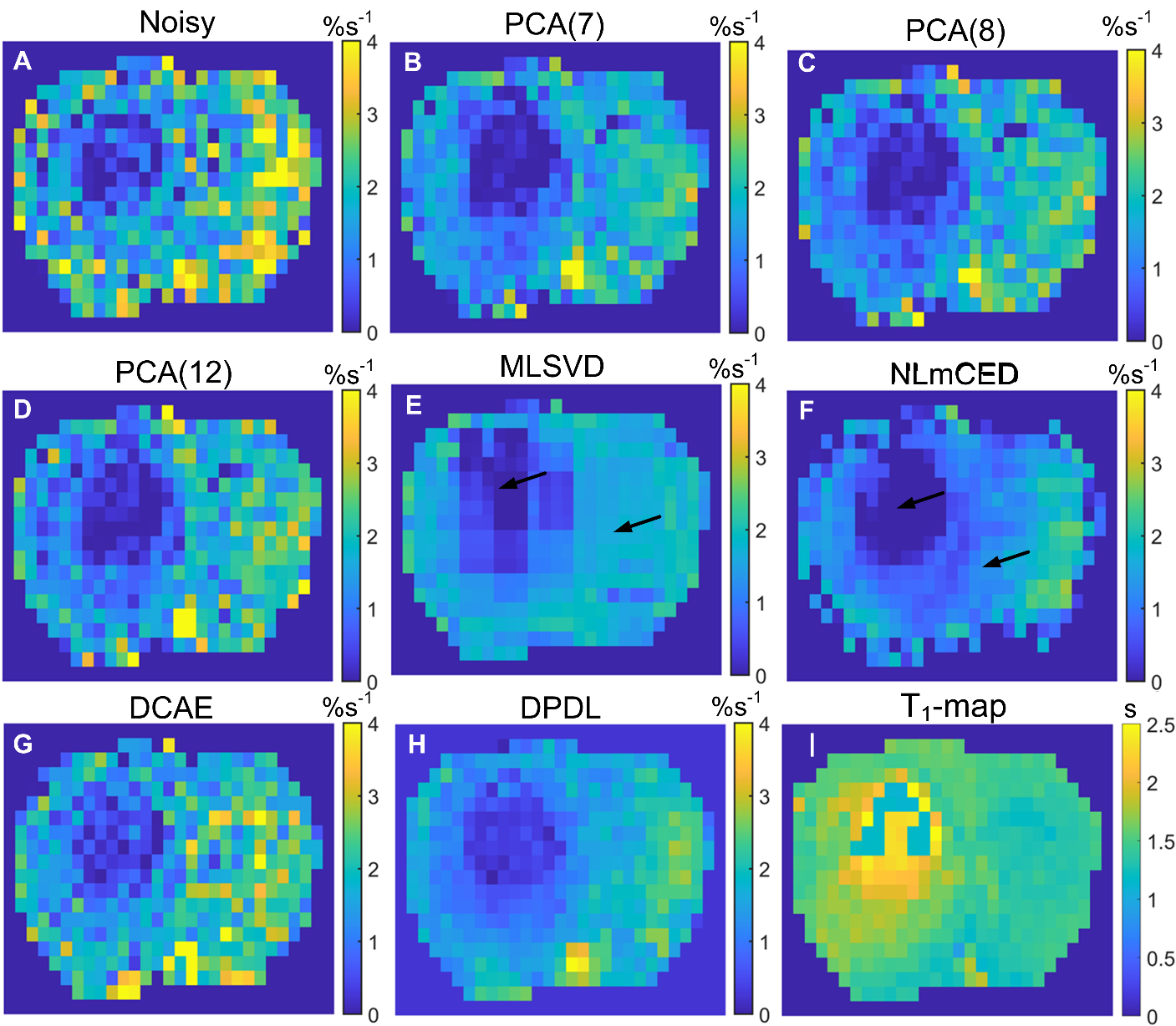


**Supporting information Fig.S28:** LD-fitted NOE(-1.6) maps from a rat brain bearing a 9L tumor (#2), without denoising (A) and with denoising by PCA(7) (B), PCA(8) (C), PCA(12) (D), MLSVD (E), NLmCED (F), DCAE (G), and DPDL (H). T_1_ map was shown in (I) to demonstrate the tumor region. Arrows in (E) and (F) point to patches of uniform intensity, highlighting the suboptimal performance of the denoising.


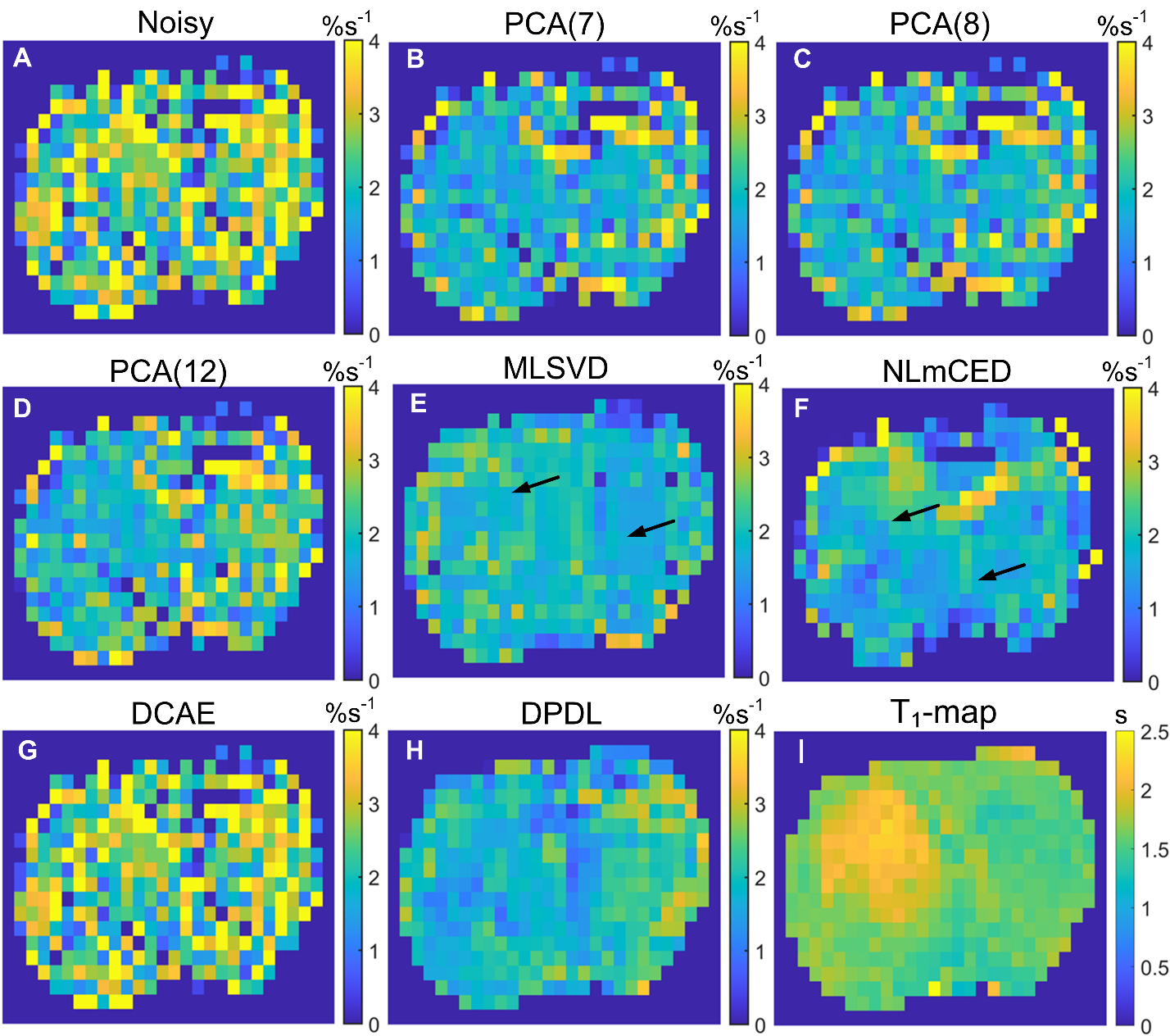


**Supporting information Fig.S29:** LD-fitted NOE(-1.6) maps from a rat brain bearing a 9L tumor (#3), without denoising (A) and with denoising by PCA(7) (B), PCA(8) (C), PCA(12) (D), MLSVD (E), NLmCED (F), DCAE (G), and DPDL (H). T_1_ map was shown in (I) to demonstrate the tumor region. Arrows in (E) and (F) point to patches of uniform intensity, highlighting the suboptimal performance of the denoising.


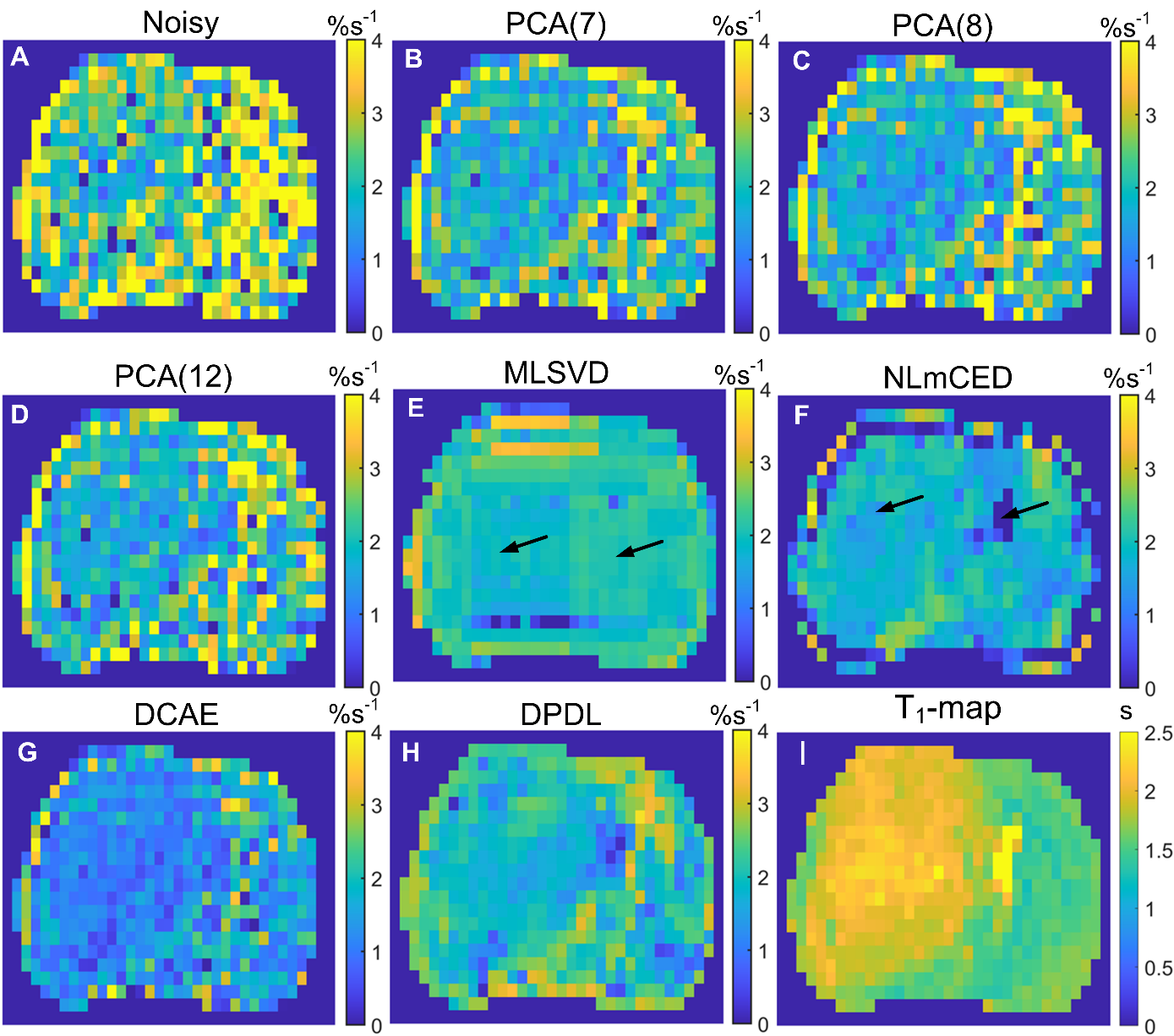


**Supporting information Fig.S30:** LD-fitted NOE(-1.6) maps from a rat brain bearing a 9L tumor (#4), without denoising (A) and with denoising by PCA(7) (B), PCA(8) (C), PCA(12) (D), MLSVD (E), NLmCED (F), DCAE (G), and DPDL (H). T_1_ map was shown in (I) to demonstrate the tumor region. Arrows in (E) and (F) point to patches of uniform intensity, highlighting the suboptimal performance of the denoising.


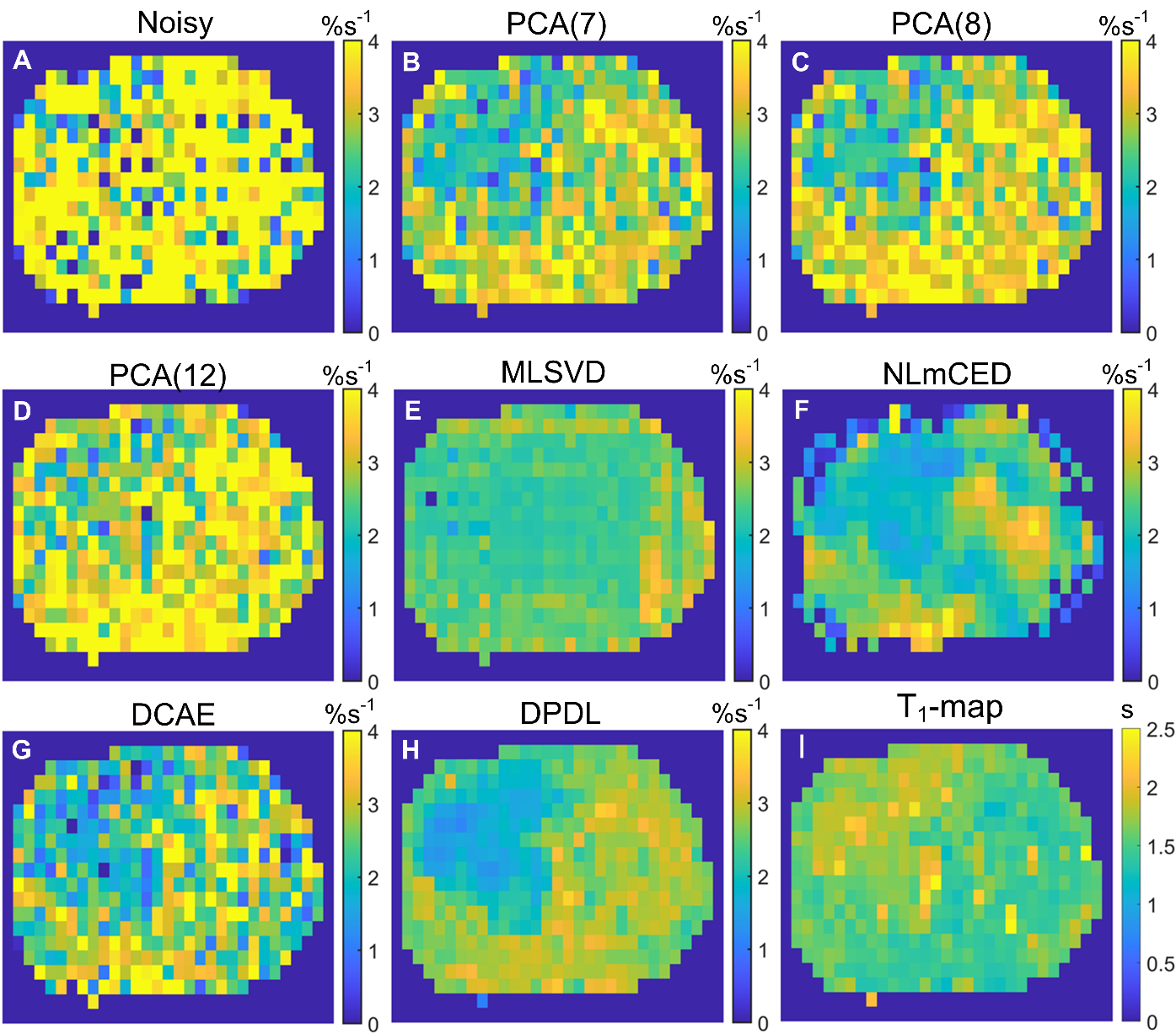


**Supporting information Fig.S31:** LD-fitted NOE(-1.6) maps from a rat brain bearing a 9L tumor (#5), without denoising (A) and with denoising by PCA(7) (B), PCA(8) (C), PCA(12) (D), MLSVD (E), NLmCED (F), DCAE (G), and DPDL (H). T_1_ map was shown in (I) to demonstrate the tumor region.


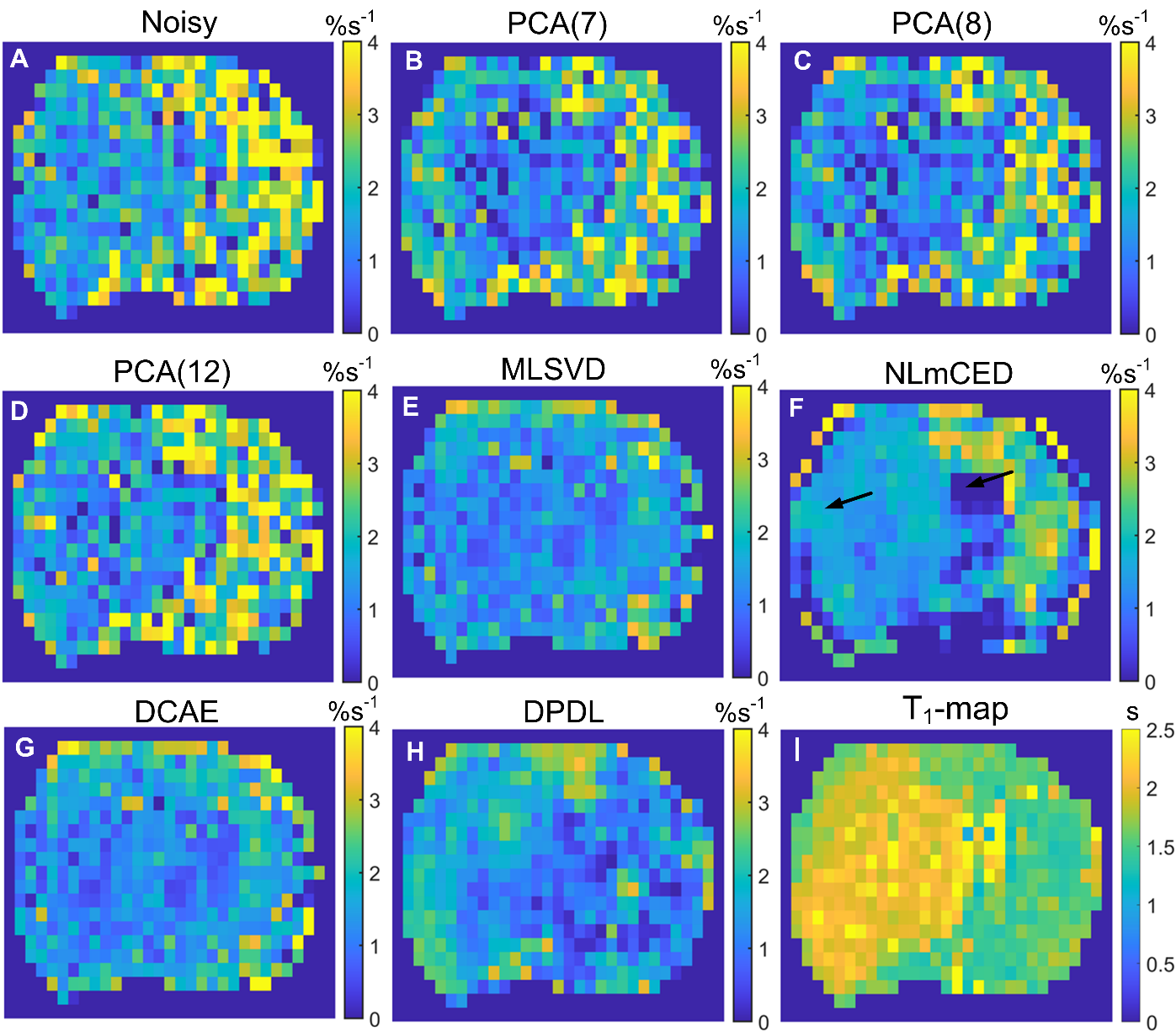


**Supporting information Fig.S32:** LD-fitted NOE(-1.6) maps from a rat brain bearing a 9L tumor (#6), without denoising (A) and with denoising by PCA(7) (B), PCA(8) (C), PCA(12) (D), MLSVD (E), NLmCED (F), DCAE (G), and DPDL (H). T_1_ map was shown in (I) to demonstrate the tumor region. Arrows in (F) point to patches of uniform intensity, highlighting the suboptimal performance of the denoising.


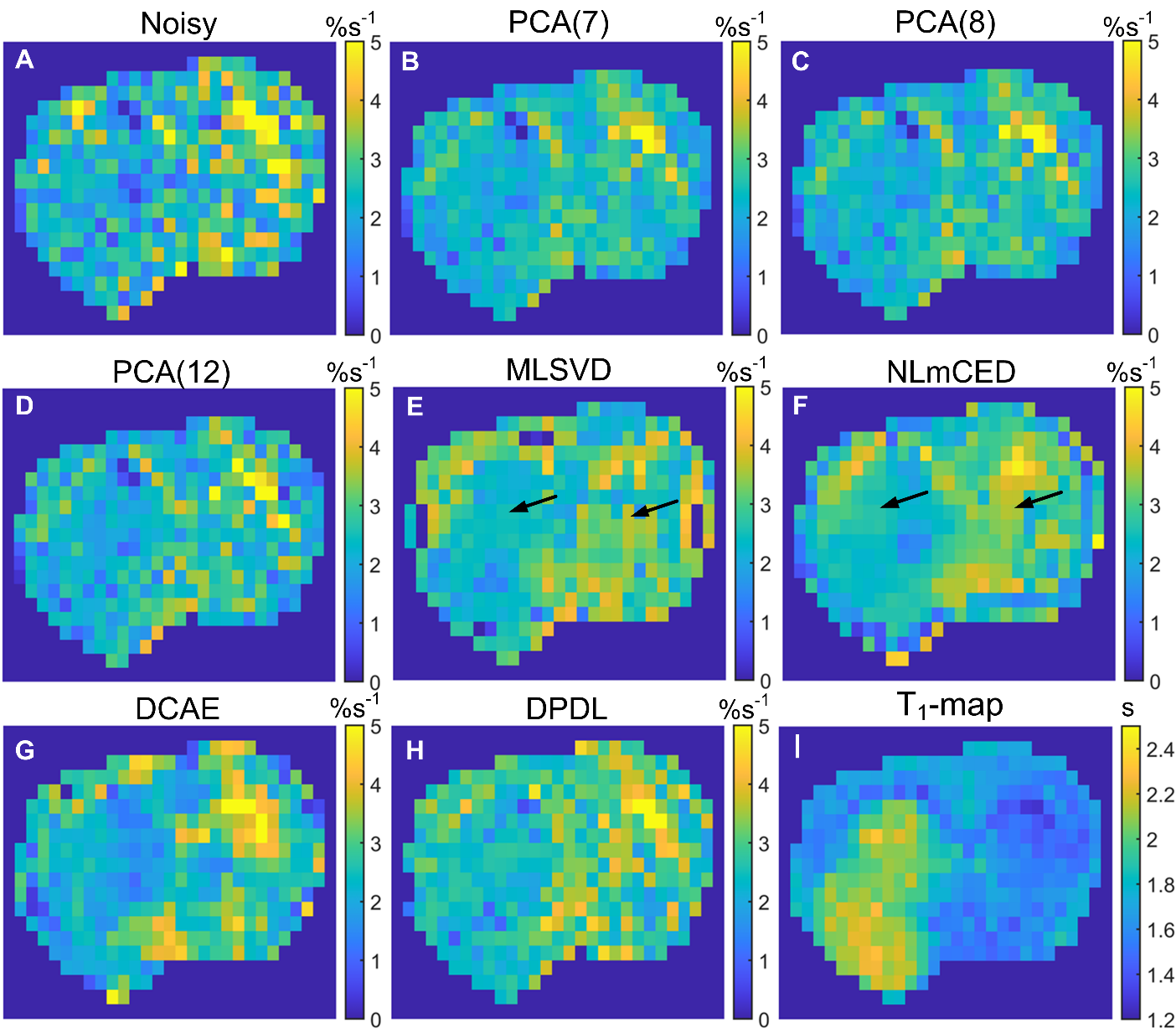


**Supporting information Fig.S33:** LD-fitted NOE(-3.5) maps from a rat brain bearing a 9L tumor (#1), without denoising (A) and with denoising by PCA(7) (B), PCA(8) (C), PCA(12) (D), MLSVD (E), NLmCED (F), DCAE (G), and DPDL (H). T_1_ map was shown in (I) to demonstrate the tumor region. Arrows in (E) and (F) point to patches of uniform intensity, highlighting the suboptimal performance of the denoising.
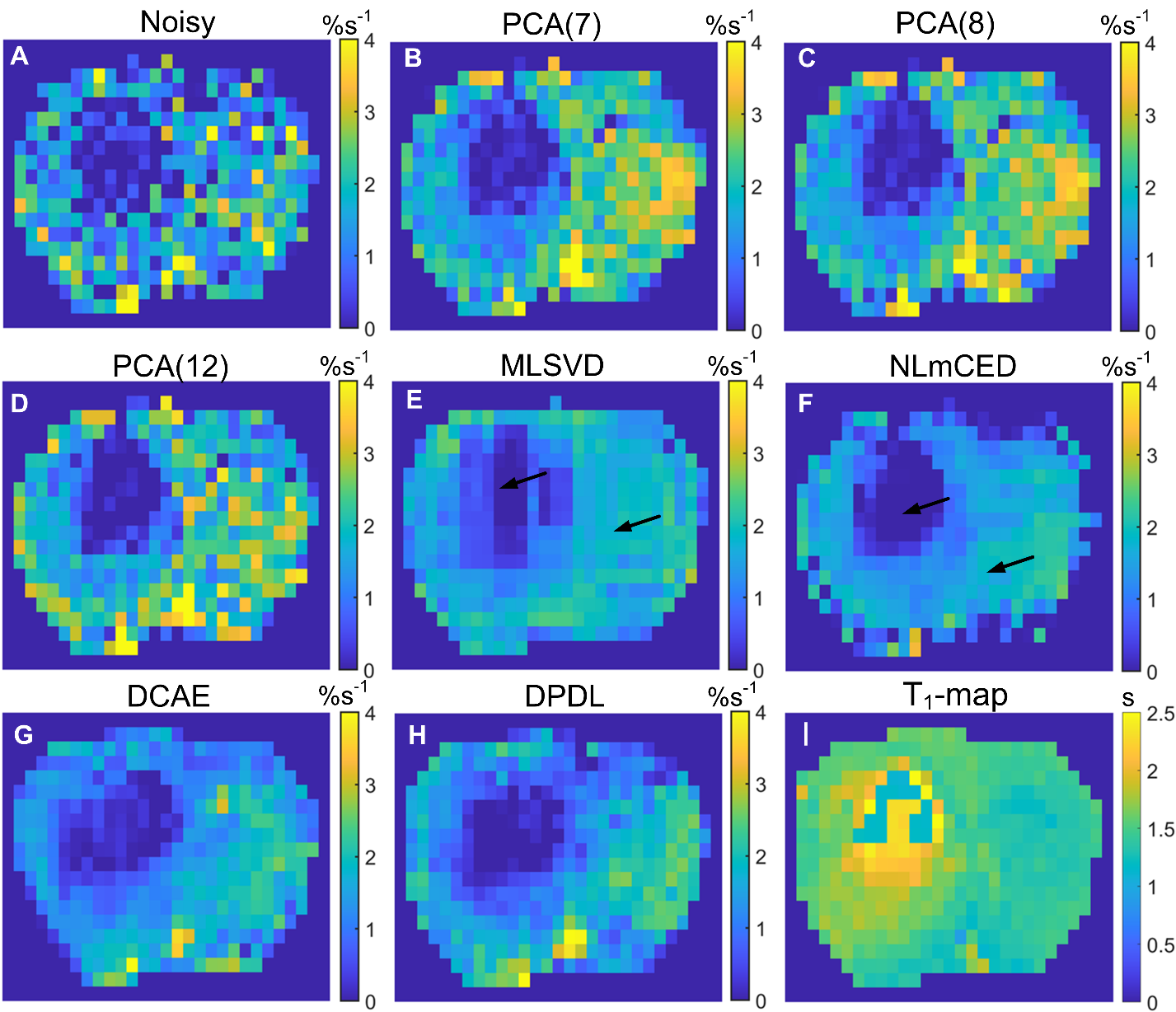


**Supporting information Fig.S34:** LD-fitted NOE(-3.5) maps from a rat brain bearing a 9L tumor (#2), without denoising (A) and with denoising by PCA(7) (B), PCA(8) (C), PCA(12) (D), MLSVD (E), NLmCED (F), DCAE (G), and DPDL (H). T_1_ map was shown in (I) to demonstrate the tumor region. Arrows in (E) and (F) point to patches of uniform intensity, highlighting the suboptimal performance of the denoising.


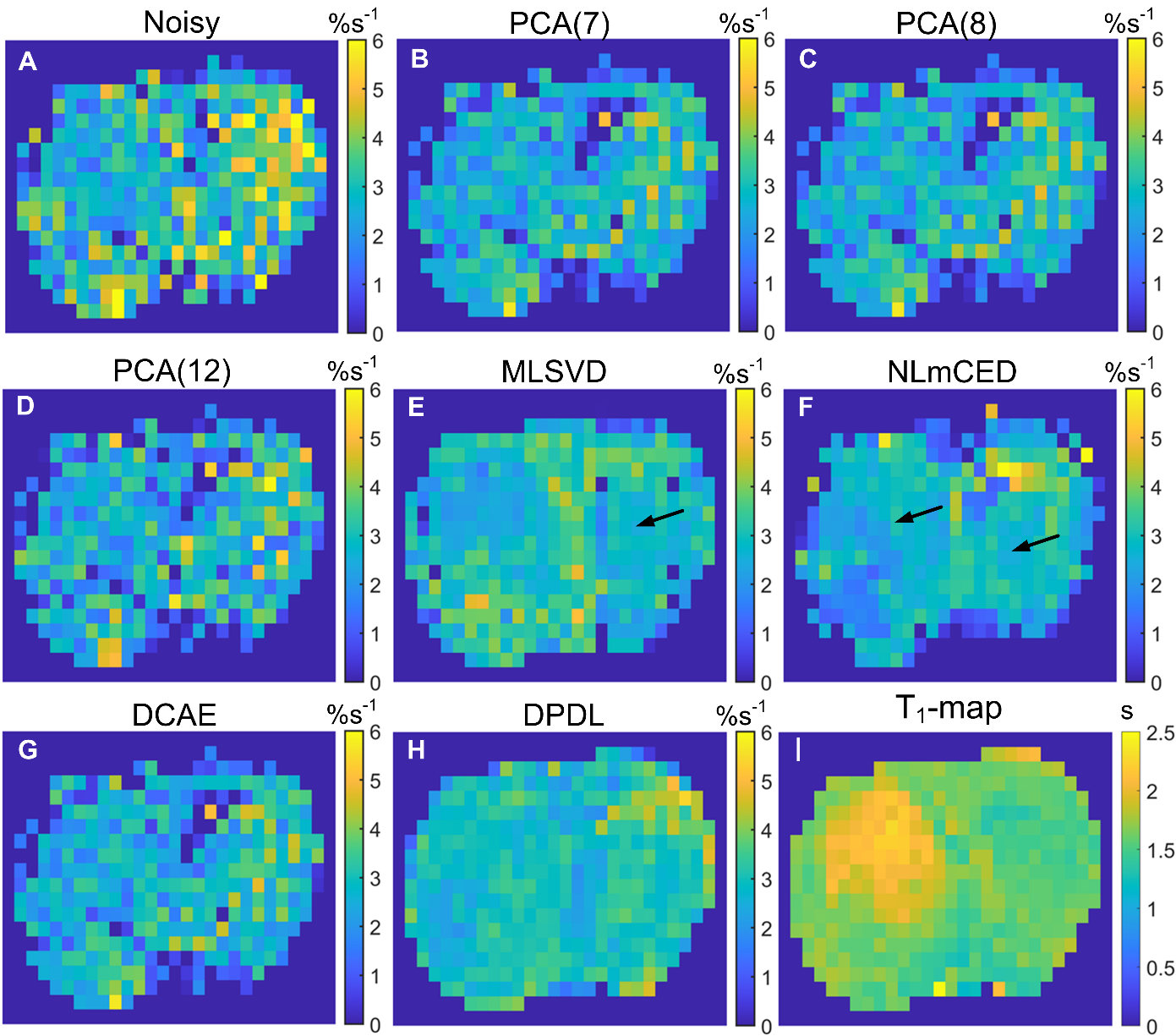


**Supporting information Fig.S35:** LD-fitted NOE(-3.5) maps from a rat brain bearing a 9L tumor (#3), without denoising (A) and with denoising by PCA(7) (B), PCA(8) (C), PCA(12) (D), MLSVD (E), NLmCED (F), DCAE (G), and DPDL (H). T_1_ map was shown in (I) to demonstrate the tumor region. Arrows in (E) and (F) point to patches of uniform intensity, highlighting the suboptimal performance of the denoising.


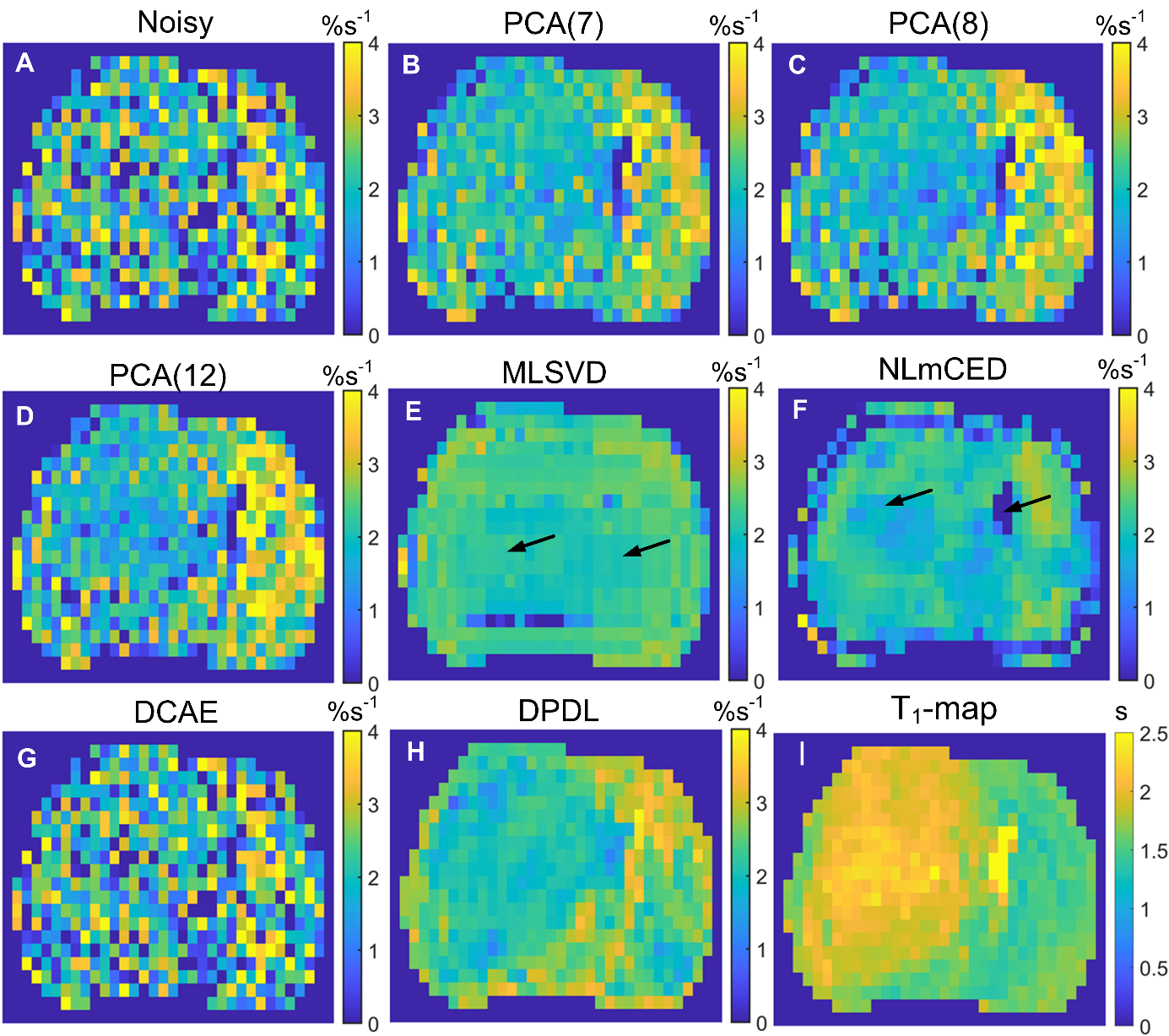


**Supporting information Fig.S36:** LD-fitted NOE(-3.5) maps from a rat brain bearing a 9L tumor (#4), without denoising (A) and with denoising by PCA(7) (B), PCA(8) (C), PCA(12) (D), MLSVD (E), NLmCED (F), DCAE (G), and DPDL (H). T_1_ map was shown in (I) to demonstrate the tumor region. Arrows in (E) and (F) point to patches of uniform intensity, highlighting the suboptimal performance of the denoising.


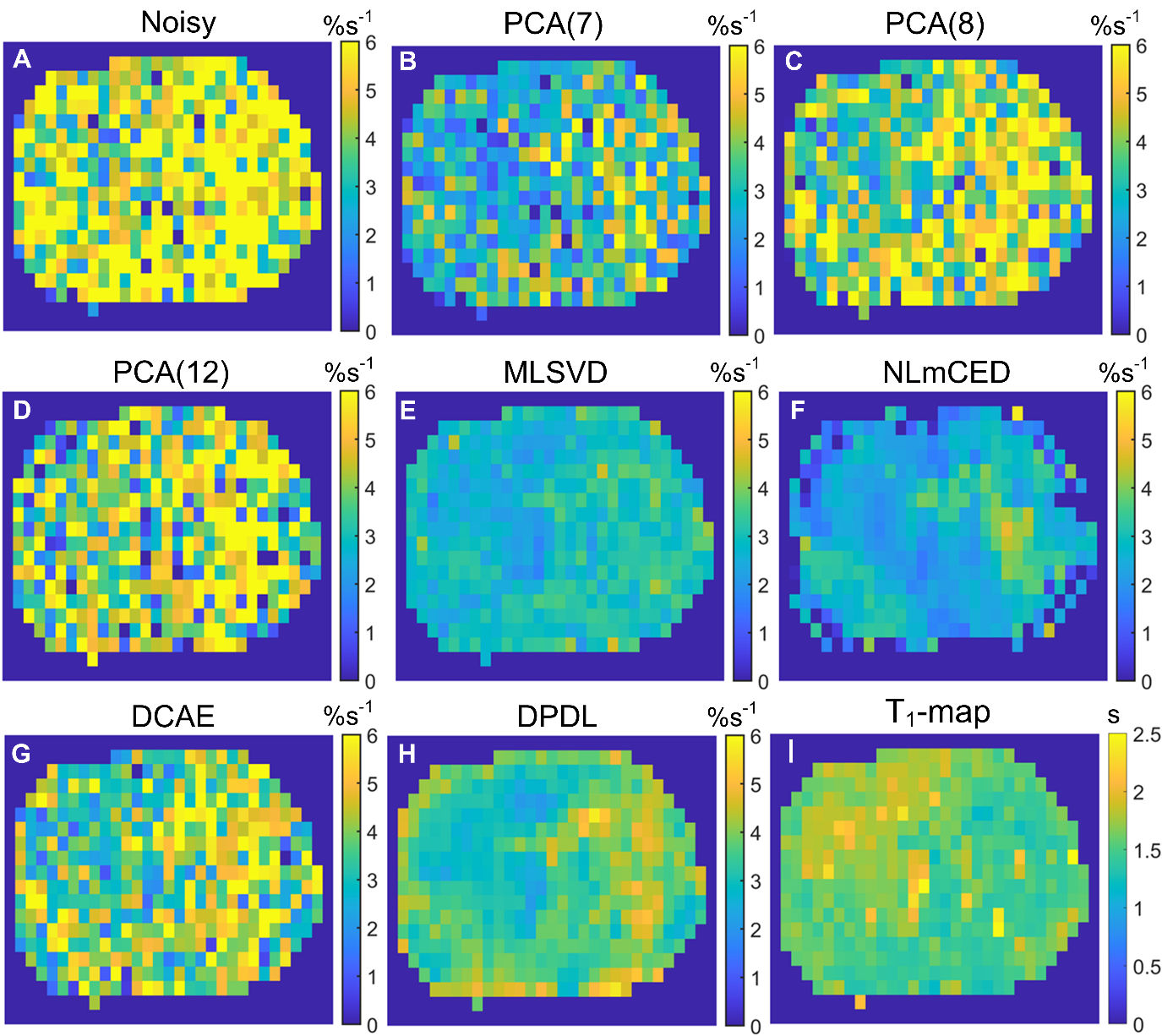


**Supporting information Fig.S37:** LD-fitted NOE(-3.5) maps from a rat brain bearing a 9L tumor (#5), without denoising (A) and with denoising by PCA(7) (B), PCA(8) (C), PCA(12) (D), MLSVD (E), NLmCED (F), DCAE (G), and DPDL (H). T_1_ map was shown in (I) to demonstrate the tumor region.


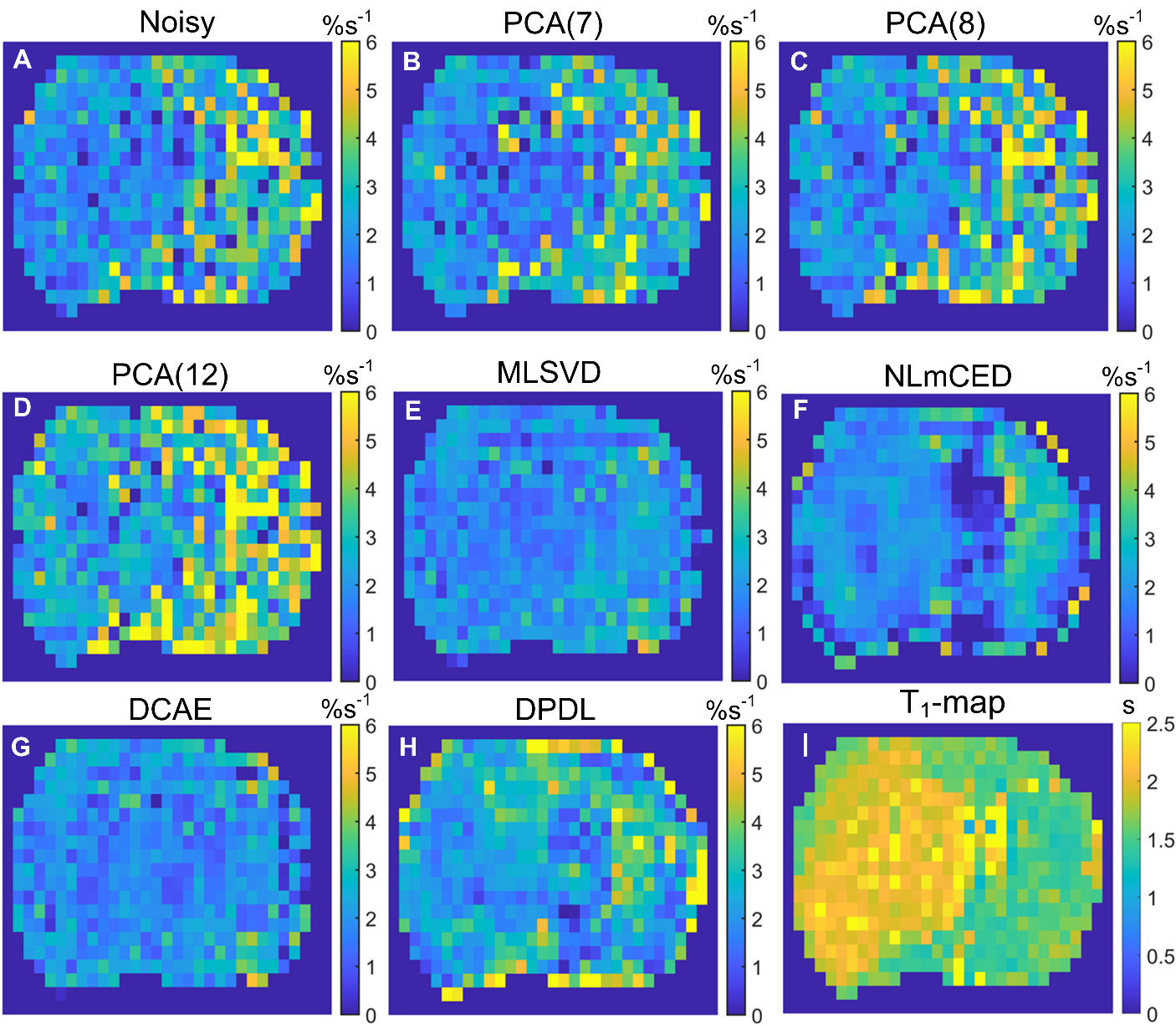


**Supporting information Fig.S38:** LD-fitted NOE(-3.5) maps from a rat brain bearing a 9L tumor (#6), without denoising (A) and with denoising by PCA(7) (B), PCA(8) (C), PCA(12) (D), MLSVD (E), NLmCED (F), DCAE (G), and DPDL (H). T_1_ map was shown in (I) to demonstrate the tumor region.


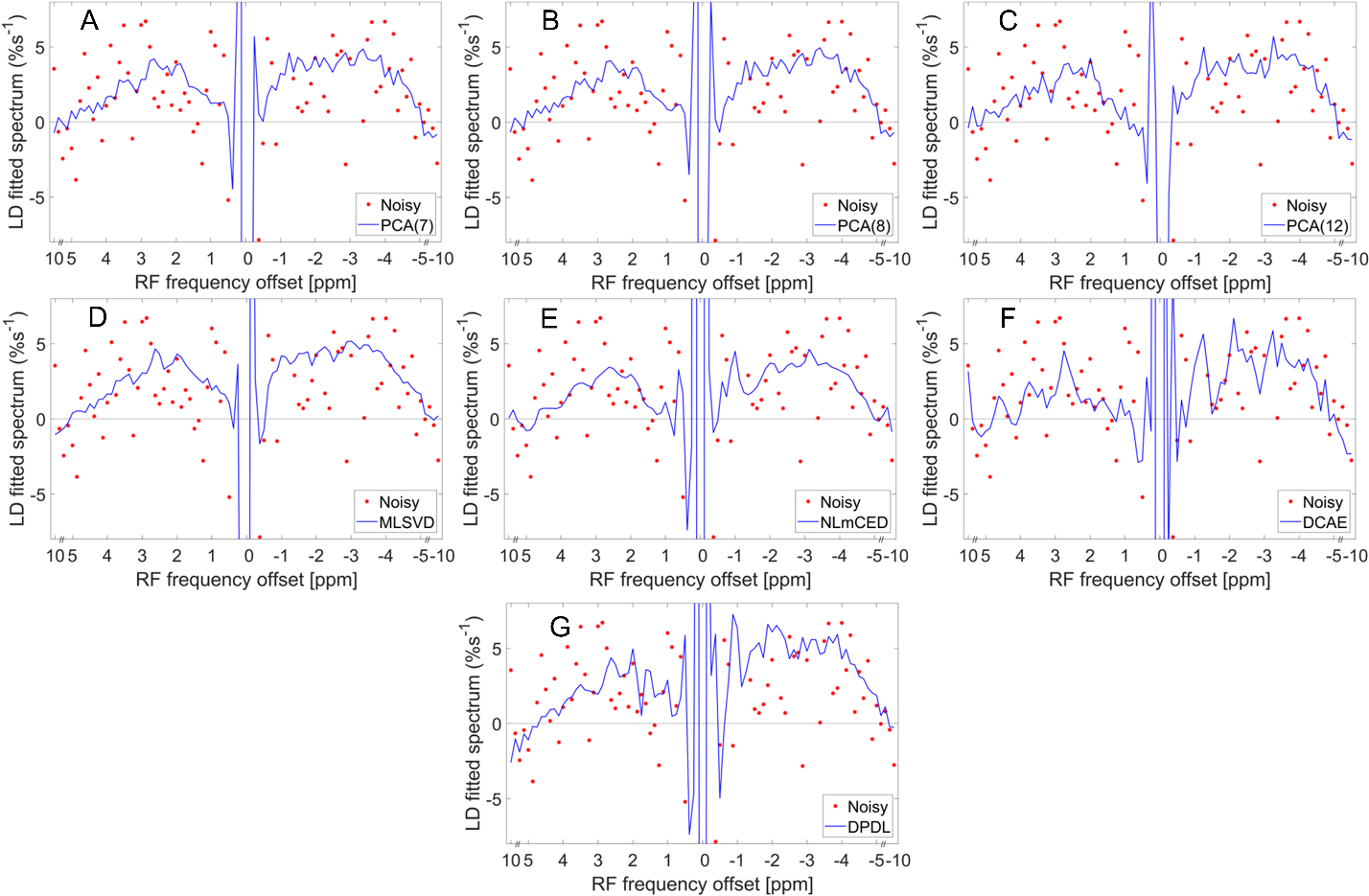


**Supporting information Fig.S39:** A sample LP LD-fitted spectrum from a single voxel in muscle in a representative healthy rat leg (#1), after denoising using various methods: PCA(7) (A), PCA(8) (B), PCA(12) (C), MLSVD (D), NLmCED (E), DCAE (F), and DPDL (G). The noisy LP LD-fitted spectrum is also included in these figures for comparison.


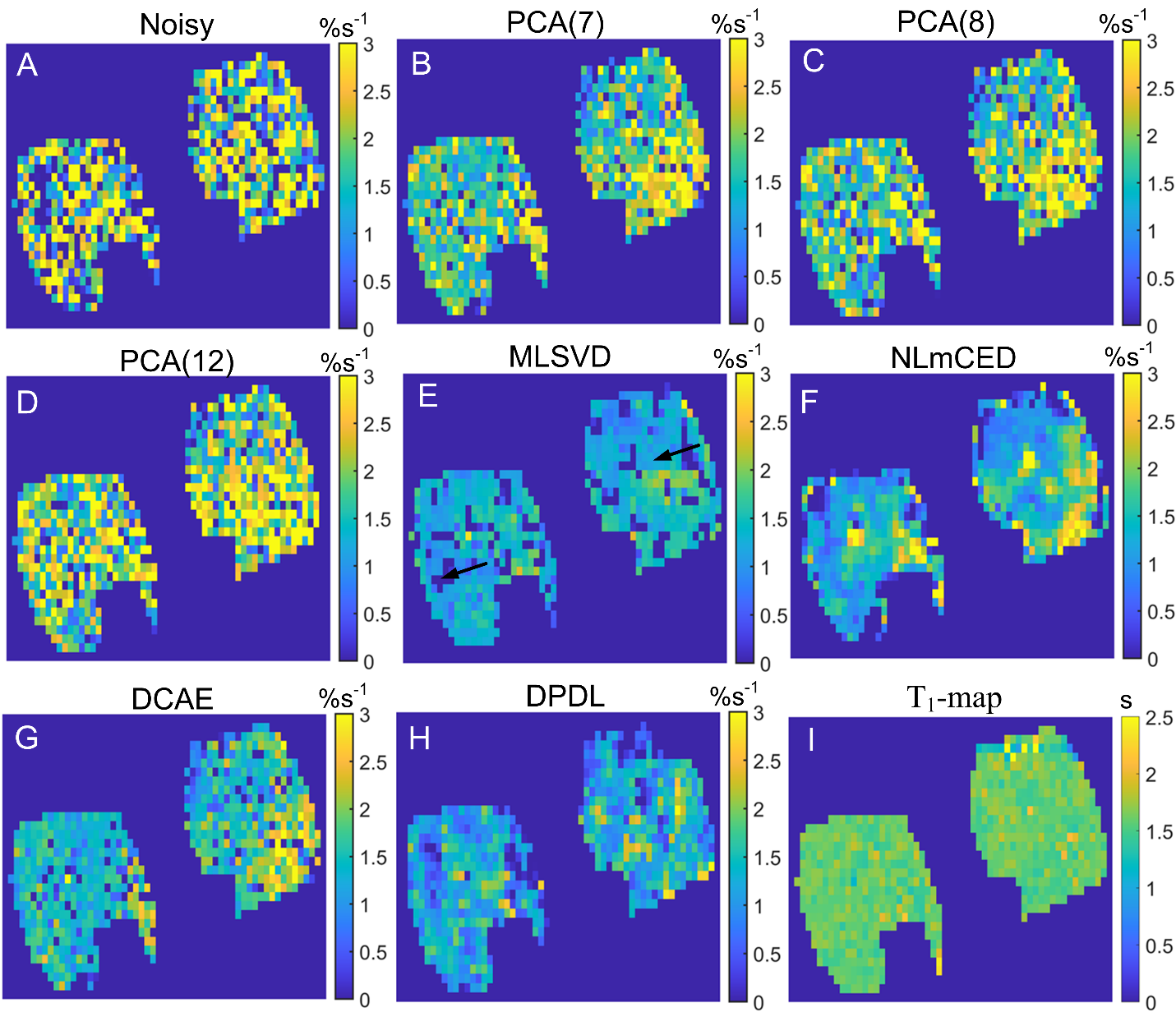


**Supporting information Fig.S40:** LD-fitted APT maps from the leg muscle in a rat (#2), without denoising (A) and with denoising by PCA(7) (B), PCA(8) (C), PCA(12) (D), MLSVD (E), NLmCED (F), DCAE (G), and DPDL (H). T_1_ map was shown in (I). Arrows in (E) point to patches of uniform intensity, highlighting the suboptimal performance of the denoising.


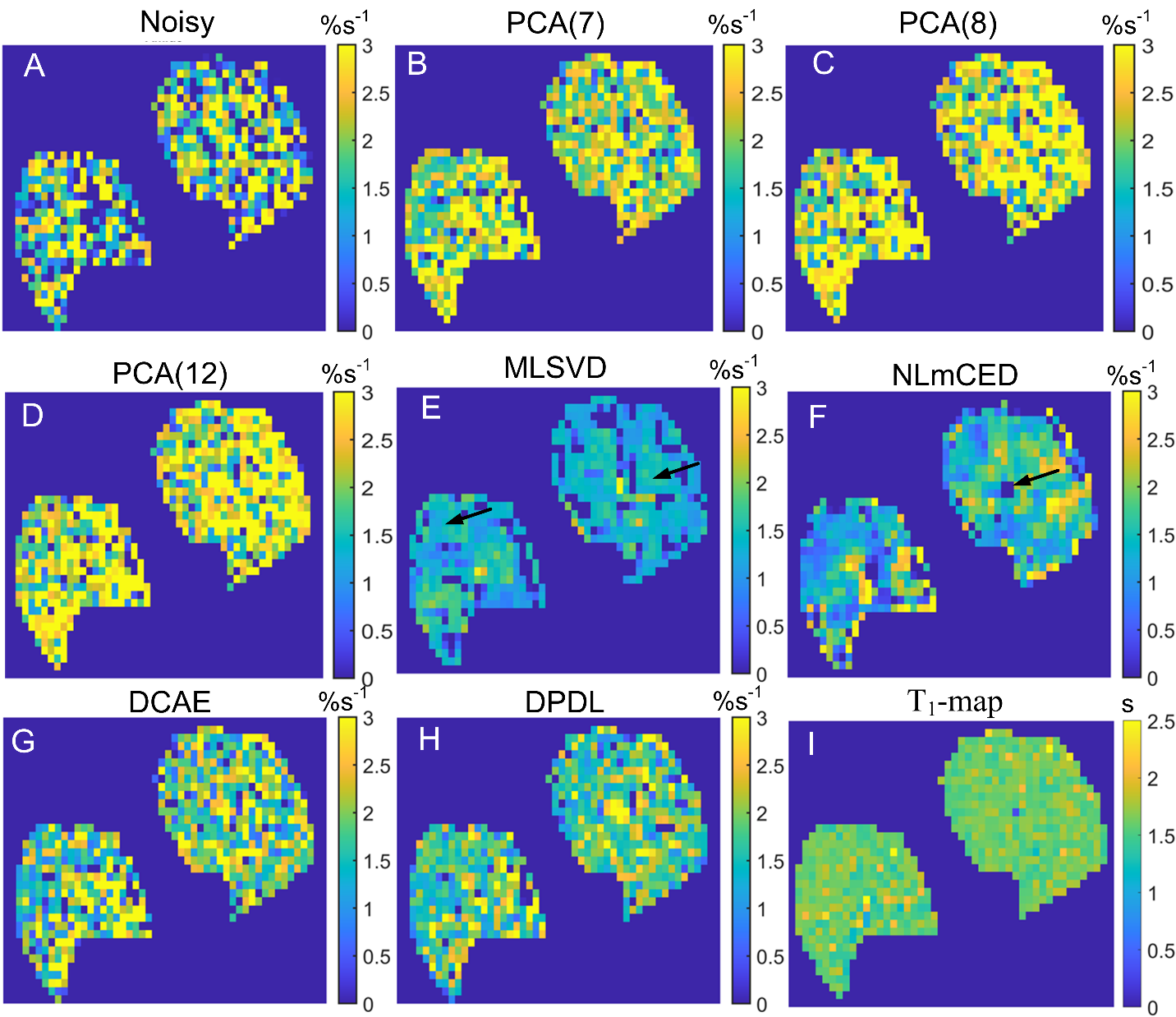


**Supporting information Fig.S41:** LD-fitted APT maps from the leg muscle in a rat (#3), without denoising (A) and with denoising by PCA(7) (B), PCA(8) (C), PCA(12) (D), MLSVD (E), NLmCED (F), DCAE (G), and DPDL (H). T_1_ map was shown in (I). Arrows in (E) and (F) point to patches of uniform intensity, highlighting the suboptimal performance of the denoising.


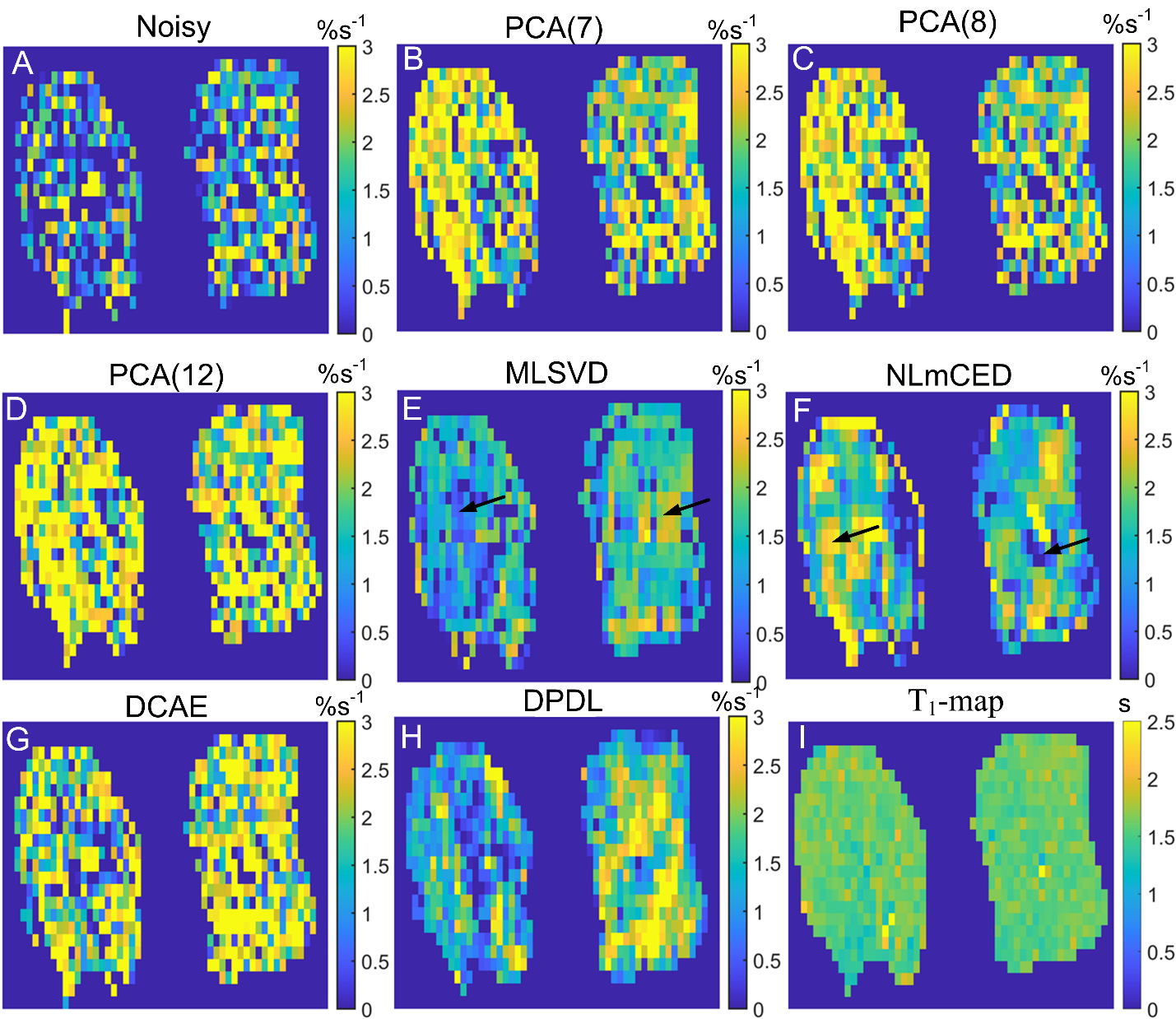


**Supporting information Fig.S42:** LD-fitted APT maps from the leg muscle in a rat (#4), without denoising (A) and with denoising by PCA(7) (B), PCA(8) (C), PCA(12) (D), MLSVD (E), NLmCED (F), DCAE (G), and DPDL (H). T_1_ map was shown in (I). Arrows in (E) and (F) point to patches of uniform intensity, highlighting the suboptimal performance of the denoising.


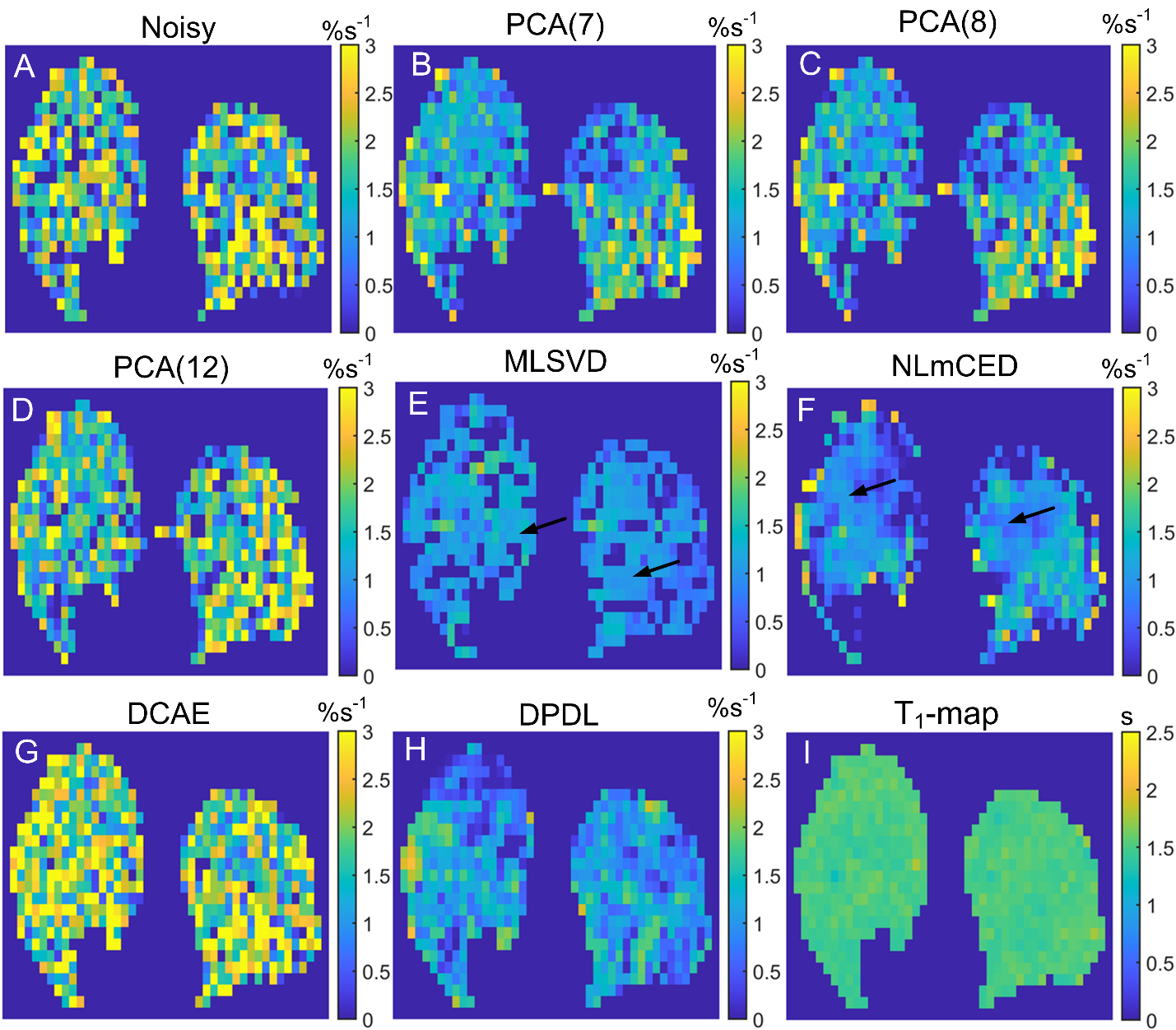


**Supporting information Fig.S43:** LD-fitted APT maps from the leg muscle in a rat (#5), without denoising (A) and with denoising by PCA(7) (B), PCA(8) (C), PCA(12) (D), MLSVD (E), NLmCED (F), DCAE (G), and DPDL (H). T_1_ map was shown in (I). Arrows in (E) and (F) point to patches of uniform intensity, highlighting the suboptimal performance of the denoising.


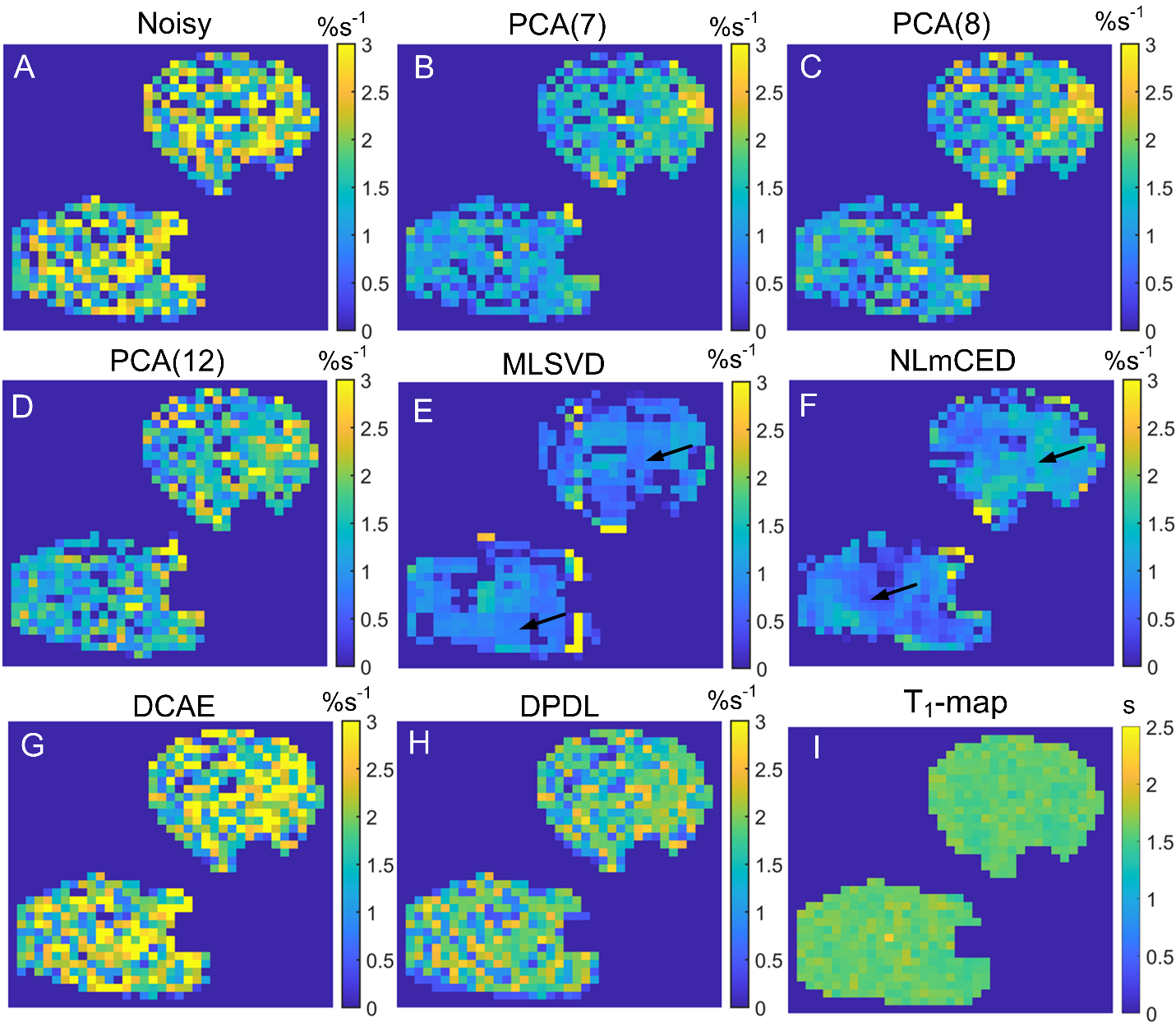


**Supporting information Fig.S44:** LD-fitted APT maps from the leg muscle in a rat (#6), without denoising (A) and with denoising by PCA(7) (B), PCA(8) (C), PCA(12) (D), MLSVD (E), NLmCED (F), DCAE (G), and DPDL (H). T_1_ map was shown in (I). Arrows in (E) and (F) point to patches of uniform intensity, highlighting the suboptimal performance of the denoising.


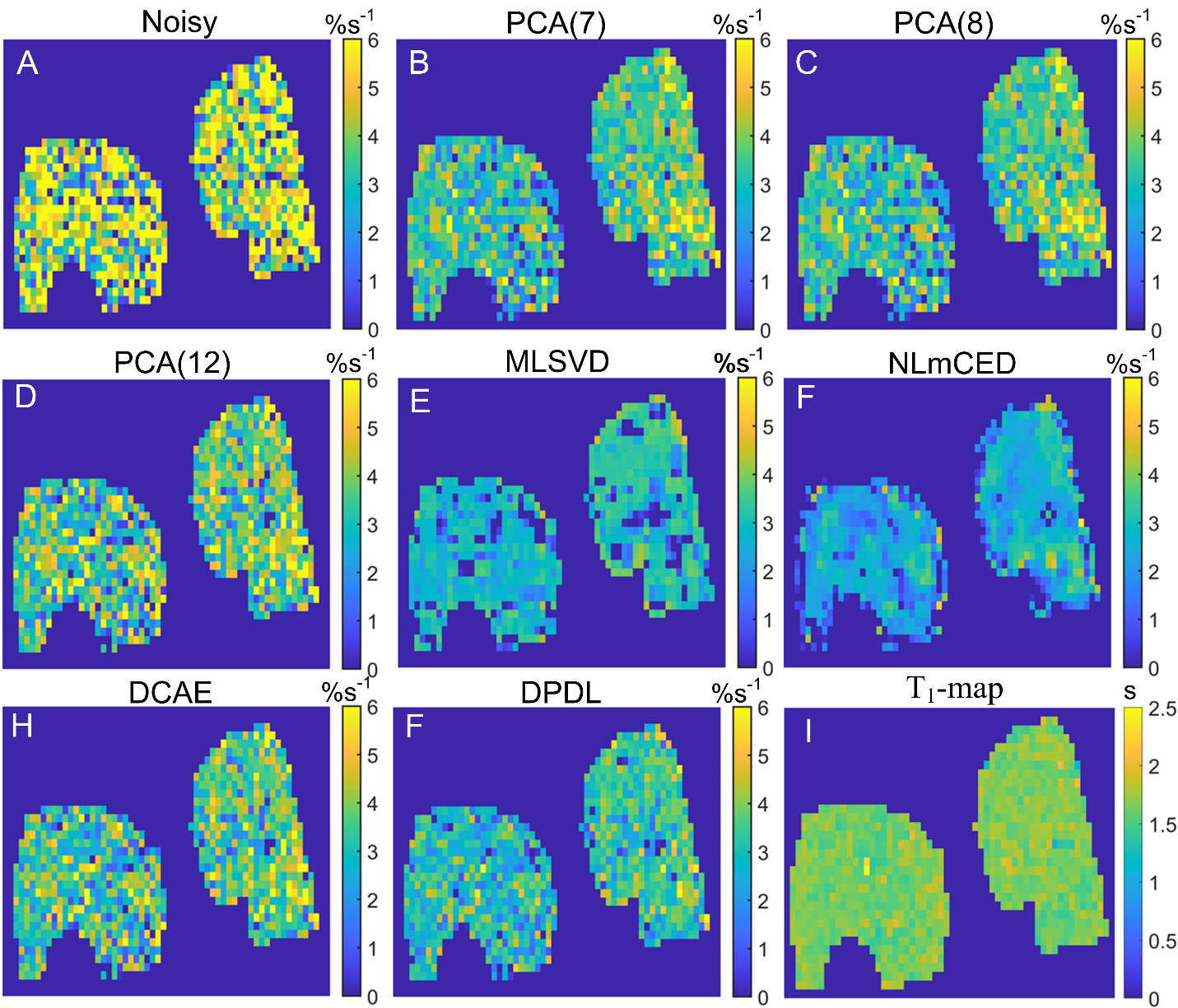


**Supporting information Fig.S45:** LD-fitted PCr maps from the leg muscle in a rat (#1), without denoising (A) and with denoising by PCA(7) (B), PCA(8) (C), PCA(12) (D), MLSVD (E), NLmCED (F), DCAE (G), and DPDL (H). T_1_ map was shown in (I).


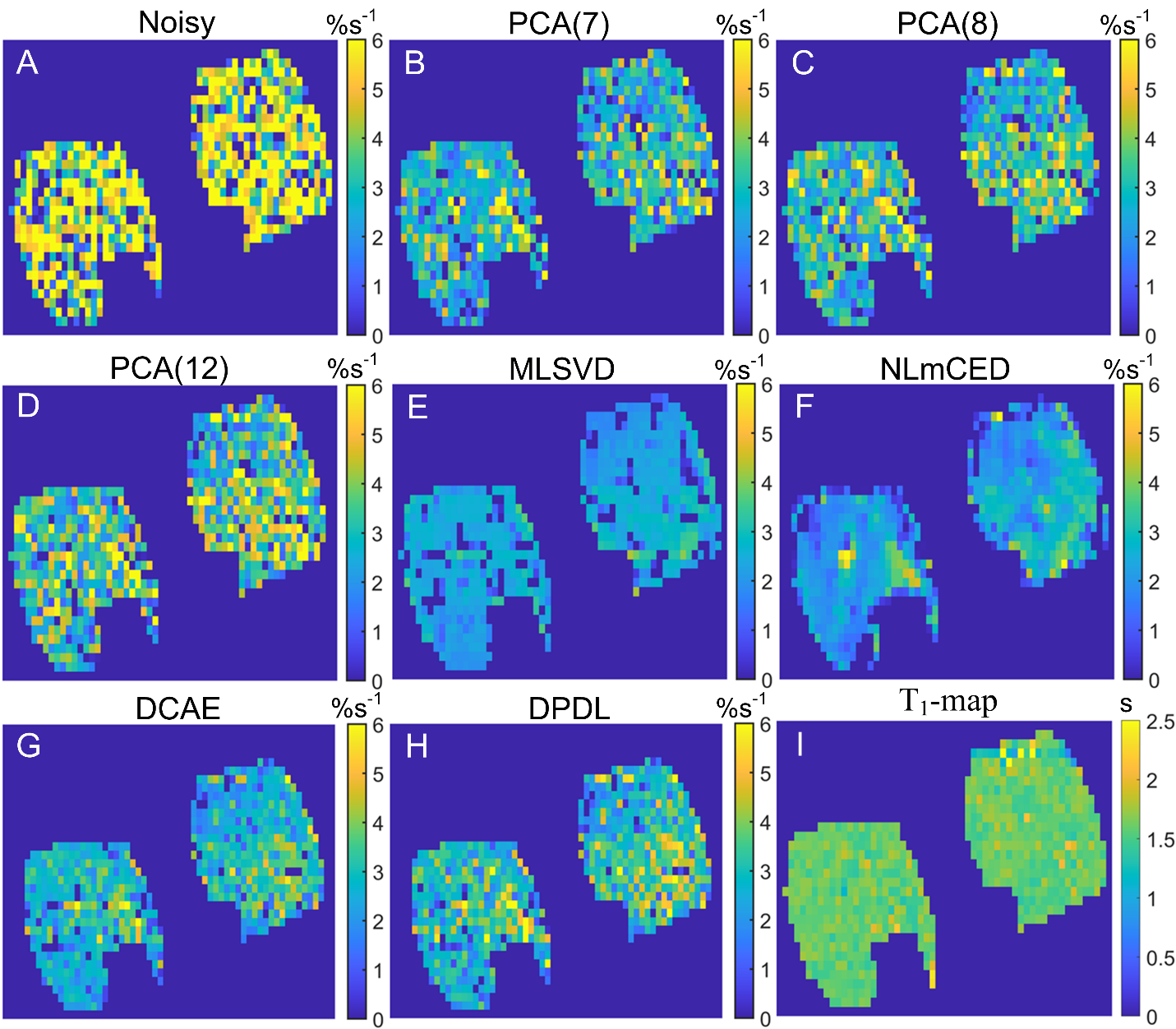


**Supporting information Fig.S46:** LD-fitted PCr maps from the leg muscle in a rat (#2), without denoising (A) and with denoising by PCA(7) (B), PCA(8) (C), PCA(12) (D), MLSVD (E), NLmCED (F), DCAE (G), and DPDL (H). T_1_ map was shown in (I).


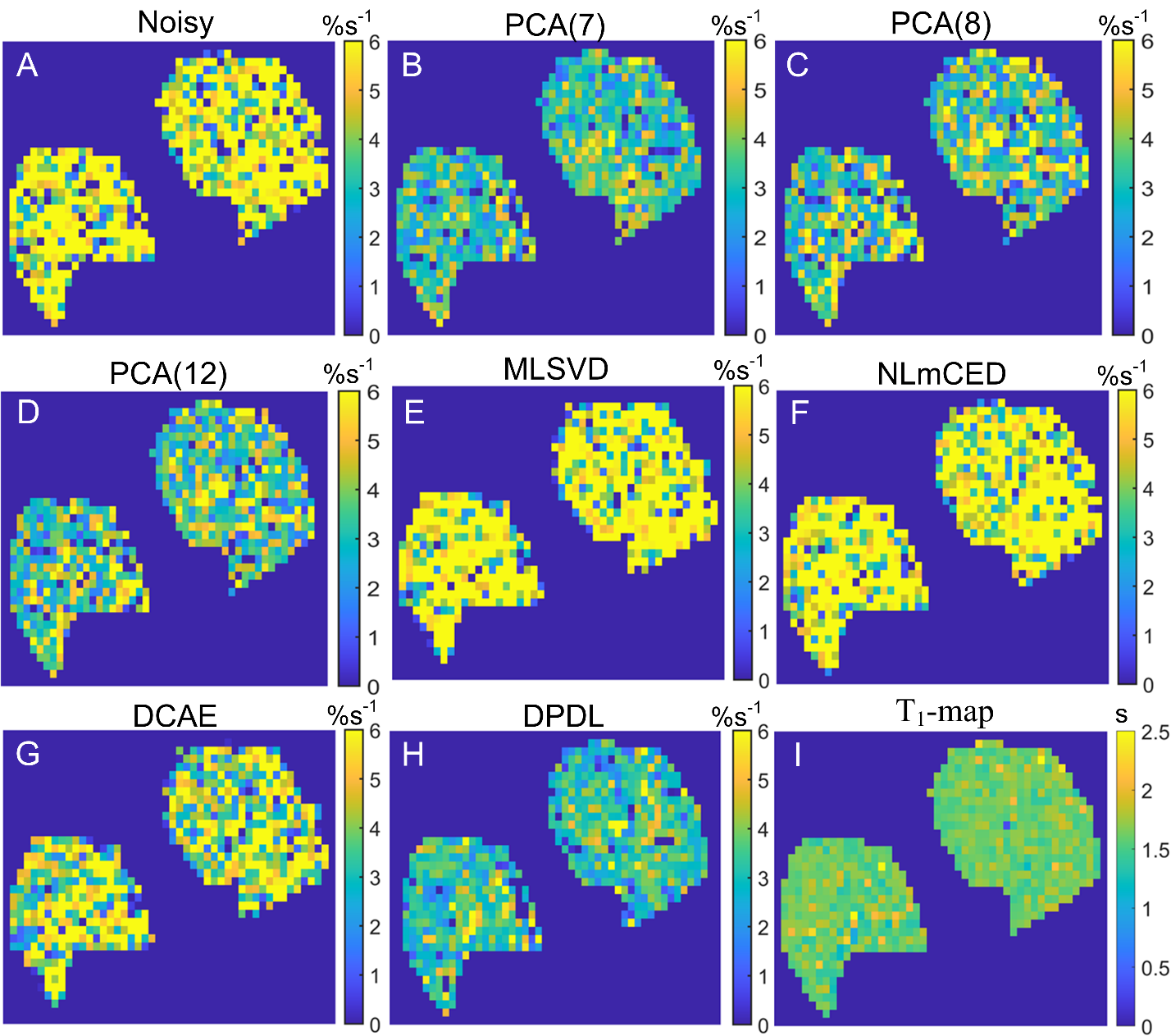


**Supporting information Fig.S47:** LD-fitted PCr maps from the leg muscle in a rat (#3), without denoising (A) and with denoising by PCA(7) (B), PCA(8) (C), PCA(12) (D), MLSVD (E), NLmCED (F), DCAE (G), and DPDL (H). T_1_ map was shown in (I).


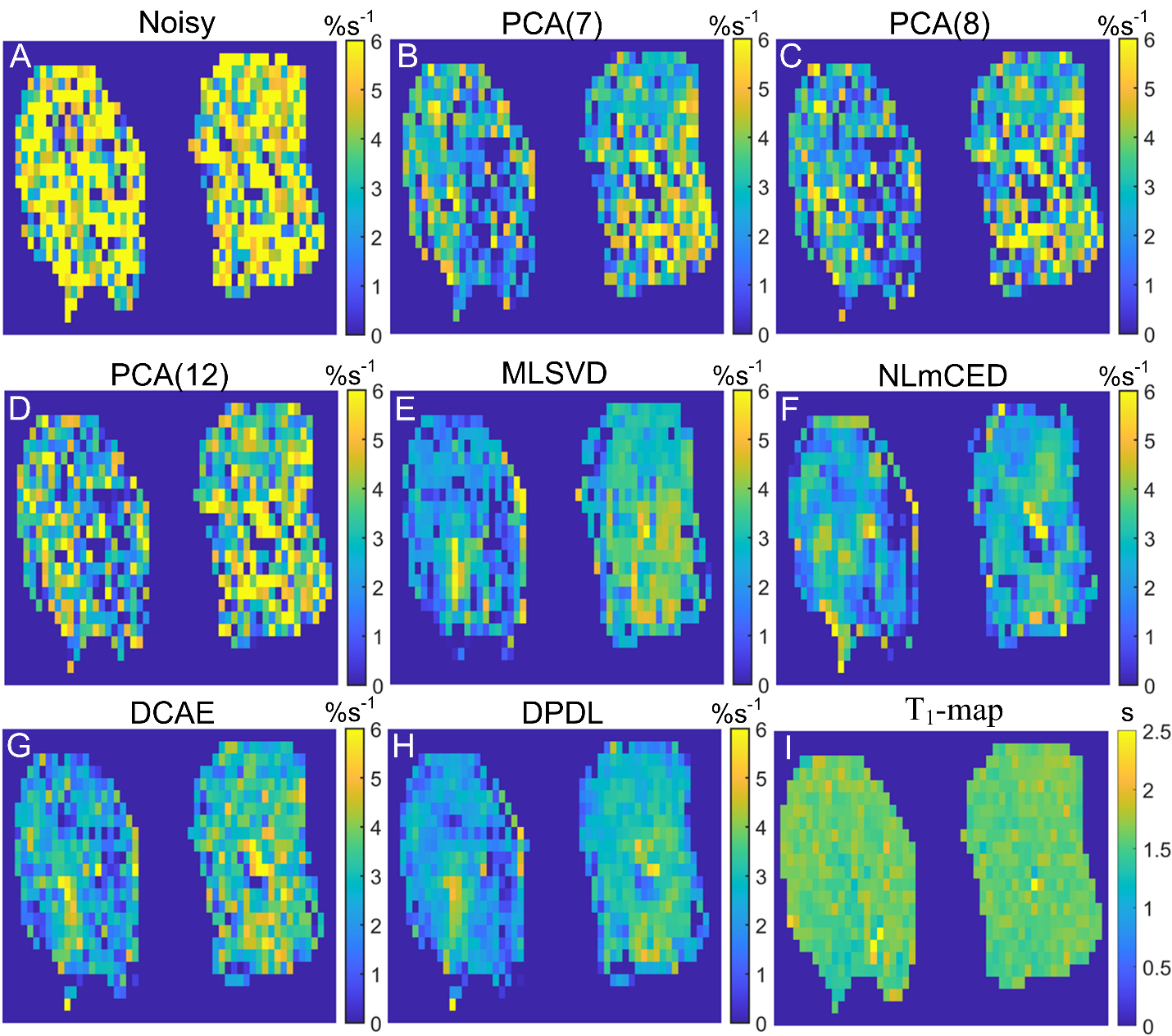


**Supporting information Fig.S48:** LD-fitted PCr maps from the leg muscle in a rat (#4), without denoising (A) and with denoising by PCA(7) (B), PCA(8) (C), PCA(12) (D), MLSVD (E), NLmCED (F), DCAE (G), and DPDL (H). T_1_ map was shown in (I).


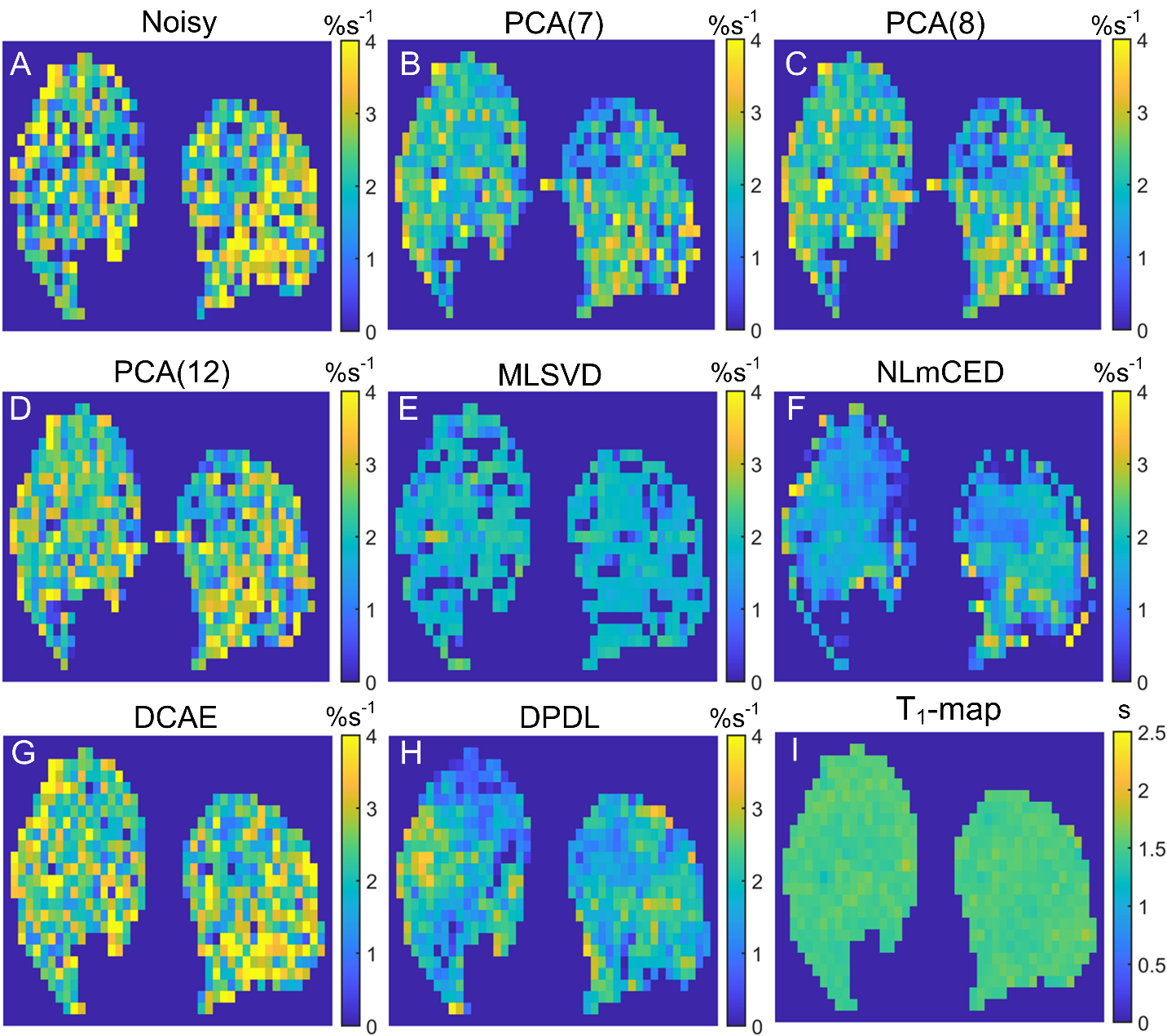


**Supporting information Fig.S49:** LD-fitted PCr maps from the leg muscle in a rat (#5), without denoising (A) and with denoising by PCA(7) (B), PCA(8) (C), PCA(12) (D), MLSVD (E), NLmCED (F), DCAE (G), and DPDL (H). T_1_ map was shown in (I).


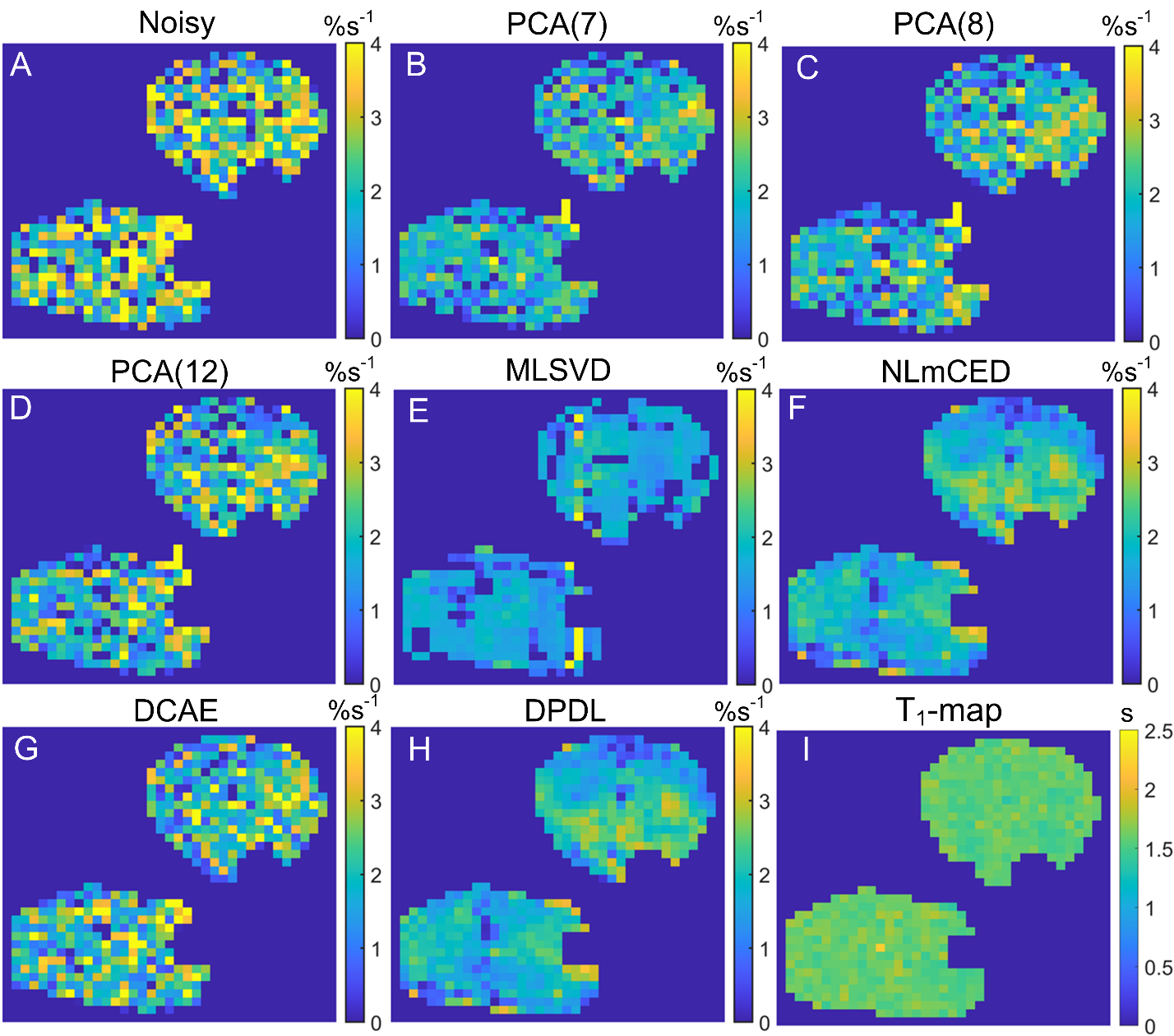


**Supporting information Fig.S50:** LD-fitted PCr maps from the leg muscle in a rat (#6), without denoising (A) and with denoising by PCA(7) (B), PCA(8) (C), PCA(12) (D), MLSVD (E), NLmCED (F), DCAE (G), and DPDL (H). T_1_ map was shown in (I).


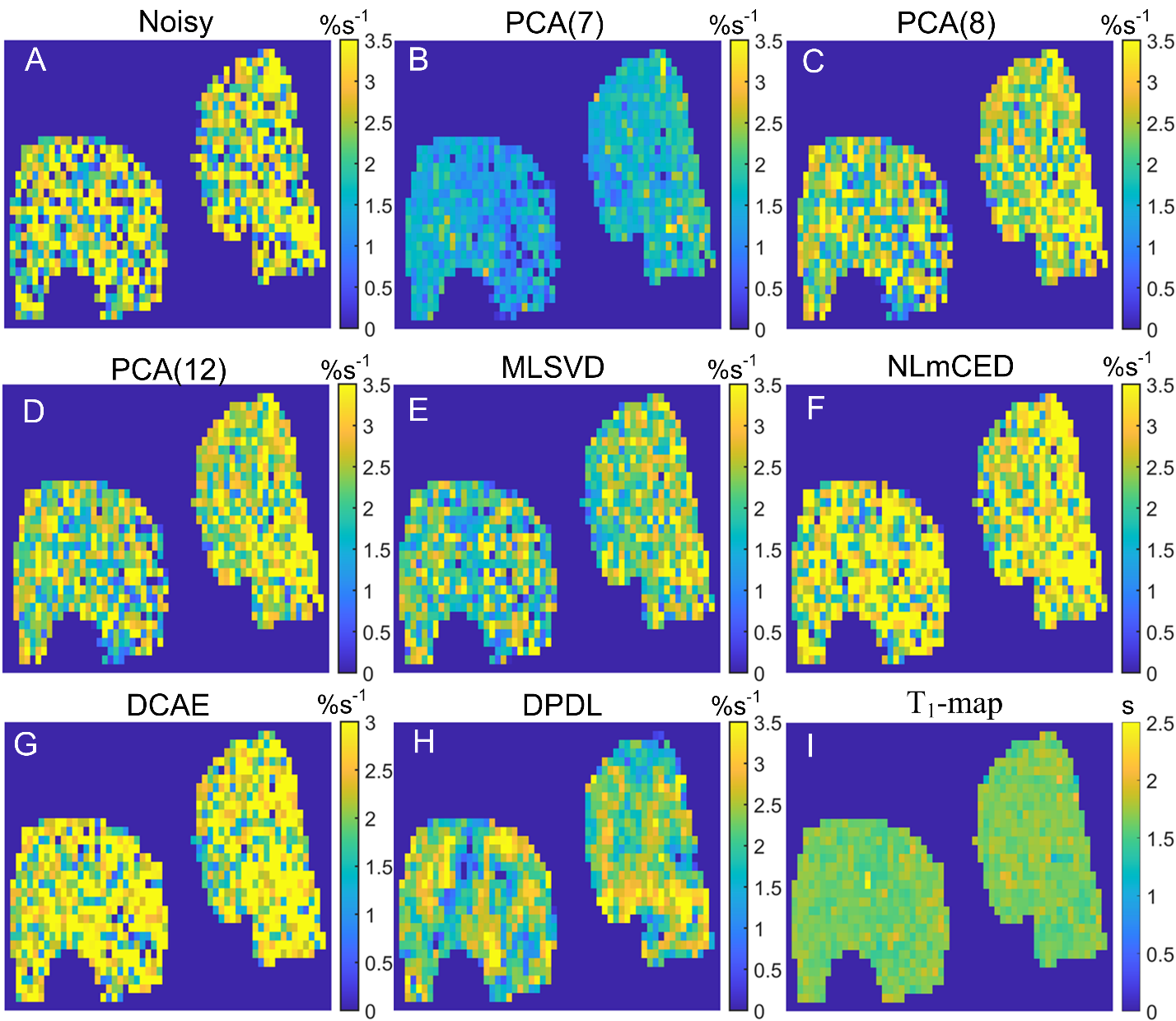


**Supporting information Fig.S51:** LD-fitted guanidine maps from the leg muscle in a rat (#1), without denoising (A) and with denoising by PCA(7) (B), PCA(8) (C), PCA(12) (D), MLSVD (E), NLmCED (F), DCAE (G), and DPDL (H). T_1_ map was shown in (I).


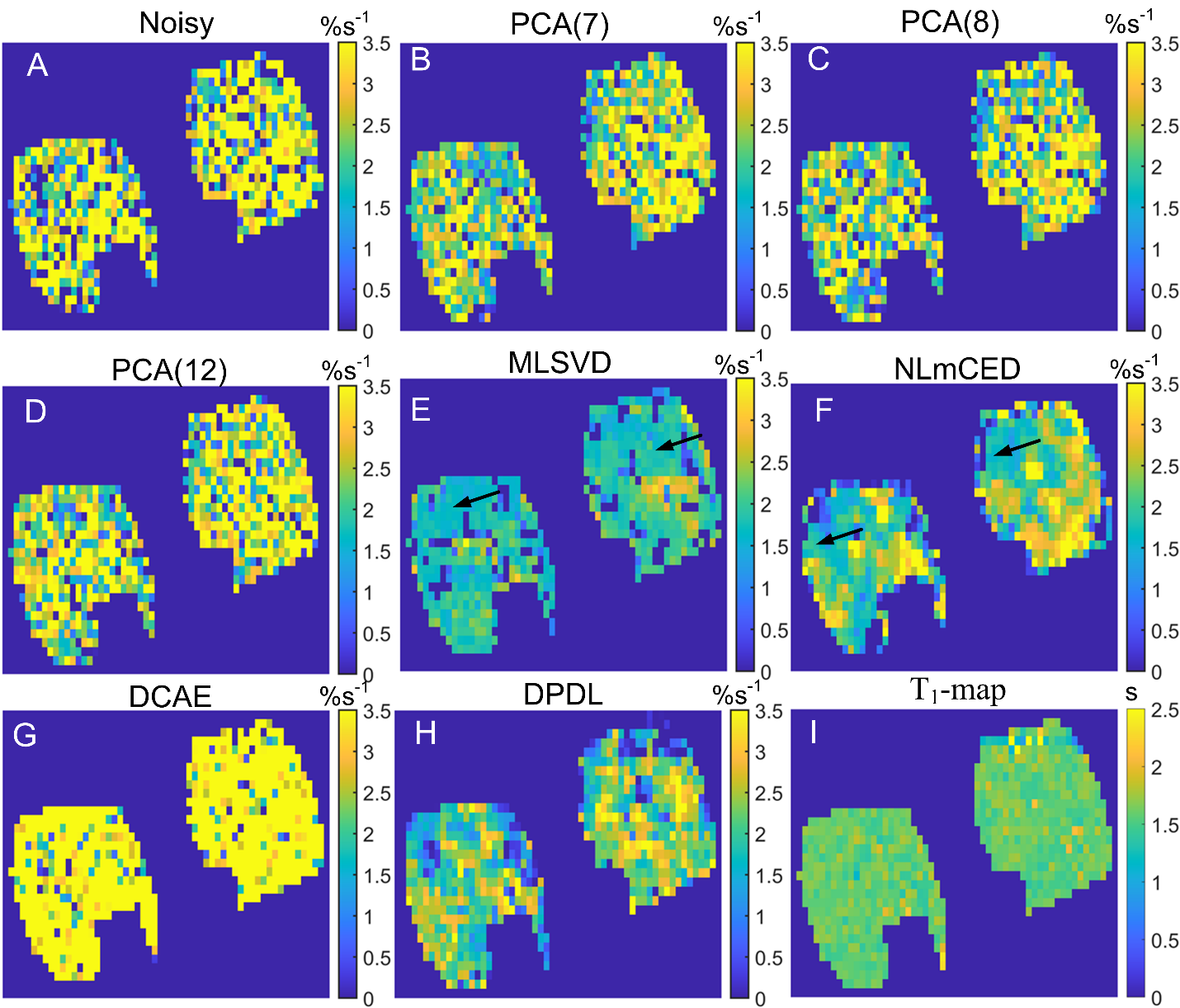


**Supporting information Fig.S52:** LD-fitted guanidine maps from the leg muscle in a rat (#2), without denoising (A) and with denoising by PCA(7) (B), PCA(8) (C), PCA(12) (D), MLSVD (E), NLmCED (F), DCAE (G), and DPDL (H). T_1_ map was shown in (I). Arrows in (E) and (F) point to patches of uniform intensity, highlighting the suboptimal performance of the denoising.


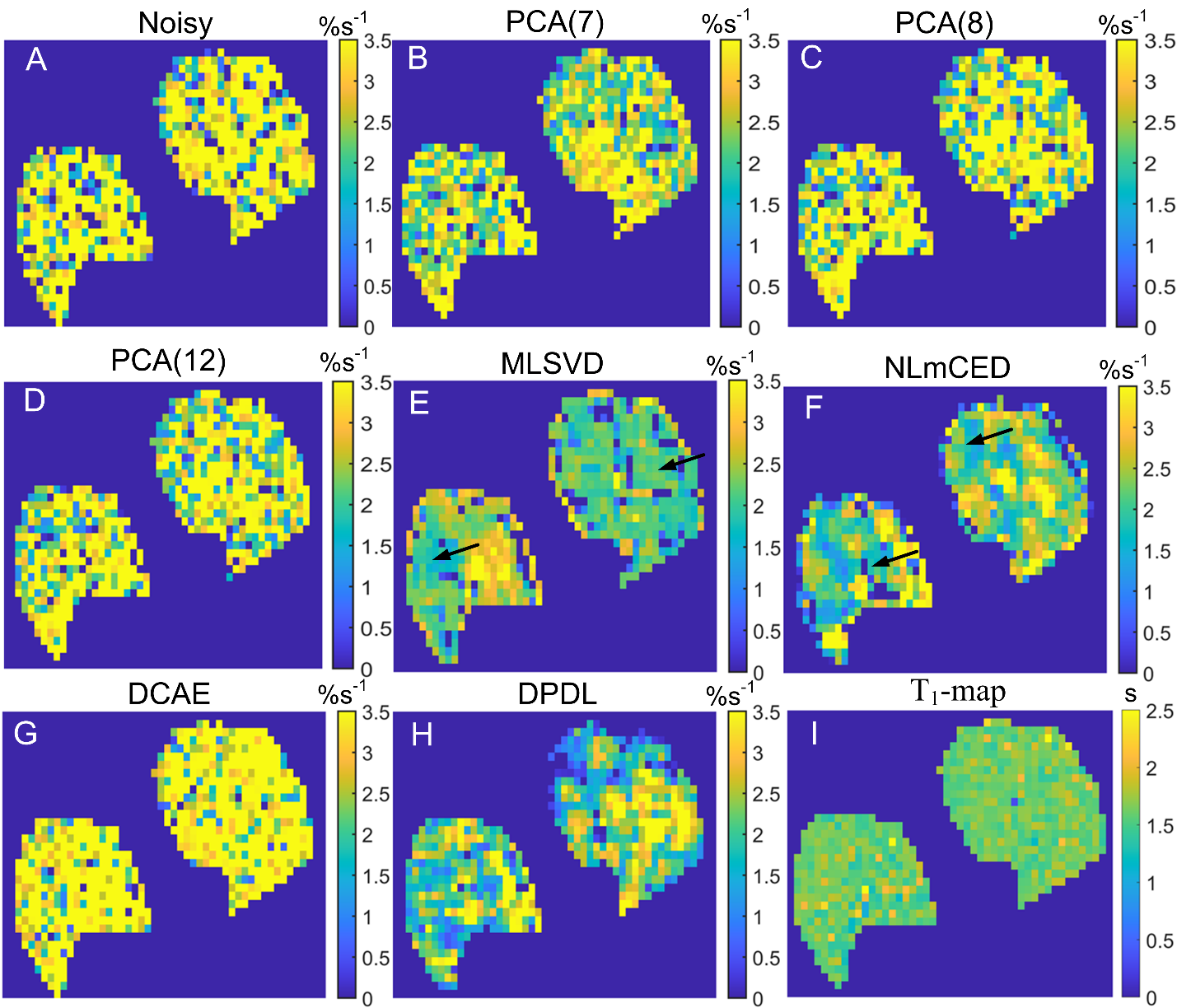


**Supporting information Fig.S53:** LD-fitted guanidine maps from the leg muscle in a rat (#3), without denoising (A) and with denoising by PCA(7) (B), PCA(8) (C), PCA(12) (D), MLSVD (E), NLmCED (F), DCAE (G), and DPDL (H). T_1_ map was shown in (I). Arrows in (E) and (F) point to patches of uniform intensity, highlighting the suboptimal performance of the denoising.


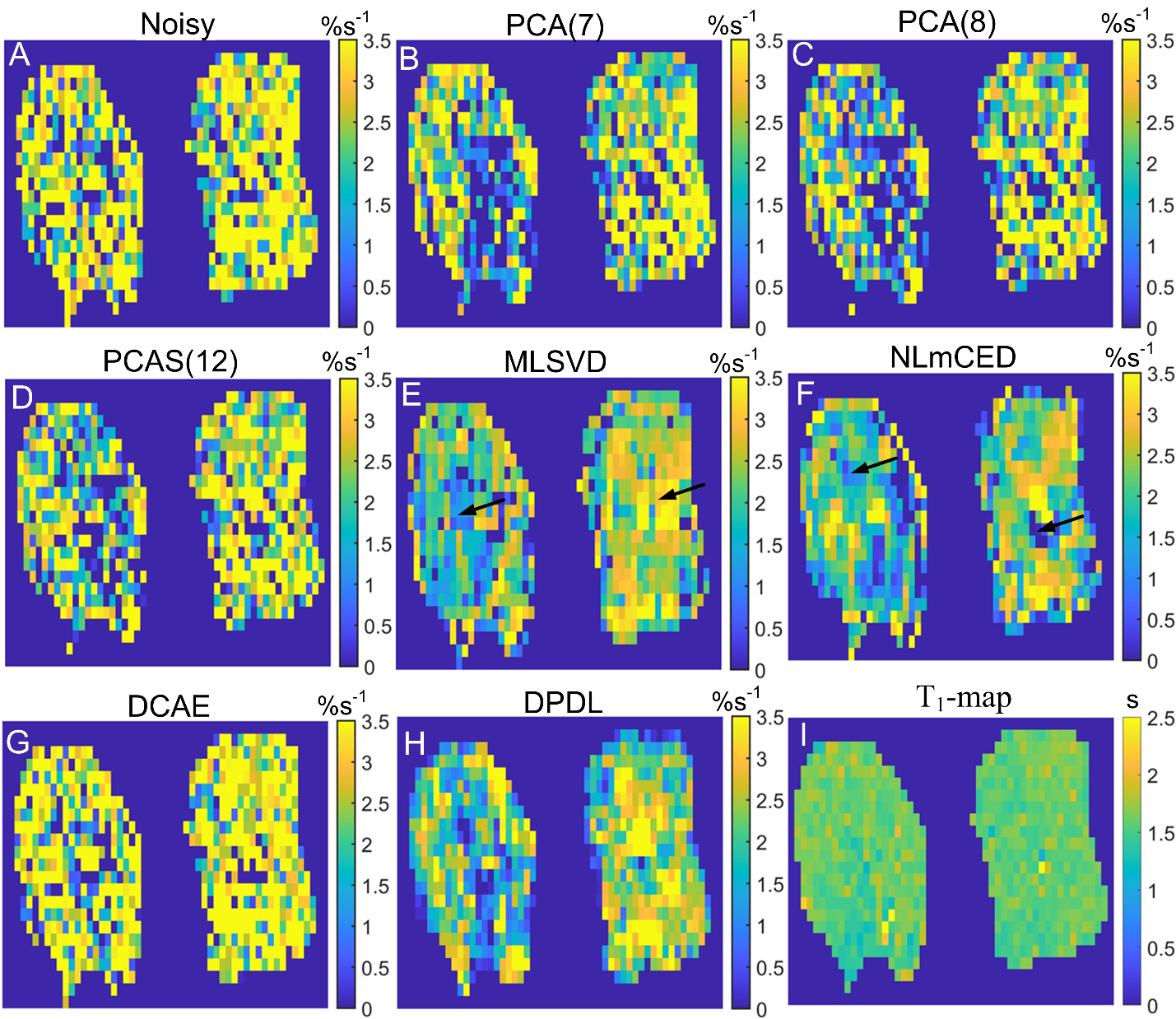


**Supporting information Fig.S54:** LD-fitted guanidine maps from the leg muscle in a rat (#4), without denoising (A) and with denoising by PCA(7) (B), PCA(8) (C), PCA(12) (D), MLSVD (E), NLmCED (F), DCAE (G), and DPDL (H). T_1_ map was shown in (I). Arrows in (E) and (F) point to patches of uniform intensity, highlighting the suboptimal performance of the denoising.


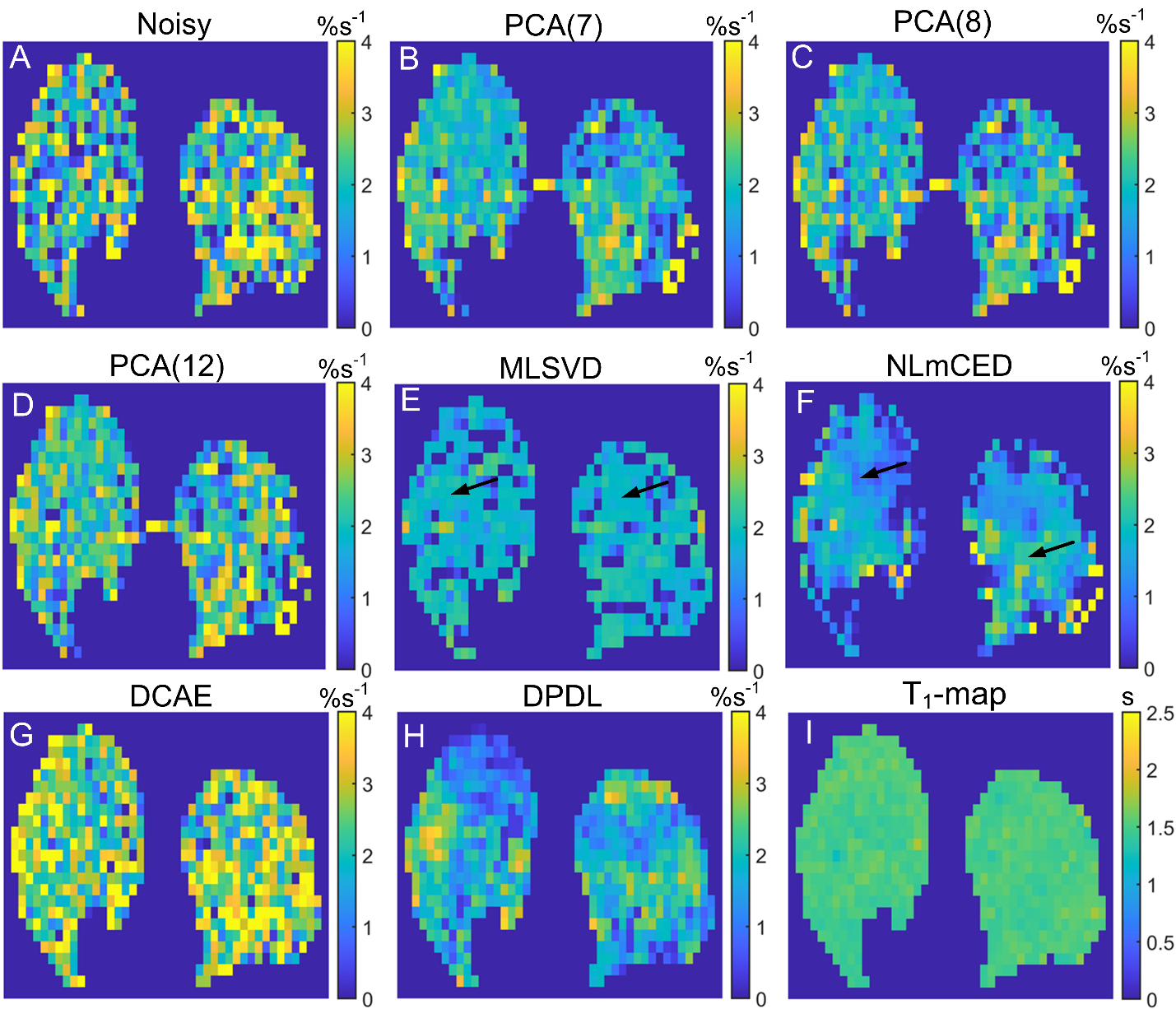


**Supporting information Fig.S55:** LD-fitted guanidine maps from the leg muscle in a rat (#5), without denoising (A) and with denoising by PCA(7) (B), PCA(8) (C), PCA(12) (D), MLSVD (E), NLmCED (F), DCAE (G), and DPDL (H). T_1_ map was shown in (I). Arrows in (E) and (F) point to patches of uniform intensity, highlighting the suboptimal performance of the denoising.


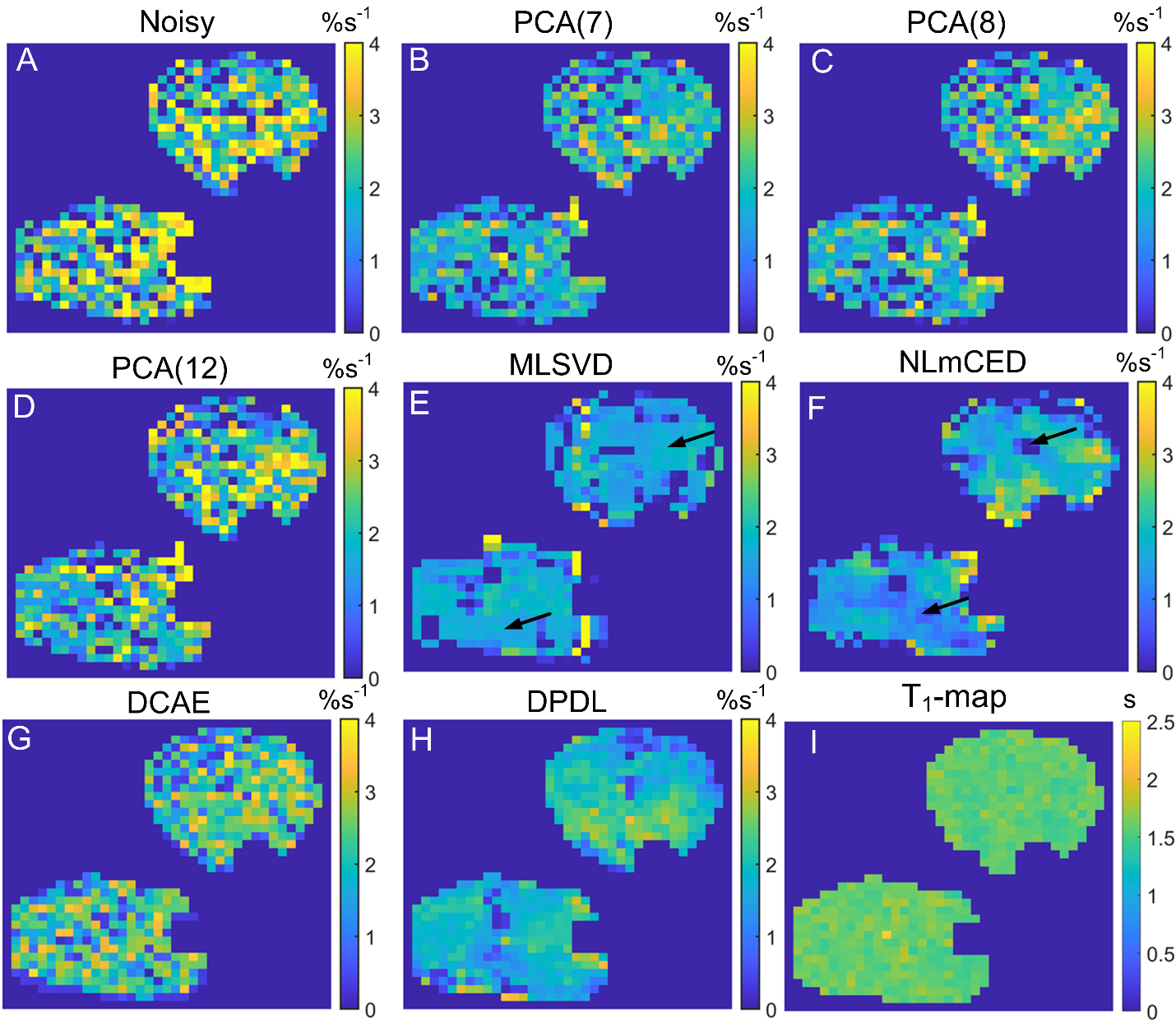


**Supporting information Fig.S56:** LD-fitted guanidine maps from the leg muscle in a rat (#6), without denoising (A) and with denoising by PCA(7) (B), PCA(8) (C), PCA(12) (D), MLSVD (E), NLmCED (F), DCAE (G), and DPDL (H). T_1_ map was shown in (I). Arrows in (E) and (F) point to patches of uniform intensity, highlighting the suboptimal performance of the denoising.


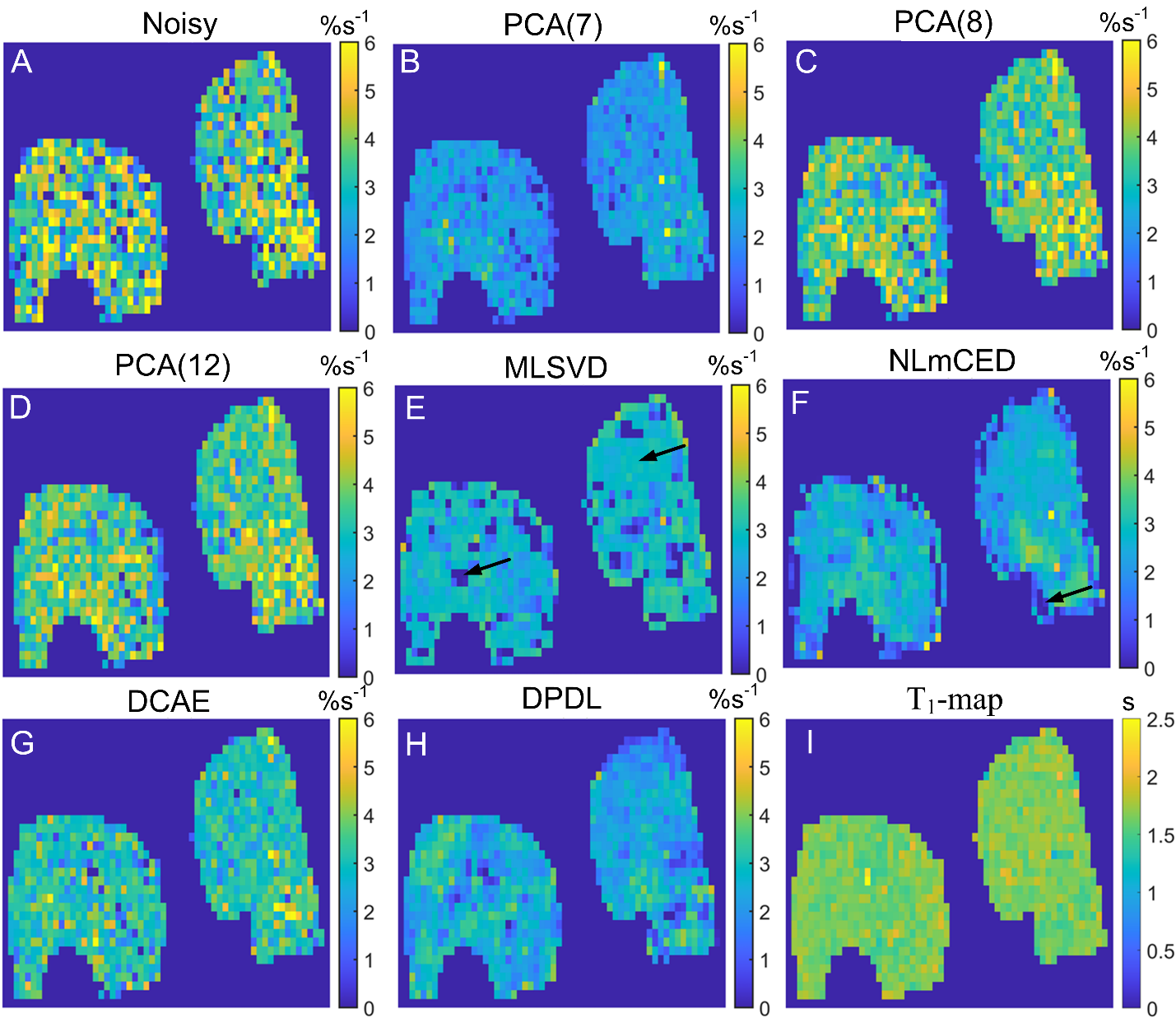


**Supporting information Fig.S57:** LD-fitted NOE(-3.5) maps from the leg muscle in a rat (#1), without denoising (A) and with denoising by PCA(7) (B), PCA(8) (C), PCA(12) (D), MLSVD (E), NLmCED (F), DCAE (G), and DPDL (H). T_1_ map was shown in (I). Arrows in (E) and (F) point to patches of uniform intensity, highlighting the suboptimal performance of the denoising.


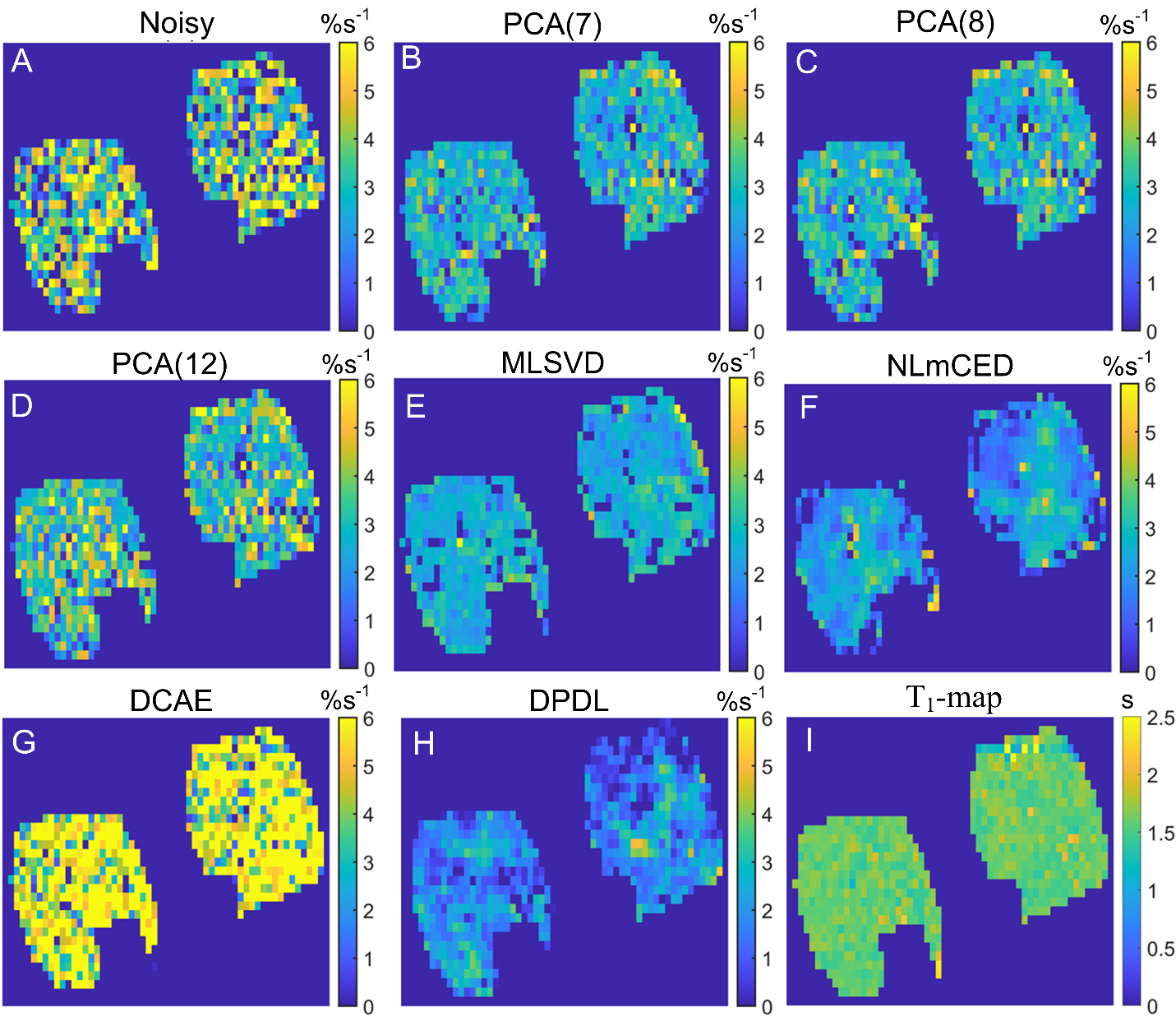


**Supporting information Fig.S58:** LD-fitted NOE(-3.5) maps from the leg muscle in a rat (#2), without denoising (A) and with denoising by PCA(7) (B), PCA(8) (C), PCA(12) (D), MLSVD (E), NLmCED (F), DCAE (G), and DPDL (H). T_1_ map was shown in (I).


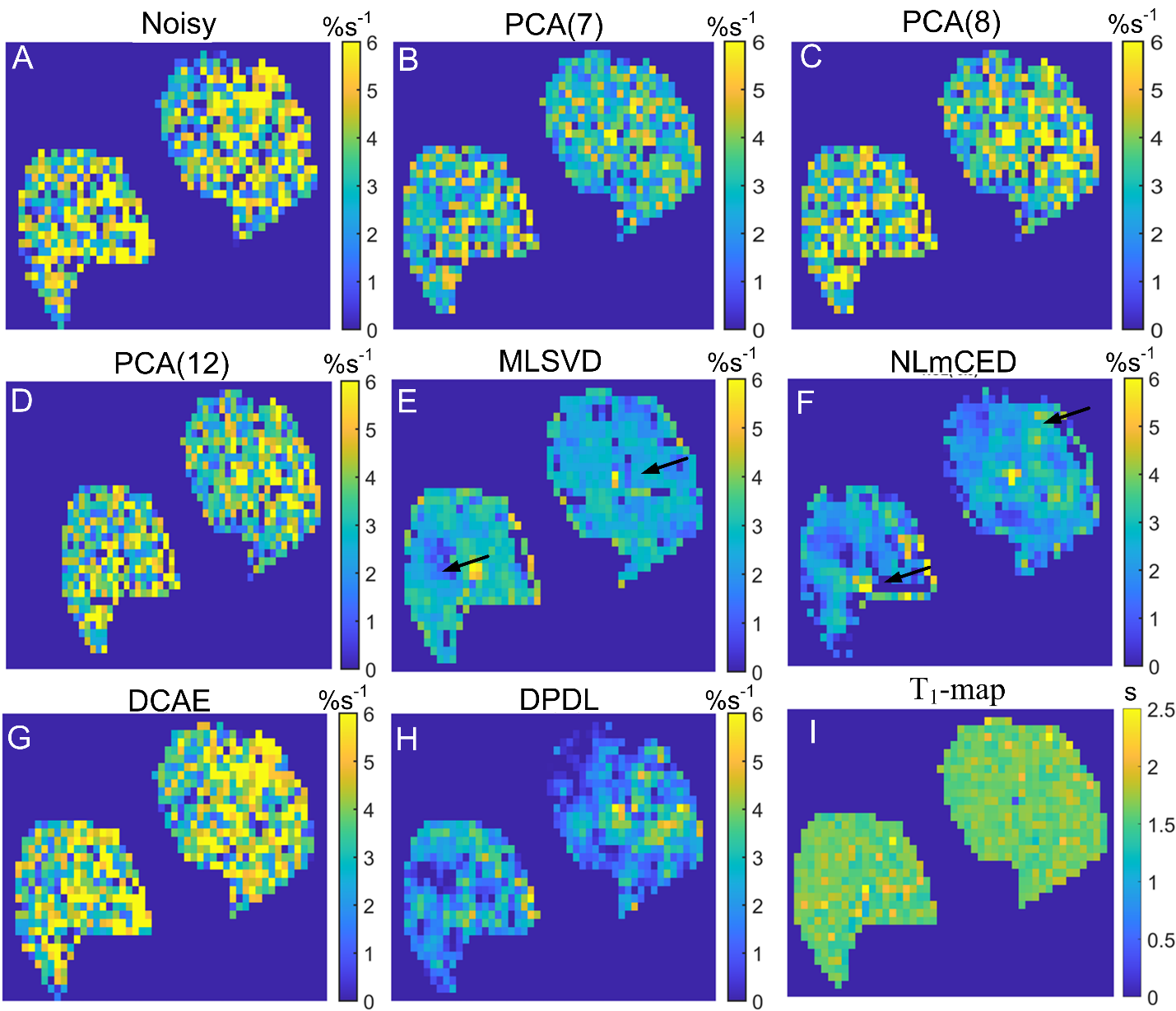


**Supporting information Fig.S59:** LD-fitted NOE(-3.5) maps from the leg muscle in a rat (#3), without denoising (A) and with denoising by PCA(7) (B), PCA(8) (C), PCA(12) (D), MLSVD (E), NLmCED (F), DCAE (G), and DPDL (H). T_1_ map was shown in (I). Arrows in (E) and (F) point to patches of uniform intensity, highlighting the suboptimal performance of the denoising.


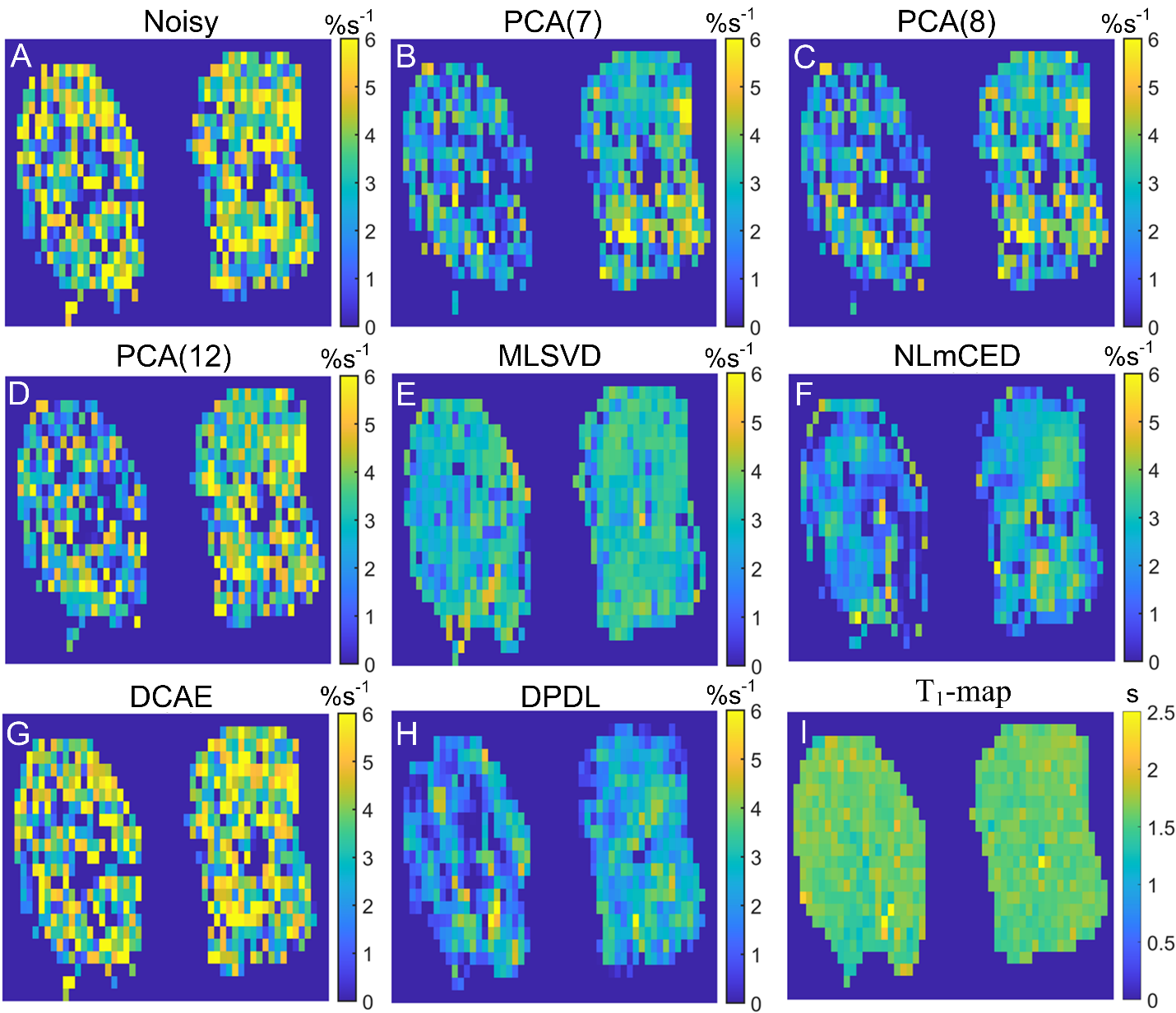


**Supporting information Fig.S60:** LD-fitted NOE(-3.5) maps from the leg muscle in a rat (#4), without denoising (A) and with denoising by PCA(7) (B), PCA(8) (C), PCA(12) (D), MLSVD (E), NLmCED (F), DCAE (G), and DPDL (H). T_1_ map was shown in (I).


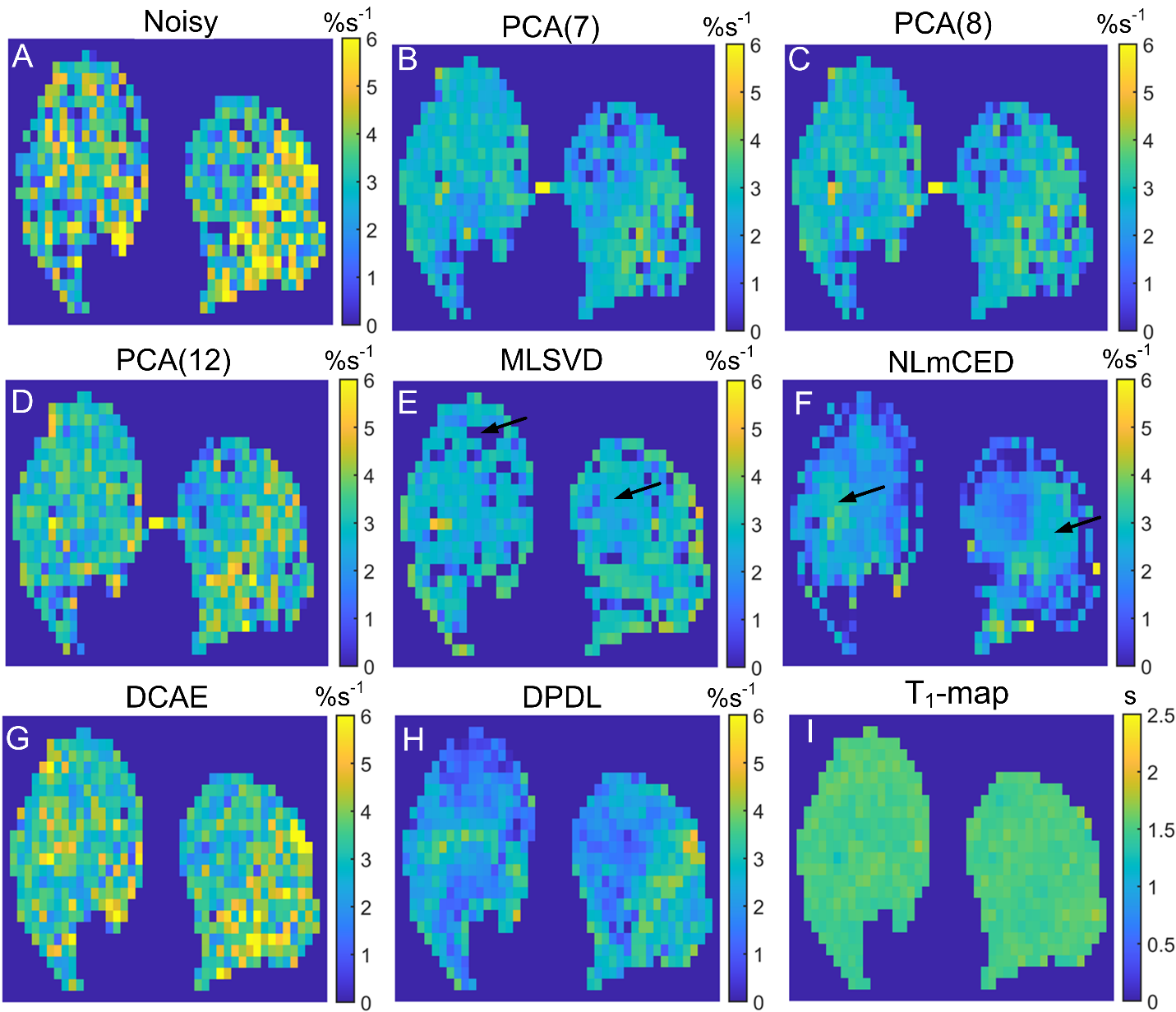


**Supporting information Fig.S61:** LD-fitted NOE(-3.5) maps from the leg muscle in a rat (#5), without denoising (A) and with denoising by PCA(7) (B), PCA(8) (C), PCA(12) (D), MLSVD (E), NLmCED (F), DCAE (G), and DPDL (H). T_1_ map was shown in (I). Arrows in (E) and (F) point to patches of uniform intensity, highlighting the suboptimal performance of the denoising.


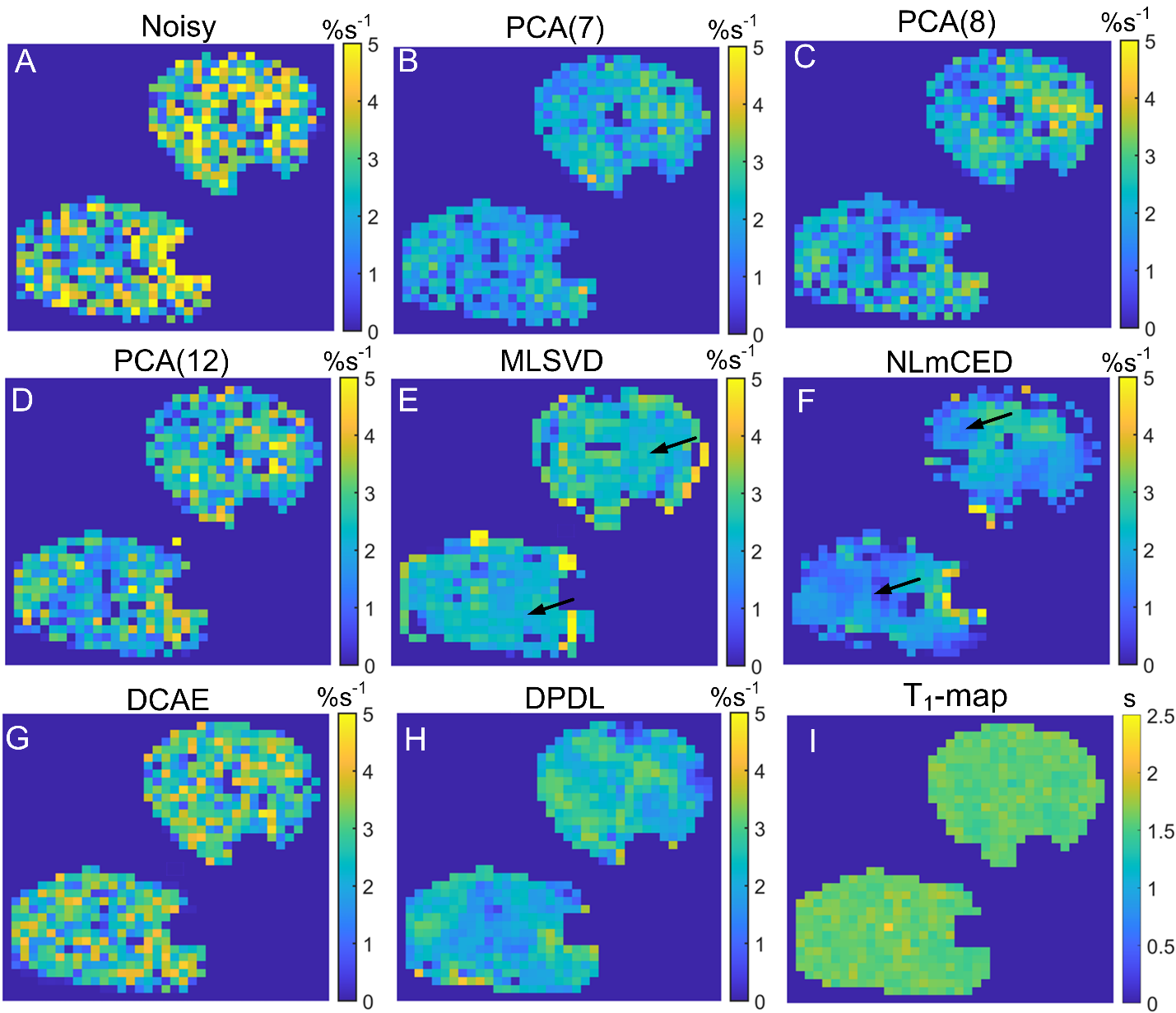


**Supporting information Fig.S62:** LD-fitted NOE(-3.5) maps from the leg muscle in a rat (#6), without denoising (A) and with denoising by PCA(7) (B), PCA(8) (C), PCA(12) (D), MLSVD (E), NLmCED (F), DCAE (G), and DPDL (H). T_1_ map was shown in (I). Arrows in (E) and (F) point to patches of uniform intensity, highlighting the suboptimal performance of the denoising.

1. Zhou W, Bovik AC, Sheikh HR, Simoncelli EP. Image quality assessment: from error visibility to structural similarity. IEEE Transactions on Image Processing 2004;13(4):600-612.

2. Cui J, Zhao Y, Sun C, Xu J, Zu Z. Evaluation of contributors to amide proton transfer-weighted imaging and nuclear Overhauser enhancement-weighted imaging contrast in tumors at a high magnetic field. Magn Reson Med 2023;90(2):596-614.

3. Cohen O, Yu VY, Tringale KR, Young RJ, Perlman O, Farrar CT, Otazo R. CEST MR fingerprinting (CEST-MRF) for brain tumor quantification using EPI readout and deep learning reconstruction. Magn Reson Med 2023;89(1):233-249.

4. Heo HY, Han Z, Jiang SS, Schar M, van Zijl PCM, Zhou JY. Quantifying amide proton exchange rate and concentration in chemical exchange saturation transfer imaging of the human brain. Neuroimage 2019;189:202-213.

5. Cai K, Haris M, Singh A, Kogan F, Greenberg JH, Hariharan H, Detre JA, Reddy R. Magnetic resonance imaging of glutamate. Nature medicine 2012;18(2):302-306.

6. Haris M, Nanga RPR, Singh A, Cai K, Kogan F, Hariharan H, Reddy R. Exchange rates of creatine kinase metabolites: feasibility of imaging creatine by chemical exchange saturation transfer MRI. NMR in biomedicine 2012;25(11):1305-1309.

7. Xiaocan Li and Shuo Wang and Yinghao C. Tutorial: Complexity analysis of Singular Value Decomposition and its variants. arXiv: Numerical Analysis 2019.

8. Cheng C-CaCF-CaHS-CaCB-H. Integral non-local means algorithm for image noise suppression. Electronics Letters 2015;51(19):1494-1495.

9. Freire PaSSaSBaNAaCNaPJEaTSK. Computational Complexity Optimization of Neural Network-Based Equalizers in Digital Signal Processing: A Comprehensive Approach. Journal of Lightwave Technology 2024;42(12):4177-4201.
